# In Vitro Characterization of Inhibitors for Lung A549 and Leukemia K562 Cell Lines from Fungal Transformation of Arecoline Supported by In Silico Docking to M3-mAChR and ADME Prediction

**DOI:** 10.3390/ph15101171

**Published:** 2022-09-21

**Authors:** Amany E. Ragab, Ebtisam T. Badawy, Shaimaa M. Aboukhatwa, Amal Kabbash, Kamilia A. Abo El-Seoud

**Affiliations:** 1 Department of Pharmacognosy, Faculty of Pharmacy, Tanta University, Tanta 31527, Egypt; 2 Department of Pharmaceutical Chemistry, Faculty of Pharmacy, Tanta University, Tanta 31527, Egypt; 3 Department of Pharmaceutical Sciences, College of Pharmacy, University of Illinois at Chicago, Chicago, IL 60612, USA

**Keywords:** arecoline, fungal transformation, lung A549, leukemia K562, apoptosis, M3-mAChR

## Abstract

The search for anticancer drugs is of continuous interest. Arecoline is an alkaloid with anticancer activity. Herein, the metabolism of arecoline through fungal transformation was investigated for the discovery of potential anticancer drugs with higher activity and selectivity. Compounds **1**–**5** were isolated, and their structures were fully elucidated using various spectroscopic analyses, including 1D and 2D NMR, ESIMS, and HRESIMS. This is the first report for the isolation of compounds **1** and **2**. An MTT assay was performed to determine the cytotoxic activity of arecoline and its metabolites in vitro using non-small-cell lung cancer A549 and leukemia K562 cell lines compared to staurosporine and doxorubicin as positive controls. For the non-small-cell lung A549 cell line, arecoline hydrobromide, staurosporine, and doxorubicin resulted in IC_50_ values of 11.73 ± 0.71 µM, 10.47 ± 0.64 µM, and 5.05 ± 0.13 µM, respectively, while compounds **1**, **3,** and **5** exhibited IC_50_ values of 3.08 ± 0.19 µM, 7.33 ± 0.45 µM, and 3.29 ± 0.20 µM, respectively. For the leukemia K562 cell line, the IC_50_ values of arecoline hydrobromide, staurosporine, and doxorubicin were 15.3 ± 1.08 µM, 5.07 ± 0.36 µM, and 6.94 ± 0.21 µM, respectively, while the IC_50_ values of compounds **1**, **3** and **5** were 1.56 ± 0.11 µM, 3.33 ± 0.24 µM, and 2.15 ± 0.15 µM, respectively. The selectivity index value of these compounds was higher than 3. These results indicated that compounds **1**, **3**, and **5** are very strong cytotoxic agents with higher activity than the positive controls and good selectivity toward the tested cancer cell lines. Cell cycle arrest was then studied by flow cytometry to investigate the apoptotic mechanism. Docking simulation revealed that most compounds possessed good binding poses and favorable protein-ligand interactions with muscarinic acetylcholine receptor M3-mAChR protein. In silico study of pharmacokinetics using SwissADME predicted compounds **1**–**5** to be drug-like with a high probability of good oral bioavailability.

## 1. Introduction

Arecoline is a psychoactive alkaloid isolated from the nuts of *Areca catechu* L., family Arecaceae, which is endemic to Southeast and South Asia. In addition to arecoline, areca nut contains the alkaloids guvacoline, arecaidine, and guvacine [1,2,3].

Due to its partial agonist activity on the nicotinic and muscarinic acetylcholine receptors (mAChRs), arecoline exerts a variety of effects on the central nervous system, such as alertness, stimulation, euphoria, and anxiolytic action [3].

Furthermore, arecoline exhibits anticancer activity. It causes apoptosis in EAHY 926 human endothelial cells, a hybridoma of human endothelium, and lung adenocarcinoma A549/8 [4]. Moreover, the death of human leukemia K562 cells was induced by arecoline. This effect was associated with the upregulation of tumor necrosis factor receptor-2 surface expression mediated by its muscarinic effect [5]. Another study suggested that arecoline prevents tumorigenesis of basal cell carcinoma by reducing levels of interleukin (IL-6), increasing levels of p53, which is a tumor suppressor factor, and finally eliciting cell cycle arrest and apoptosis [6]. In contrast, arecoline causes mutation of the oral mucosal cells resulting in oral cancer in areca nut chewers [7,8].

We anticipate that the cytotoxic effects of arecoline could be mediated through its action on muscarinic receptors. Muscarinic receptors are expressed in numerous primary and metastatic tumors, such as those in prostate, ovary, colon, and lung tissues [9,10,11]. In some of these tumors, such as small cell lung carcinoma, acetylcholine (Ach) is possibly synthesized by tumor cells, suggesting that Ach’s potential to affect tumor cell proliferation and migration might be dependent on an autocrine pathway mediated by the nicotinic and/or muscarinic receptors [12].

The ability of the antagonists of M3-muscarinic receptors to inhibit tumor growth has been revealed in colon and lung cancers. M3-muscarinic receptor antagonists inhibit the growth of small cell lung carcinoma [13]. On the other hand, muscarinic agonists produce cytotoxic actions in a dose-dependent manner [14]. Additionally, in human glioblastoma, the M2-mAChR agonist arecaidine inhibited cell proliferation and survival in cancer stem cells [15]. It was also reported that cholinergic receptors M3-mAChR play a significant role in the proliferation, differentiation, and apoptosis of leukemia cells [16].

A molecular docking simulation of arecoline revealed that it might bind to six sub-types of mAChR, most favorably to M3-mAChR [17].

The metabolic pathway of arecoline was studied in mice. Arecoline was subjected to *N*-oxidation to give arecoline *N*-oxide, and both compounds formed conjugates with mercapturic acid. The ester hydrolysis of arecoline to generate arecaidine was also detected. Further metabolism of arecaidine occurred by either *N*-oxidation to arecaidine *N*-oxide or by reduction of the double bond to yield *N*-methyl nipecotic acid, followed by conjugation with glycine. Arecaidine also formed conjugates with glycine, glycerol, and mercapturic acid [18,19]. No reports were found regarding the biotransformation of arecoline using fungi. Herein, the aim of this study was to generate metabolites from arecoline with potential higher cytotoxic activity compared to the precursor molecule. We conducted a fungal bioconversion study of arecoline using 31 strains, and the most efficient ones were selected for large-scale preparation and isolation of the metabolites. The isolated metabolites were screened for cytotoxic effects on normal and cancer cell lines for both lung and blood cells. Moreover, in silico molecular docking of the elucidated structures to the M3-muscarinic receptor model (M3-mT4L, PDB: 4U15) and ADME prediction were conducted to further explain the results. M3-mT4L is a fusion protein of M3-muscarinic receptor and T4 lysozyme to facilitate obtaining a crystal structure of M3 at high resolution [20].

## 2. Results and Discussion

### 2.1. Identification of the Isolated Compounds

**Figure 1:**
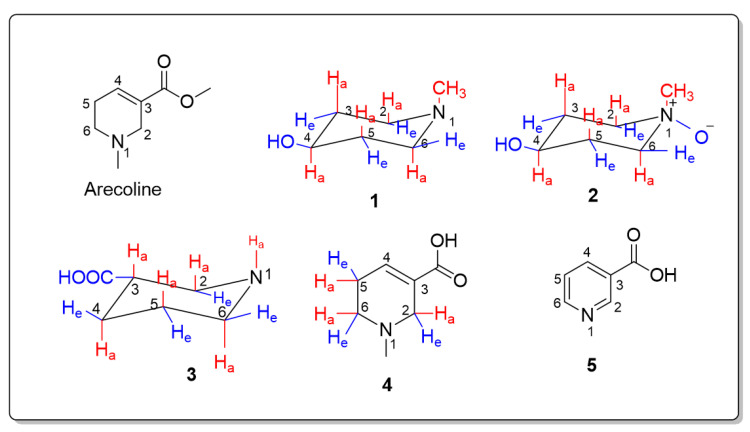
Chemical structures of arecoline and isolated compounds **1**–**5**.

Compound **1**: The ^13^C NMR spectrum (CDCl_3_) showed four signals corresponding to six carbons. Signals at δ_C_ 33.9 and 53.0 represented two magnetically equivalent carbons for each. Compared to arecoline [18,21,22], the signal at δ_C_ 66.0 suggested an oxygenated carbon and was assigned to C-4, while the signal at δ_C_ 45.7 suggested N-CH_3_ (Figure S1). These data suggested a 1,4-disubstituted ring. The presence of magnetically equivalent carbons, the N-CH_3_ signal, and the chemical shifts of the carbons compared to arecoline indicated the reduction of the double bond at the C3-C4 position and oxygenation at the C-4 position. Thus, the structure was deduced as 4-hydroxy-*N*-methylpiperidine. As in 4-hydroxy-*N*-methylpiperidine betaine [23], the hydroxyl group caused the deshielding of C-4, and the nitrogen atom caused the deshielding of C-2 and C-6; thus, the signal at δ_C_ 33.9 was assigned to C-3 and C-5, while the signal at δ_C_ 53.0 was assigned to C-2 and C-6. The ^1^H NMR data (CDCl_3_) for compound **1** (Figure S2) revealed the presence of axial and equatorial protons at positions C-2, C-6, C-3, and C-5. For six-membered rings containing nitrogen atoms, the axial protons are more shielded than the equatorial protons due to the anisotropy of the lone pair of electrons on the nitrogen atom [23,24,25]. The assignment of these protons was based on the COSY and HSQC data. Thus, the two broad singlet peaks at δ_H_ 1.81 and 2.40, each integrating for two protons, were assigned to the axial and equatorial protons H-2a,6a and H-2e,6e, respectively. A singlet peak at δ_H_ 1.94 was integrated for three protons corresponding to N-CH_3_. Two peaks at δ_H_ 1.27 and 1.52, each integrating for two protons, were assigned for the axial and equatorial protons H-3a,5a (multiplet) and H-3e,5e (broad singlet), respectively. A broad singlet peak at δ_H_ 3.29 integrating for one proton was assigned to the proton at C-4. The ^1^H-^1^H COSY correlation confirmed the proposed assignment of protons. The H-4 proton was correlated to the axial protons at C-3 and C-5. The axial protons at C-3 and C-5 exhibited correlations to the equatorial protons at the same carbons and to the axial protons at C-2 and C-6, respectively. The equatorial protons at C-2 and C-6 exhibited a correlation to the axial protons at the same carbons and a weak correlation to the equatorial protons at C-3 and C-5, respectively (Figure S3). The HSQC spectrum indicated a correlation between H-4 and δ_C_ 66.0 (Figure S4). The HMBC spectrum showed correlations of the protons at C-3 to C-5, the protons at C-5 to C-3, the protons at C-2 to C-3 and C-4, and the protons at C-6 to C-4 and C-5 (Figure S5). For the determination of the relative configuration, the NOE spectrum (Figure S6) indicated the correlation between H-4 and H-3e and H-5e, which indicated that H-4 is an axial proton and, thus, the hydroxyl group is equatorial. The IR spectrum showed a characteristic strong, broad band of O-H stretching at 3356 cm^−1^ and a strong band of C-O at 1072 cm^−1^ in the absence of the (C=O) band (Figure S7). These data confirmed the presence of a hydroxyl group. The ESIMS data indicated a deprotonated molecular ion peak [M-H]^−^ at *m*/*z* 113.7 and a pseudo molecular ion peak [M+K-2H]^−^ at *m*/*z* 151.7 (Figure S8). This compound was found optically inactive, which could be related to its symmetric structure. These data confirmed the structure of compound **1** to be 1-methylpiperidin-4-ol (Figure 1) which is considered one of the building blocks for the chemical synthesis of bis(ammonio)alkane-type compounds [26]. This is the first report of the isolation and full characterization of this compound from a biological source.

Compound **2**: The DEPTQ NMR spectrum (D_2_O) yielded four signals corresponding to six carbons. Signals at δ_C_ 27.5 and 61.6 represented two magnetically equivalent carbons for each. The signal at δ_C_ 61.2 for a methine carbon suggested oxygenation and was assigned to C-4, while the signal at δ_C_ 58.1 proposed N-CH_3_ (Figure S9). Because of the nitrogen atom and the hydroxyl group, as discussed in compound **1**, the signal at δ_C_ 27.5 for a methylene carbon was assigned to C-3 and C-5, while the signal at δ_C_ 61.6 for a methylene carbon was assigned to C-2 and C-6. These data suggested a 1,4-disubstituted ring which was confirmed by the ^1^H NMR spectrum. The ^1^H NMR data (D_2_O) of compound **2** (Figure S10) revealed axial and equatorial protons at the positions C-2, C-6, C-3, and C-5, which were assigned with the aid of COSY and HSQC analysis as in compound **1**. It showed two multiplet signals at δ_H_ 3.03 and 3.43, each integrating for two protons, assigned to the axial and equatorial protons H-2a,6a, and H-2e,6e, respectively. A singlet peak at δ_H_ 3.06 was integrated for three protons corresponding to N-CH_3_. The multiplet signals at δ_H_ 1.59 and 2.06, each integrating for two protons, were assigned to the axial and equatorial protons H-3a,5a and H-3e,5e, respectively. A multiplet signal at δ_H_ 3.95 was integrated for one proton corresponding to the proton at C-4. The ^1^H-^1^H COSY correlation confirmed the proposed assignment of protons. The H-4 proton was correlated to the axial protons at C-3 and C-5, which indicated that the hydroxyl group is equatorial. Additionally, the axial protons at C-3 and C-5 showed a strong correlation to the equatorial geminal protons at the same carbons and to the axial protons at C-2 and C- 6, respectively. Weak correlations were observed for the equatorial protons at C-3 and C-5 to H-4 and to the axial protons at C-2 and C-6. The equatorial protons at C-2 and C-6 exhibited a strong correlation to the axial geminal protons at the same carbons, a strong correlation to the equatorial protons at C-3 and C-5, and a weak correlation to the axial protons at C-3 and C-5 (Figure S11). The DEPTQ and ^1^H NMR correlation spectra indicated a correlation for H-4 and δ_C_ 61.2 (Figure S12). These data are consistent with those of compound 1. However, C-2, C-6, and N-CH_3_ are more deshielded, indicating an electronegative substitution affecting these sites. The HMBC spectrum (Figure S13) revealed a correlation of the axial protons at C-2 and C-6 to the *N*-methyl carbon and the equatorial protons at C-2 and C-6 to C-6 and C-2, respectively. The HMBC analysis revealed a correlation of the axial protons at C-3 and C-5 to C-5 and C-3, respectively. The protons at C-3 and C-5 exhibited a correlation to C-4. The protons at C-2 were correlated to C-3, C-4, and C-6, and the protons at C-6 were correlated to C-4 and C-5. For the determination of the relative configuration, as in **1**, the NOE spectrum (Figure S14) indicated the correlation between H-4 and H-3e and H-5e, which indicated that H-4 is axial and, thus, the hydroxyl group is equatorial. The IR spectrum exhibited a characteristic strong, broad band of O-H stretching at 3431 cm^−1^ and a band of N-O at 1454 cm^−1^ (Figure S15). These data suggested an *N*-oxide substitution. HRESIMS of compound **2** (Figure S16) exhibited pseudo molecular ions of [M + H]^+^ at *m*/*z* 132.10205 (calculated 132.10245) and [2M+H]^+^ at *m*/*z* 263.19652 (calculated 263.19708). The HRESIMS data, in conjunction with ^13^C NMR data, suggested a molecular formula of C_6_H_13_NO_2_ for compound **2**. Elemental analysis represented C, 55.12%; H, 10.07%; N, 10.79%; and O, 24.02% (calculated C, 54.94%; H, 9.99%; N, 10.68%; and O, 24.39%). This compound was found optically inactive, which could be related to its symmetric structure. The comparison of the data to those of arecoline [18,21,22] and compound **1** confirmed the structure of compound **2** to be 4-hydroxy-1-methylpiperidine 1-oxide (Figure 1). To our knowledge, this is the first report on this compound, which could be of importance for chemical synthesis.

Compound **3**: Examination of the DEPTQ spectra (D_2_O) of compound **3** (Figure S17) showed six signals corresponding to six different carbon atoms. The spectrum exhibited a carbonyl signal at δ_C_ 180.0, a signal for methine carbon at δ_C_ 40.8, and four signals for methylene carbons at δ_C_ 45.6, 25.6, 21.0, and 43.8. Compared to arecoline [18,21,22], these data indicated double bond reduction, ester hydrolysis, and *N*-demethylation. Thus, a piperidine ring with a carboxyl group at C-3 was suggested for compound **3**. Accordingly, C-2 was the most deshielded, as affected by the nitrogen atom and the carbonyl group at C-3, followed by C-6, C-4, and C-5. Based on this, the signals at δ_C_ 45.6, 43.8, 25.6, and 21.0 were assigned to C-2, C-6, C-4, and C-5, respectively. The ^1^H NMR data of compound **3** (D_2_O, 400 MHz) (Figure S18) demonstrated different peaks integrating for nine protons. One multiplet at δ_H_ 2.35, integrating for one proton, was assigned to a methine proton at C-3. Multiplet peaks at δ_H_ 1.41 and 1.77, each integrating for one proton, were assigned to H-4a and H-4e, respectively. In addition, multiplet peaks at δ_H_ 1.48 and 1.63 were ascribed to H-5a and H-5e, respectively. Multiplet peaks at δ_H_ 2.78 and 2.86, each integrating for one proton, were assigned to the axial protons H-6a and H-2a, respectively. Muliplet peaks at δ_H_ 2.99 and 3.10, each integrating for one proton, were assigned to the equatorial protons H-6e and H-2e, respectively. The ^1^H-^1^H COSY (400 MHz) spectrum confirmed the assignment of the protons (Figure S19). The proton H-5a showed correlations to H-5e, H-6a, and H-4a. The proton H-4a was correlated to H-4e, H-5a, and H-3. The correlations of H-3 to H-2a and H-4a were also indicated. The protons H-2e and H-6e showed correlations to H-2a and H-6a, respectively. The DEPTQ and ^1^H NMR correlation spectrum confirmed the assignment of the protons (Figure S20). The protons H-5a,e were correlated to δ_C_ 21.0, while the protons H-4a,e were correlated to δ_C_ 25.6. The proton H-3 was correlated to δ_C_ 40.8, and the protons H-2a,e were correlated to δ_C_ 45.7. The protons H-6a,e were correlated to δ_C_ 43.9. The analysis of the HMBC spectrum (Figure S21) revealed the correlation of H-3 to C-2, C-4, C-5, and the carbonyl. The protons at C-4 were correlated to C-2, C-3, C-5, and the carbonyl. The protons at C-5 were correlated to C-3, C-4, and C-6. The protons at C-2 were correlated to C-3, C-4, C-6, and the carbonyl. Finally, the protons at C-6 were correlated to C-2, C-4, and C-5. For the determination of the relative configuration, the NOE spectrum (Figure S22) indicated the correlation between H-3 and H-4e, which indicated that H-3 is axial and, thus, COOH group is equatorial. The IR spectrum (Figure S23) showed a broad O-H absorption band at 3421 cm^−1^, a characteristic C=O peak at 1631 cm^−1^, and a strong band of an acyl (C-O) stretch at 1390 cm^−1^. The ESIMS spectrum of compound **3** (Figure S24) revealed the presence of a deprotonated molecular ion peak [M-H]^−^ at *m*/*z* 128.1. The specific rotation ${\left[\alpha \right]}_{D}^{20}$ of **3** is +5 (C 1, water). The results of compound **3** were in good concordance with the data in the literature for nipecotic acid (Figure 1) [27,28].

Compound **4**: The DEPTQ spectrum (D_2_O) of compound **4** (Figure S25) showed seven signals. A signal for a carbonyl group at δ_C_ 167.1, with the disappearance of the ester signal compared to arecoline, suggested an acid. The other signals matched those of arecoline. Thus, the arecaidine structure was anticipated for compound **4**. The carbons of the double bond at C-3 and C-4 resonated at δ_C_ 123.4 and 138.2. Taking into consideration the effect of the nitrogen atom, the chemical shifts at δ_C_ 50.5, 22.6, and 49.4 were assigned for C-2, C-5, and C-6, respectively. The N-CH_3_ was represented by a signal at δ_C_ 42.3 (Figure S25). The ^1^H NMR spectrum (Figure S26) indicated a singlet signal integrating for 3 protons at δ_H_ 2.88 for N-CH_3_. A broad singlet resonating at δ_H_ 7.09 was assigned to H-4. As in arecoline, the deshielding effect of the nitrogen atom caused a downfield shift and magnetic inequivalence of the protons at C-2 and C-6 compared to C-5. The protons of C-2 were more deshielded than those of C-6 due to the additional effect of the double bond and the carboxyl group at C-3. Protons of C-2 were found magnetically inequivalent and represented by signals at δ_H_ 3.64 (doublet of doublets, *J* = 16.2 and 2.4 Hz) for H-2a and at δ_H_ 4.06 (doublet, *J* = 16.2 Hz) for H-2e. The same goes for the protons at C-6; they appeared as two multiplets at δ_H_ 3.10 and 3.46, each integrating for one proton. Accordingly, a multiplet integrating for 2 protons at δ_H_ 2.57 was ascribed to C-5 protons. The peak for the methyl ester of arecoline at δ_H_ 5.44 was absent. The ^1^H-^1^H COSY analysis (Figure S27) indicated the correlation between the axial and equatorial protons at C-2. The axial proton at C-6 correlated to the equatorial proton at the same carbon and to the protons at C-5. The proton H-4 correlated to the protons at C-5. The DEPTQ and ^1^H NMR correlation spectrum (Figure S28) showed the correlation between H-4 and δ_C_ 138.2, protons of C-5 and δ_C_ 22.6, *N*-methyl and δ_C_ 42.3, protons of C-2 and δ_C_ 50.5 and lastly protons of C-6 and δ_C_ 49.4. The HMBC analysis confirmed the protons assignment (Figure S29). The axial and equatorial protons at C-2 showed correlations to C-3, C-4, and methyl carbon, while only the equatorial proton at C-2 exhibited correlations to the carbonyl carbon and C-6. The proton H-4 correlated to the carbonyl carbon, C-2, C-5, and C-6. Protons at C-5 showed correlations to C-3 and C-4. The methyl carbon correlated to C-2, C-3, C-5, and C-6. The axial and equatorial protons at C-6 correlated to C-2, C-4, C-5, and the methyl carbon. The IR spectrum (Figure S30) showed a broad O-H absorption band at 3431 cm^−1^, a characteristic C=O peak at 1725 cm^−1^, and a strong acyl C-O peak at 1189 cm^−1^. The ESIMS spectrum of compound **4** (Figure S31) displayed a protonated molecular ion peak [M+H]^+^ at *m*/*z* 142.1. All these data suggested the structure of compound **4** as arecaidine (Figure 1), which was confirmed by matching the published data [29].

Compound **5**: The ^13^C NMR spectrum (D_2_O) showed five carbons with δ_C_ 145.8, 135.5, 142.4, 126.9, and 143.0, which suggested an aromatic structure (Figure S32). A signal at δ_C_ 168.3 alongside the absence of signals for oxygenated carbons indicated an acid group. The protonated ion peak [M + H]^+^ at *m/z* 121.8 (Figure S33) implied the presence of a nitrogen atom. These data suggested a pyridine nucleus substituted with a carboxyl group. The ^1^H NMR spectrum (D_2_O) of compound **5** (Figure S34) confirmed the aromatic nature. The multiplicities of the detected protons as a singlet, two doublets, and a doublet of doublets implied substitution at C-3. A singlet at δ_H_ 9.01, integrating for one proton, was assigned to H-2 due to the deshielding effect of the nitrogen atom of the pyridine nucleus and the carboxyl group at C-3. Two doublet peaks at δ_H_ 8.72 (*J* = 6.0 Hz) and δ_H_ 8.81 (*J* = 8.0 Hz) integrated for one proton for each and were assigned to H-4 and H-6, respectively. This assignment was proposed based on the deshielding impact of the nitrogen atom on C-6. A doublet of doublets peak at δ_H_ 7.99 (*J*= 8.0 and 6.0 Hz) integrated for one proton corresponding to H-5. The ^1^H-^1^H COSY spectrum (Figure S35) demonstrated a correlation between H-5 to C-4 and C-6. The proton H-4 correlated to C-5 and the proton H-6 correlated to C-5. By HSQC analysis (Figure S36), the carbon atoms were assigned as C-2 at δ_C_ 142.4, C-3 at δ_C_ 135.5, C-4 at δ_C_ 143.0, C-5 at δ_C_ 126.9, C-6 at δ_C_ 145.8 and carbonyl group at δ_C_ 168.3. The HMBC analysis (Figure S37) indicated correlations for H-5 to C-3 and C-4. The proton H-4 correlated to C-2, C-6, and C-5, while H-6 correlated to C-2 and C-4. Additionally, the proton H-2 showed correlations to C-4 and C-6. The IR spectrum (Figure S38) showed a broad O-H absorption band at 3449 cm^−1^, a band for =C-H stretch at 3076 cm^−1^, a characteristic C=O band at 1701 cm^−1^, a band for C=C stretching at 1639 cm^−1^ and a strong band for an acyl C-O group at 1298 cm^−1^. These spectral data were consistent with those published for nicotinic acid (Figure 1) [30].

### 2.2. Biological Activity

#### 2.2.1. In Vitro Cytotoxic Activity

##### In Vitro Cytotoxic Activity Using Cancer Cell Lines

An assay using 3-(4, 5-dimethylthiazol-2-yl)-2,5-diphenyltetrazolium bromide (MTT) was performed to evaluate the cytotoxic activity of compounds **1**–**5** in vitro using the non-small-cell lung cancer A549 and leukemia K562 cell lines. The results are shown in Table 1. Staurosporine and doxorubicin were used as positive controls. For the non-small-cell lung A549 cell line, arecoline hydrobromide, staurosporine, and doxorubicin showed IC_50_ values of 11.73 ± 0.71 µM, 10.47 ± 0.64 µM, and 5.05 ± 0.13 µM, respectively, while compounds **1**, **3** and **5** exhibited IC_50_ values of 3.08 ± 0.19 µM, 7.33 ± 0.45 µM, and 3.29 ± 0.20 µM, respectively. In contrast, compounds **4** and **2** exhibited IC_50_ of 90.90 ± 0.59 µM and >100 µM, respectively.

For the leukemia K562 cell line, the IC_50_ values of arecoline hydrobromide, staurosporine, and doxorubicin were 15.3 ± 1.08 µM, 5.07 ± 0.36 µM, and 6.94 ± 0.21 µM, respectively, while the IC_50_ values of compounds **1**, **3** and **5** were 1.56 ± 0.11 µM, 3.33 ± 0.24 µM, and 2.15 ± 0.15 µM, respectively. However, compounds **4** and **2** presented IC_50_ of 67.3 ± 0.53 µM and >100 µM, respectively.

These results revealed that compounds **1**, **3**, and **5** showed very strong cytotoxicity higher than arecoline hydrobromide, staurosporine, and doxorubicin against non-small-cell lung cancer A549 and leukemia cancer K562 cell line. Compounds **2** and **4** possessed low and moderate cytotoxicity, respectively (Table 1).

To our knowledge, this is the first report of the in vitro cytotoxic activity of compounds **1**, **3**, and **5** using the non-small-cell lung cancer A49 and for compounds **1** and **3** against the leukemia cancer K562 cell line. Ida et al. studied the cytotoxic effect of nicotinic acid and some related derivatives against the leukemia K562 cell line using an MTT assay [31]. The authors reported that nicotinic acid inhibited leukemia cancer K562 cell proliferation and induced differentiation to erythrocytes.

**Figure 2:**
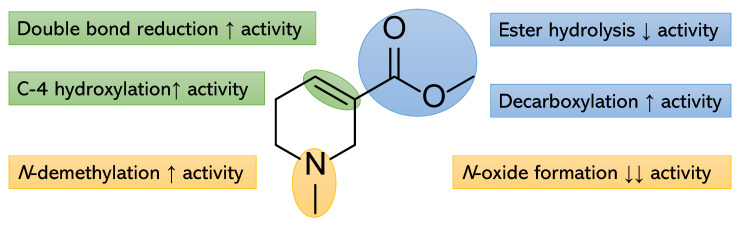
Summary of potential hotspots for structural modification of arecoline metabolites.

According to these results, we can conclude that the structural changes to arecoline resulted in differences in the IC_50_ against the tested cell lines. The ester hydrolysis, as in compound **4**, resulted in a decrease in the activity. However, ester hydrolysis accompanied by *N*-demethylation and double bond reduction, as in compound **3**, resulted in an increase in activity. Double bond reduction, decarboxylation, and *N*-demethylation together with hydroxylation at C-4, as in compound **1**, resulted in an increase in activity. However, the same changes alongside *N*-oxide formation, as in compound **2**, resulted in a dramatic decrease in activity (Figure 2).

##### In Vitro Cytotoxic Activity Using Normal Cell Lines

As compounds **1**, **3**, and **5** presented the highest cytotoxic effect amongst the tested compounds, they were further examined against normal lung cell WI38 and normal blood cell PCS-800-016 using MTT assay (Table 2) to determine the selectivity index. The selectivity index identifies the selectivity of the tested compounds toward the cancer cell line and is calculated by dividing the IC_50_ against the normal cell line by the IC_50_ against the cancer cells.

Compounds **1**, **3**, **5**, and staurosporine possessed selectivity indices of 5.93, 3.64, 5.00, and 1.57, respectively, toward non-small-cell lung cancer A549. It was also found that compounds **1**, **3**, **5**, and staurosporine exerted selectivity indices of 6.32, 11.37, 9.35, and 2.56, respectively, toward leukemia cancer cells K562. The value of the selectivity index of compounds **1**, **3**, and **5** indicated high selectivity toward the tested cell lines [32,33,34,35], as the higher the magnitude of the selectivity index (more than 3) of a compound is, the greater its selectivity [36].

#### 2.2.2. Flow Cytometry Analysis of DNA Content for Cell Cycle and Apoptosis

##### Cell Cycle Arrest and Apoptosis Induced by Compound **1** Using Non-Small-Cell Lung Cancer A549

**Figure 3:**
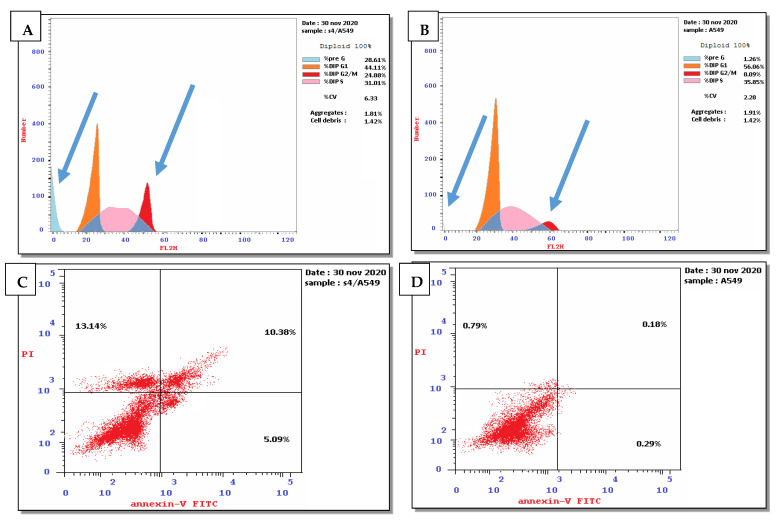
(**A**) Effect of compound **1** on the cell cycle distribution of non-small-cell lung cancer A549 cell line. (**B**) Untreated lung cancer cell as a negative control. (**C**) Apoptosis induced by compound **1** using non-small-cell lung cancer A549 cell line. (**D**) Apoptosis in untreated cells as negative control. The four quadrants identified as: lower left: viable; lower right: early apoptotic; upper right: late apoptotic; upper left: necrotic.

The results of the selectivity index indicated that compound **1** displayed the highest selectivity toward non-small-cell lung cancer A549 cells; thus, the effect of compound **1** on the cell cycle distribution and apoptosis was explored compared to untreated cancer cells as a negative control. The results of the DNA flow cytometry experiment using non-small-cell lung cancer A549 cells showed that compound **1** affected the cell cycle distribution and induced apoptosis. This was demonstrated by an increasing percentage of cells in the sub-G1 phase and G2/M phase (28.61% and 24.88%, respectively) compared to the untreated cells (1.26% and 8.09%, respectively). Moreover, compound **1** also exhibited a decline in the percentage of cells in the S-phase and G0/G1 phase by 31.01% and 44.11%, respectively, compared to the cell cycle distribution in the untreated cells (35.85% and 56.06%, respectively) (Table S1 and Figure 3A,B). Furthermore, compound **1** induced early and late apoptosis and necrosis (5.09%, 10.38%, and 13.14%, respectively) compared to the untreated cells (0.29%, 0.18%, and 0.79, respectively). These results indicated that the tested compound induced total cell death of 28.61% compared to the untreated cells as a negative control, 1.26% (Table S2 and Figure 3C,D).

These results also indicate that compound **1** mediated cell cycle arrest in the G2/M phase. Moreover, compound **1** increased early apoptosis, late apoptosis, and necrosis by 17.55-, 57.67-, and 16.63-fold, respectively, compared to the untreated lung cancer cells. Moreover, it increased total cell death levels by 22.71-fold compared to the negative control.

##### Cell Cycle Arrest and Apoptosis Induced by Compounds **3** and **5** Using Leukemia Cancer Cell K562

**Figure 4:**
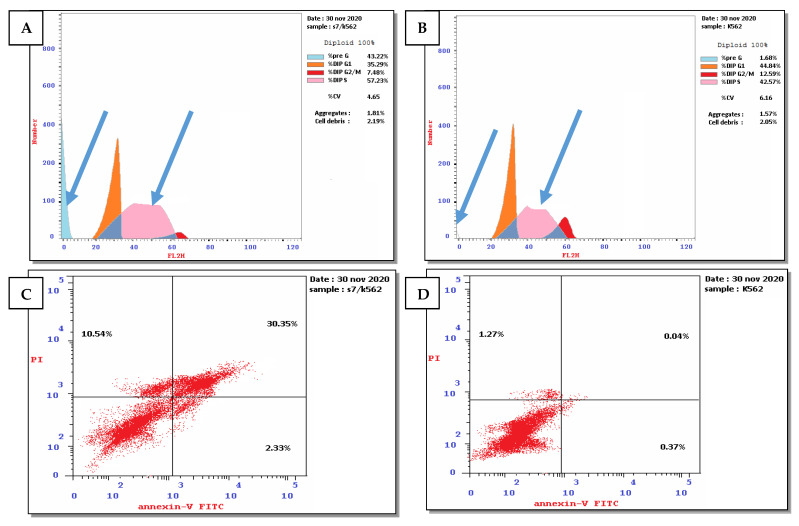
(**A**) Effect of compound **3** on the cell cycle distribution of leukemia cell line K562. (**B**) Untreated leukemia cell was used as negative control. (**C**) Apoptosis induced by compound **3** using leukemia cell line K562. (**D**) Apoptosis in untreated cells as a negative control. The four quadrants identified as: lower left: viable cells; lower right: early apoptotic cells; upper right: late apoptotic cells; upper left: necrotic cells.

**Figure 5:**
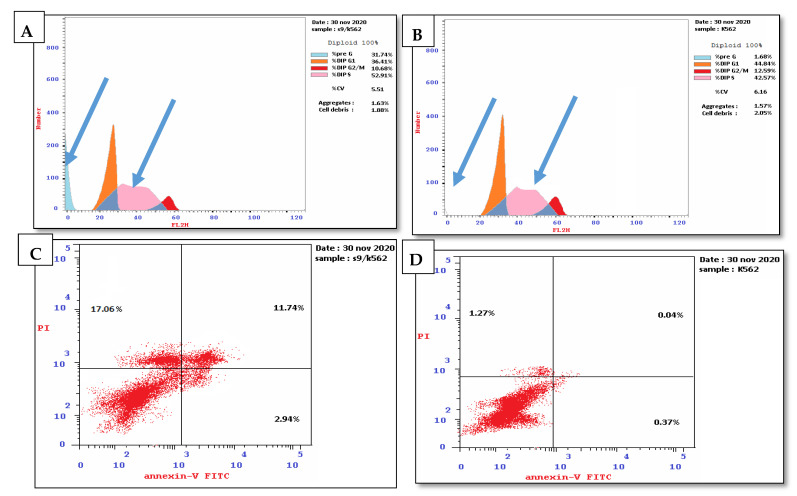
(**A**) Effect of compound **5** on the cell cycle of leukemia cell line K562. (**B**) Untreated cells were used as negative control. (**C**) Apoptosis induced by compound **5** using leukemia cell line. (**D**) Apoptosis of untreated cells as negative control. The four quadrants identified as: lower left: viable cells; lower right: early apoptotic cells; upper right: late apoptotic cells; upper left: necrotic cells.

The results of the selectivity index indicated that compounds **3** and **5** displayed the highest selectivity toward K562 leukemia cancer cells; therefore, the effect of compounds **3** and **5** on the cell cycle distribution and induction of apoptosis was investigated compared to the untreated cancer cells as a negative control (Tables S1 and S2) and (Figure 4 and Figure 5).

For compound **3**, the results demonstrated an increase in the percentage of cells in the sub-G1 phase and S-phase (43.22% and 57.23%, respectively) compared to the untreated cells (1.68% and 42.57%, respectively). It also showed a decline in the percentage of cells in G2/M and G0/G1 phase (7.48% and 35.29%, respectively) compared to the untreated cells (12.59% and 44.84%, respectively). These results indicated that compound **3** induced cell cycle arrest at the S phase (Table S1 and Figure 4A,B). Moreover, compound **3** induced early apoptosis, late apoptosis, and necrosis (2.33%, 30.35%, and 10.54, respectively) compared to the untreated cells (0.37%, 0.04%, and 1.27, respectively). These results indicated that compound **3** induced total cell death by 43.22%, which is higher than the negative control (1.68%). Furthermore, the results revealed that compound **3** enhanced early apoptosis, late apoptosis, and necrosis by 6.30, 758.75, and 8.30-fold higher than the negative control (Table S2 and Figure 4C,D).

On the other hand, compound **5** increased the percentage of the cell population in sub-G1 and S-phase (31.74% and 52.91%, respectively) compared to the untreated cells (1.68% and 42.57%, respectively). In addition, compound **5** exhibited a decline in the percentage of cells in the G2/M and G0/G1 phase (10.68% and 36.41%, respectively) compared to the untreated cells (12.59 % and 44.84 %, respectively) (Table S1 and Figure 5A,B).

Additionally, the results revealed that compound **5** induced early, late apoptosis and necrosis (2.94%, 11.74%, and 17.06, respectively) compared to the untreated cells (0.37%, 0.04%, and 1.27%, respectively). Thus, compound **5** increased total cell death by 31.74% compared to the untreated cells (1.68%) (Table S2 and Figure 5C,D). The results implied that compound **5** arrested the cell cycle in the S phase and induced early, late apoptosis and necrosis by 7.95-, 293.50-, and 13.43-fold, respectively, higher than the untreated cell. These results are in agreement with the previously reported data of compound **5** on leukemia HL-60 [37,38].

### 2.3. In Silico Molecular Docking

As the arecoline cytotoxic effect can be mediated through binding to muscarinic receptors, the cytotoxic mechanism of the isolated compounds was investigated using in silico docking simulation to muscarinic receptors. In the present study, the docking simulation of arecoline and compounds **1**–**5** to the M3-mT4L protein, which is an M3-mAChR bound to tiotropium (PDB ID: 4U15), was carried out using Molegro Virtual Docker (MVD) software.

**Figure 6:**
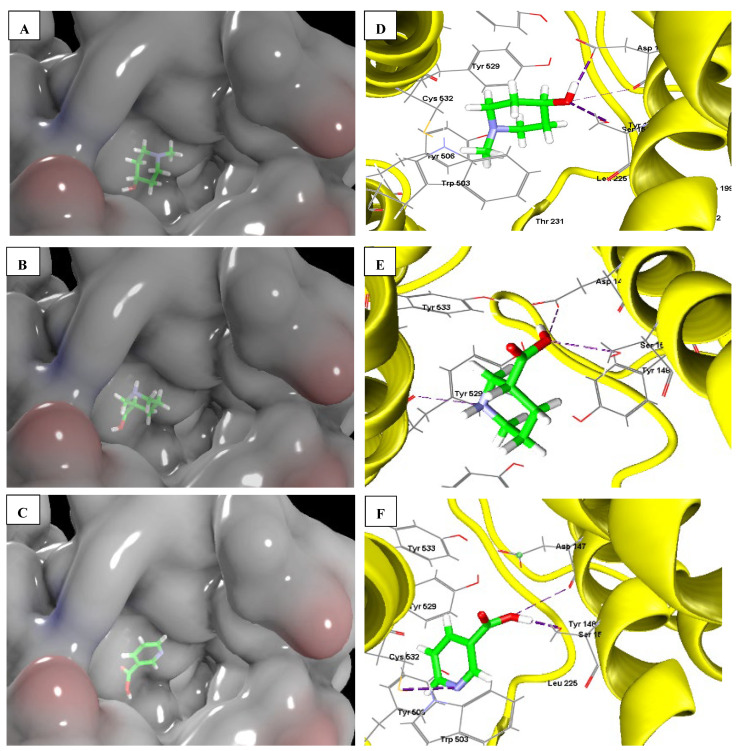
The binding mode of compounds **1, 3,** and **5**. Electrostatic surface representation of ligand binding pocket of compounds **1** (**A**), **3** (**B**), and **5** (**C**). Amino acid residues involved in ligand interaction and hydrogen bonds are shown as blue dashed lines, **1** (**D**), **3** (**E**), and **5** (**F**).

The results revealed that compounds **1**–**5** possessed good binding energies and favorable protein-ligand interactions with M3-mT4L protein, as shown in Table S3 and Figure 6 and Figure S39 [20]. According to the MolDock binding score, arecoline and compounds **1**–**5** possessed good binding energies of −81.15, −62.19, −73.43, −66.20, −75.37, and −65.18 kcal/mol, respectively. The binding modes for compounds **1**–**5** were similar to those of arecoline. The amino acids involved in hydrogen bonding interactions of arecoline and compounds **1**–**5** with the M3-mT4L protein are presented in Table S4. Hydrogen bonding interaction with the active site amino acid Ser151 was found in the docking poses of arecoline and compounds **1**–**5**. In addition, arecoline tightly interacted via hydrogen bonding with Cys532, compound **1** interacted with Asp147, and compound **3** interacted with Tyr529 and Asp147. On the other hand, compounds **2**, **4**, and **5** tightly interacted with Cys532 and Asp147, as presented in Figure 6 and Figure S39.

This result is an indicator that compounds **1**–**5** are efficient binders to the M3-mAChR as they fit into the binding site and interact with the active site residues that are crucial for biological activity.

Although compounds **2**, **4**, and **5** showed the same interactions with the active site residues, they exhibited different IC_50_ values, as discussed above. Out of these compounds, only compound **5** exhibited a profound activity. Thus, we can conclude that there are other factors affecting the measured activity and cannot solely be explained by the docking results. Pharmacokinetic ADME properties were calculated for more explanation.

### 2.4. Prediction of Drug-Likeness and Pharmacokinetics ADME

Based on online SwissADME prediction, arecoline and compounds **1**–**5** were predicted to be drug-like with a high probability of good oral bioavailability according to Lipinski, Veber, and Egan rules with zero violation [39,40,41]. Only compound **5** was predicted to permeate the blood–brain barrier (Table S5 and Figure S40). Arecoline and metabolites **1**–**5** have a molecular weight of <500 Da, hydrogen bond donor <5, hydrogen bond acceptor <10, rotatable bonds fewer than 10, TPSA less than 132 Å2, and logP in a range between −1 and 6. Therefore, arecoline and metabolites **1**–**5** are predicted to be a drug like with a high probability of good oral bioavailability according to Lipinski, Veber, and Egan rules with zero violation from these rules. The results of the BOILED-Egg plot revealed that all metabolites could be passively absorbed through the gastrointestinal tract, but only metabolite 5 could permeate blood–the brain barrier and not be affected by P-glycoprotein mediated extrusion from the central nervous system.

In addition, using the PreADMET web-based tool, all compounds were predicted to be well absorbed and distributed except for compound **2**, which exhibited low permeability using Caco-2 cells as a model (Table S6). An additional prediction model, PerMM, was applied to predict black liquid membrane (BLM) and parallel artificial membrane permeability assay (PAMPA), as well as Caco-2 cell permeability [42,43]. The results are presented in Table S7 and Figure S41. Metabolite **2** was predicted to have low permeability across all tested membrane types, as indicated by the lowest negative log permeability coefficient value across arecoline and its metabolites. This result might help to explain the low in vitro cytotoxic effect of compound **2** despite the high docking score. Additionally, compound **4** was predicted to have high plasma protein binding (91.95%) compared to the other compounds. In vivo studies are required for a better explanation.

## 3. Experimental Procedures

### 3.1. General Procedures

All solvents used for extraction, isolation, and NMR analysis were purchased from either E. Merck (Darmstadt, Germany) or Sigma Aldrich Co. (St. Louis, MO, USA). Arecoline hydrobromide was purchased from Santa Cruz Biotech., Inc. (Dallas, TX, USA). A Bruker Avance 400 spectrophotometer (Karlsruh, Germany), using CDCl_3_ or D_2_O as a solvent and tetramethylsilane (TMS) as an internal standard, was used for NMR analysis at 400 MHz for ^1^H NMR and 100 MHz for ^13^C NMR. HRESIMS analysis was performed using LC/Q-TOF, 6530 (Agilent Technologies, Santa Clara, CA, USA). ESIMS analysis was achieved by Advion compact mass spectrometer (CMS, New York, NY, USA) supported with ESI ion source. A FLASH 2000 CHNS/O analyzer (Thermo Scientific, Loughborough, UK) was used for elemental analysis. Polax-21 (Atago Co., Ltd., Tokyo, Japan) was used for the measurement of the optical activity. The purity of the compounds was detected on precoated silica gel G60 F254 plates (0.25 mm layer, E. Merck, Darmstadt, Germany) using four different solvent systems (SS1–SS4). These solvent systems consist of ethanol: petroleum ether:2 M NH_3_ (6:1:1 *v*/*v*) for SS-1, ethanol:acetic acid:water (1:1:8 *v*/*v*) for SS-2, ethyl acetate: methanol (7:3 *v*/*v*) for SS-3, and dichloromethane:methanol (5.5:4.5 *v*/*v*) for SS-4. Visualization was achieved by Dragendorff’s spray reagent.

### 3.2. Microorganisms, Culture Conditions, and Fermentation Procedures

#### 3.2.1. Microorganisms

About 31 strains of fungi were tested in the initial screening of arecoline hydrobromide. The tested strains (Table S8) were obtained from American Type Culture Collection (ATCC), Northern Regional Research Laboratory (NRRL), Assiut University Mycological Center (AUMC), and the Department of Microbiology, College of Pharmacy, King Saud University. The strains were maintained on potato dextrose agar (DIFCO, Detroit, USA) or Sabouraud dextrose agar (Becton Dickonson and Co., Cockeysville, MD, USA) slants at 4 °C.

#### 3.2.2. Preliminary Screening Procedures

A standard two-stage fermentation technique was followed. The fermentation process and the culture conditions were followed as reported in the previously published work [44,45,46]. Stage I cultures were started by inoculating two-week-old slants of the tested organisms (Table S8) in a sterilized culture medium (50 mL in 250 mL flasks), then they were incubated for three days in a gyratory shaker (Melrose Park, IL, USA) operating at 150 rpm at 27 °C. Stage II cultures were achieved by inoculating stage I culture (5 mL) to each 250 mL flask containing 50 mL of fresh liquid medium. Both substrate and organism-free controls were handled in the same way. Cultures were permitted to grow for 24 h before the addition of arecoline aqueous solution (10 mg/0.25 mL). Samples (5 mL) were withdrawn from each culture on days 3, 7, 10, and 14. Each sample was successively extracted with dichloromethane, ethyl acetate, and *n*-butanol after alkalization by conc. ammonia (pH 8). Each extract was screened on TLC to determine the organisms that were able to convert arecoline hydrobromide, the best incubation period, and the most suitable organic solvent for the extraction of metabolites.

#### 3.2.3. Large Scale Fermentation

*Cunninghamella blakesleeana* NRRL 1369 (family Cunninghamellaceae) and *Aspergillus niger* ATCC 10549 (family Trichocomaceae) induced conversion of arecoline hydrobromide and were selected for large-scale culturing.

For conversion using *C*. *blakesleeana* NRRL 1369, arecoline hydrobromide (1200 mg) was dissolved in sterile water (30 mL) and then equally divided among 30 flasks (each 1 L in size) containing 200 mL of stage II culture medium and incubated at 27 °C on a gyratory shaker operating at 150 rpm. After seven days of fermentation, the experiment was terminated. The fermentation broth, after filtration of the cells, was extracted successively with ethyl acetate and *n*-butanol. The extracts were evaporated to dryness to obtain brown residues for the ethyl acetate (1300 mg) and *n*-butanol (2000 mg) fractions. The obtained residues were examined on silica gel G_60_ precoated TLC plates.

For *A*. *niger*, arecoline hydrobromide (480 mg) was dissolved in sterile water (12 mL), then equally divided among 12 flasks (each 1 L in size) containing 200 mL of stage II culture medium and incubated at 27 °C on a gyratory shaker operating at 150 rpm. After ten days of fermentation, the experiment was terminated. After filtration of the cells, the fermentation broth was extracted using ethyl acetate. The extract was evaporated to dryness under reduced pressure to give a dark brown residue (530 mg). The ethyl acetate residue was examined on silica gel G_60_ precoated TLC plates.

### 3.3. Isolation of Compounds

#### 3.3.1. Isolation of Compounds from Arecoline Hydrobromide Transformation by *C. blakesleeana* NRRL 1369

##### Isolation of Compounds **1** and **2** from Ethyl Acetate Extract

The ethyl acetate residue (1300 mg) was dissolved in methanol and loaded on a silica gel (Sigma Aldrich Co., St. Louis, MO, USA) column (52 g, 54 × 1.6 cm) packed using the dry method. The isocratic elution method was adopted using ethyl acetate:acetone (7.5:2.5). Collected fractions (130, 10 mL each) were examined on precoated silica gel G_60_ TLC plates using solvent system SS-1. Similar fractions were collected together and evaporated to dryness.

Fractions (15–60) were pooled together and evaporated to dryness to give a yellow residue (100 mg). Further purification was carried out via isocratic elution chromatography on a silica gel column (3 g, 16 × 0.5 cm) using ethyl acetate:methanol (9.5:0.5) as a mobile phase. Collected subfractions (85, 1 mL each) were examined on silica gel G_60_ precoated TLC plates using the solvent system SS-2, and similar subfractions were pooled together. Subfractions (47–54) gave compound **1** as an oily substance (23 mg, 1.9% yield). Subfractions (76–82) gave a faint yellow residue (32 mg) and were subjected to further purification using Sephadex LH20 purchased from Sigma Aldrich Co., St. Louis, MO, USA (10 g, 25 × 1 cm), which yielded compound **2** as a white amorphous powder (10 mg, 0.83% yield).

##### Isolation of Compounds **3** and **4** from *n*-Butanol Extract

*n*-Butanol extract (2000 mg) was suspended in deionized water and applied to a Diaion HP-20 column (80 g, 60 × 3 cm). The column was initially eluted using deionized water to remove free sugars and salts, followed by 100% methanol (HPLC grade). Methanol fraction was dried under reduced pressure to give a brown residue (1650 mg). Part of the residue (450 mg) was chromatographed using a silica gel column (18 g, 38 × 1 cm). The isocratic elution method was adopted using dichloromethane:methanol (8.5:1.5), and the collected fractions (160, 1 mL each) were examined on precoated silica gel G_60_ TLC plates using the solvent system SS-2. Similar fractions were pooled together and evaporated to dryness.

Fractions (66–78) gave a faint yellow residue (25 mg) which was subjected to further purification using Sephadex LH20 (10 g, 25 × 1 cm). Subfractions (15–27) yielded compound **3** as white crystals (15 mg, 1.25% yield).

Fractions (120–128) gave a yellow residue (35 mg) which was dissolved in a mixture of ethanol (HPLC grade):dichloromethane (98:2) to precipitate compound **4**, which was recrystallized from methanol (HPLC) as white crystals (25 mg, 2% yield).

#### 3.3.2. Isolation of Compound **5** from Arecoline Hydrobromide Transformation by *A. niger* ATCC 10549

The ethyl acetate residue was chromatographed using dichloromethane:methanol (8.5:1.5) on a silica gel column (16 g, 35 × 1 cm) packed with the dry method. Collected fractions (98, 5 mL each) were examined on silica gel G_60_ precoated TLC plates using the solvent system SS-4. Similar fractions were pooled together and evaporated to dryness. Fractions (19–28) were collected, evaporated till dryness to give a faint yellow residue (27 mg), and further purified using Sephadex LH20 column (10 g, 25 × 1 cm) eluted with methanol (HPLC grade). Subfractions (25, 0.5 mL each) were collected and examined on silica gel G60 precoated TLC plates using the solvent system SS-4. Subfractions (18–22) were pooled and evaporated to dryness to give compound **5** as white crystals (11 mg, 2.3% yield).

### 3.4. Physical and Spectral Data of Compounds

**1-Methylpiperidin-4-ol (1)**

Yield: 1.9%. Aspect: oily. IR (KBr disc) v_max_ (cm^−1^): 3356, 1072. ^1^H NMR (400 MHz, CDCl_3_) δ (ppm): 1.81 (br s, 2H, H-2a/6a), 2.40 (br s, 2H, H-2e/6e), 1.27 (m, 2H, H-3a/5a), 1.52 (br s, 2H, H-3e/5e), 3.29 (br s, 1H, H-4), 1.94 (s, 3H, N-CH3), 5.07 (br s, 1H, O-H). DEPTQ (100 MHz, CDCl_3_) δ (ppm): 53.0, 33.9, 66.0, 45.7. ESI-MS (*m*/*z*) 113.7 [M-H]^−^, 151.7 [M+K-2H]^−^.

**4-Hydroxy-1-methylpiperidine 1-oxide (2)**

Yield: 0.83%. Aspect: white amorphous powder. IR (KBr disc) v_max_ (cm^−1^): 3431, 1454. ^1^H NMR (400 MHz, D_2_O) δ (ppm): 3.03 (m, 2H, H-2a/6a), 3.43 (m, 2H, H-2e/6e), 1.59 (m, 2H, H-3a/5a), 2.06 (m, 2H, H-3e/5e), 3.95 (m, 1H, H-4), 3.06 (s, 3H, N-CH_3_). DEPTQ (100 MHz, D_2_O) δ (ppm): 61.6, 27.5, 61.2, 58.1. HRESIMS (+) *m*/*z* cald. for [M+H]^+^ C_6_H_13_NO_2_ 132.10245, found 132.10205 and *m*/*z* cald. for [2M+H]^+^ 263.19708, found 263.19652. 

**D-piperidine-3-carboxylic acid, D-nipecotic acid (3)**

Yield: 1.25%. Aspect: white crystals. Mp: 261 °C. ${\left[\alpha \right]}_{D}^{20}$ +5 (C 1, water). IR (KBr disc) v_max_ (cm^−1^): 3421, 1631, 1390. ^1^H NMR (400 MHz, D_2_O) δ (ppm): 2.86 (m, 1H, H-2a), 3.10 (m, 1H, H-2e), 2.35 (m, 1H, H-3), 1.41 (m, 1H, H-4a), 1.77 (m, 1H, H-4e), 1.48 (m, 1H, H-5a), 1.63 (m, 1H, H-5e), 2.78 (m, 1H, H-6a), 2.99 (m, 1H, H-6e). DEPTQ (100 MHz, D_2_O) δ (ppm): 45.6, 40.8, 25.6, 21.0, 43.8, 180.0. ESI-MS (*m*/*z*) 128.1 [M-H]^−^.

**1-Methyl-1,2,5,6-tetrahydropyridine-3-carboxylic acid; arecaidine (4)**

Yield: 2%. Aspect: white crystals. Mp: 132 °C. IR (KBr disc) v_max_ (cm^−1^): 3431, 1725, 1189. ^1^H NMR (400 MHz, D_2_O) δ (ppm): 3.64 (dd, *J* = 16.2 and 2.4 Hz, 1H, 2a); 4.06 (d, *J* = 16.2 Hz, 1H, 2e), 7.09 (br s, 1H, H-4), 2.57 (m, 2H, H-5), 3.10 (m, 1H, 6a), 3.46 (m, 1H, 6e), 2.88 (s, 3H, N-CH_3_). DEPTQ (100 MHz, D_2_O) δ (ppm): 50.5, 123.4, 138.2, 22.6, 49.4, 42.3,167.1. ESI-MS (*m/z*) 142.1 [M+H]^+^.

**Pyridine-3-carboxylic acid, nicotinic acid (5)**

Yield: 2.3%. Aspect: white crystals. Mp: 237 °C. UV (MeOH) λ_max_ (nm): 246. IR (KBr disc) v_max_ (cm^−1^): 3449, 3076, 1701, 1640, 1299. ^1^H NMR (400 MHz, D_2_O) δ (ppm): 9.01 (s, 1H, H-2), 8.72 (d, *J* = 6 Hz, 1H, H-4), 7.99 (dd, *J* = 8.0, 6.0, 1H, H-5), 8.81 (d, *J* = 8.0, 1H, H-6). ^13^C NMR (100 MHz, D_2_O) δ (ppm): 142.4, 135.5, 143.0, 126.9, 145.8, 168.3. ESI-MS (*m/z*) 121.8 [M-H]^−^.

### 3.5. Biological Activity

#### 3.5.1. In Vitro Cytotoxic Activity

Cell lines were obtained from the American Type Culture Collection (ATCC). Cells were cultured using Dulbecco’s Modified Eagle’s Medium (DMEM) (Invitrogen/Life Technologies, Waltham, MA, USA) supplemented with 10% fetal bovine serum (FBS, Hyclone, Logan, UT, USA), 10 µg/mL of insulin (Sigma Aldrich, St. Louis, MO, USA) and 1% mixture of penicillin and streptomycin. After incubation for 48 h with the isolated compounds, the viability of cells was measured using an MTT assay to determine the toxic concentration for each compound using the microplate reader according to the published procedures [47,48,49,50].

#### 3.5.2. Flow Cytometry Analysis of DNA Content for Cell Cycle and Apoptosis

For cell cycle analysis, a propidium iodide flow cytometry kit (ab139418, Abcam, Cambridge, UK) was used. For apoptosis, we used an annexin V apoptosis kit, which is composed of annexin V-FITC (fluorescein isothiocyanate), 1X binding buffer, and propidium iodide. Detection was conducted by measuring DNA content using a flow cytometer [51].

### 3.6. Molecular Docking Studies for the Selected Target

Compounds **1**–**5** were docked to the active site of the M3-mT4L receptor bound to tiotropium (PDB ID: 4U15)] [20]. The crystal structure of M3-mT4L was downloaded from the protein databank. The protein complex was prepared, and the receptor grid was generated with Molegro Virtual Docker (MVD 2019 7.0.0) [52]. The protein structure was imported into the workspace of MVD. Each ligand was imported into the workspace as a structure data (*.sdf) file. The docking search algorithm was set to MolDock Simplex Evolution (SE). The docking results were imported into the workspace, and the pose organizer was used to explore the returned poses for each ligand.

### 3.7. Prediction of Drug-Likeness and Pharmacokinetics ADME

The web-based SwissADME tool (http://www.swissadme.ch/index.php, accessed on 14 October 2021) [53] was used to estimate the drug-likeness and oral bioavailability of arecoline and compounds **1**–**5**. Furthermore, it was applied for the prediction of the tested compounds’ permeability to the blood–brain barrier and the gastrointestinal tract. The results are presented as a BOILED-Egg plot [54]. Additionally, the online PreADMET (https://preadmet.bmdrc.kr/, accessed on 15 May 2022) and PerMM (https://permm.phar.umich.edu/, accessed on 15 May 2022) tools were used for the prediction of membrane permeability of compounds. ADME was predicted for arecoline and compounds **1**–**5** [55].

## 4. Conclusions

Fungal transformation of arecoline generated five compounds, of which **1** and **2** were firstly reported. This bioconversion included ester hydrolysis, double bond hydration, *N*-oxide formation, and ring unsaturation. Compounds **1**, **3**, and **5** exhibited in vitro cytotoxic activity against non-small-cell lung cancer A549 and leukemia cancer K562 cell lines with high selectivity indices, which were further explained by in silico molecular docking to the M3-mAChR receptor. Moreover, all compounds possessed good pharmacokinetic parameters based on the drug-likeness rules for oral bioavailability and are considered orally active drugs. However, only compound **5** could permeate the blood–brain barrier. ADME prediction confirmed good absorption and distribution of all compounds. However, compound **2** was predicted to have low cellular permeability compared to other metabolites. Although the biological investigations in this study were preliminary, they shed some light on the anticancer potential of the metabolites. These results encouraged us for future in vivo investigation of the cytotoxic effects of these compounds and to elaborate on the mechanistic mode of action of the most promising compounds.

## Figures and Tables

**Table 1:** In vitro cytotoxic activity (IC_50_ ± SD) of arecoline hydrobromide and compounds **1**–**5** using non-small-cell lung cancer A549 and leukemia cancer cell K562.

Compound	Cytotoxicity IC_50_ (µM) ± SD	
	Non-Small-Cell Lung Cancer A549	Leukemia Cancer Cell K562
Arecoline hydrobromide	11.73 ± 0.71	15.30 ± 1.08
**1**	3.08 ± 0.19	1.56 ± 0.11
**2**	>100	>100
**3**	7.33 ± 0.45	3.33 ± 0.24
**4**	90.90 ± 0.59	67.30 ± 0.53
**5**	3.29 ± 0.20	2.15 ± 0.15
Staurosporine	10.47 ± 0.64	5.07 ± 0.36
Doxorubicin	5.05 ± 0.13	6.94 ± 0.21

**Table 2:** Cytotoxicity and selectivity index (SI) of **1**, **3**, and **5** using normal lung cell WI38 and normal blood cell PCS-800-016 in vitro.

Compound	Normal Lung Cell Line WI38 IC_50_ (µM) ± SD	** SI (Lung)	Normal Blood Cell PCS-800-016 IC_50_ (µM) ± SD	** SI (Blood Cell)
**1**	18.27 ± 0.91	5.93	9.872 ± 0.49	6.32
**3**	26.58 ± 1.33	3.64	37.86 ± 1.89	11.37
**5**	13.91 ± 0.69	5.00	20.20 ± 1.01	9.35
Staurosporine	16.42 ± 0.82	1.57	12.99 ± 0.65	2.56

** SI: selectivity index, it was calculated by dividing the IC_50_ against normal cell line by the IC_50_ against the cancer cells.

## Data Availability

Data is contained within the article and Supplementary Materials.

## Supplementary Materials

The supporting information can be downloaded at: https://www.mdpi.com/article/10.3390/ph15101171/s1, Figure S1: ^13^C NMR spectrum of 1 (CDCl_3_, 100 MHz), Figure S2: ^1^H NMR spectrum of 1 (CDCl_3_, 400 MHz), Figure S3: COSY spectrum of 1 (CDCl_3_), Figure S4: HSQC spectrum of 1 (CDCl_3_), Figure S5: HMBC spectrum of 1 (CDCl_3_), Figure S6: NOE spectrum of 1 (CDCl_3_), Figure S7: IR spectrum of 1 (KBr disc), Figure S8: ESIMS (-ve) spectrum of 1, Figure S9: DEPTQ-135 NMR spectrum of 2 (D_2_O, 100 MHz), Figure S10: ^1^H NMR spectrum of 2 (D_2_O, 400 MHz), Figure S11: COSY spectrum of 2 (D_2_O), Figure S12: DEPTQ-135 and ^1^H NMR correlation spectrum of 2 (D_2_O), Figure S13: HMBC spectrum of 2 (D_2_O), Figure S14: NOE spectrum of 2 (D_2_O), Figure S15: IR spectrum of 2 (KBr disc), Figure S16: HRESIMS (+ve) spectrum of 2, Figure S17: DEPTQ-135 spectrum of 3 (D_2_O, 100 MHz), Figure S18: ^1^H NMR spectrum of 3 (D_2_O, 400 MHz), Figure S19: COSY spectrum of 3 (D_2_O), Figure S20: DEPTQ-135 and ^1^H NMR correlation spectrum of 3 (D_2_O), Figure S21: HMBC spectrum of 3 (D_2_O), Figure S22: NOE spectrum of 3 (D_2_O), Figure S23: IR spectrum of 3 (KBr disc), Figure S24: ESIMS (-ve) spectrum of 3, Figure S25: DEPTQ-135 spectrum of 4 (D_2_O, 100 MHz), Figure S26: ^1^H NMR spectrum of 4 (D_2_O, 400 MHz), Figure S27: COSY spectrum of 4 (D_2_O), Figure S28: DEPTQ-135 and ^1^H NMR correlation spectrum of 4 (D_2_O), Figure S29: HMBC spectrum of 4 (D_2_O), Figure S30: IR spectrum of 4 (KBr disc), Figure S31: ESIMS (+ve) spectrum of 4, Figure S32: ^13^C NMR spectrum of 5 (D_2_O, 100 MHz), Figure S33: ESIMS (-ve) spectrum of 5, Figure S34: ^1^H NMR spectrum of 5 (D_2_O, 400 MHz), Figure S35: COSY spectrum of 5 (D_2_O), Figure S36: HSQC spectrum of 5 (D_2_O), Figure S37: HMBC spectrum of 5 (D_2_O), Figure S38: IR spectrum of 5 (KBr disc), Figure S39: The binding mode of arecoline, 2 and 4. Electrostatic surface representation of ligand binding pocket of arecoline (A), 2 (B) and 4 (C). Amino acid residues involved in ligand interaction, hydrogen bonds are shown as blue dashed lines, arecoline (D), 2 (E) and 4 (F), Figure S40: SwissADME prediction, BOILED egg showing blood brain barrier permeability (yellow region) and human intestinal absorption (white region). Red dots represented molecules predicted not to be effluated from central nervous system by P-glycoprotein. Arecoline (A), 1 (B), 2 (C), 3 (D), 4 (E) and 5 (F), Figure S41: Cell permeability prediction of arecoline and compounds **1**–**5** from PerMM web-based tool, Table S1: A: Effect of 1 on the cell cycle of non-small cell lung cancer A549 using untreated lung cancer cells as a negative control. B: Effect of 3 and 5 on cell cycle of leukemia cancer K562 using untreated leukemia cancer cells as a negative control, Table S2: A: Detection of apoptosis induced by 1 using non-small cell lung cancer A549 and untreated cells as a negative control. B: Detection of apoptosis induced by 3 and 5 using leukemia cancer K562 and untreated cells as a negative control. Fold increase than control was included, Table S3: Docking results of arecoline and compounds **1**–**5** to M3-mT4L (PDB ID: 4U15), Table S4: Amino acids involved in hydrogen bonding interaction of arecoline and compounds **1**–**5** with M3-mAChR, Table S5: Physicochemical properties of arecoline and compounds **1**–**5** for detection of drug likeness and oral bioavailability, Table S6: Results of ADME prediction of arecoline and compounds **1**–**5** from PreADMET web-based tool, Table S7: Cell permeability prediction of arecoline and compounds **1**–**5** using PerMM web-based tool, Table S8: List of microorganisms.
