# Supplementary material for: In Vitro Characterization of Inhibitors for Lung A549 and Leukemia K562 Cell Lines from Fungal Transformation of Arecoline Supported by In Silico Docking to M3-mAChR and ADME Prediction
Source: Pharmaceuticals (Basel). 2022 Sep 21;15(10):1171. doi: 10.3390/ph15101171 (PMC9609924; doi:10.3390/ph15101171)
Supplement: Supplementary file 1 [file pharmaceuticals-15-01171-s001.zip › pharmaceuticals-1890429-supplementary.pdf]

In vitro characterization of inhibitors for lung A549 and leukemia K562 cell lines from fungal transformation of arecoline: Apoptosis mechanism, targeting M3-mAChR via *in silico* study and ADME prediction

Amany E. Ragab[a], Ebtisam T. Badawy[a], Shaimaa M. Aboukhatwa[b,c], Amal Kabbash[a] and Kamilia A. Abo Elseoud[a].

[a]Department of Pharmacognosy, Faculty of Pharmacy, Tanta University, Tanta, 31527, Egypt.
[b]Department of Pharmaceutical Chemistry, Faculty of Pharmacy, Tanta University, Tanta, 31527, Egypt.
[c]Department of Pharmaceutical Sciences, College of Pharmacy, University of Illinois at Chicago, Chicago Illinois, 60612, USA.

[*] Correspondence: amany.ragab@pharm.tanta.edu.eg

Content	
**Subject**	**Page**

Subject	Page
**Fig. S1:** ^{13}C NMR spectrum of **1** ($CDCl_3$, 100 MHz).	5
**Fig. S2:** ^{1}H NMR spectrum of **1** ($CDCl_3$, 400 MHz).	6
**Fig. S3:** COSY spectrum of **1** ($CDCl_3$).	7
**Fig. S4:** HSQC spectrum of **1** ($CDCl_3$).	8
**Fig. S5:** HMBC spectrum of **1** ($CDCl_3$).	9
**Fig. S6:** NOE spectrum of **1** ($CDCl_3$).	10
**Fig. S7:** IR spectrum of **1** (KBr disc).	11
**Fig. S8:** ESIMS (-ve) spectrum of **1**.	12
**Fig. S9:** DEPTQ-135 NMR spectrum of **2** (D_2O, 100 MHz).	13
**Fig. S10:** ^{1}H NMR spectrum of **2** (D_2O, 400 MHz).	14
**Fig. S11:** COSY spectrum of **2** (D_2O).	15
**Fig. S12:** DEPTQ-135 and ^{1}H NMR correlation spectrum of **2** (D_2O).	16
**Fig. S13:** HMBC spectrum of **2** (D_2O).	17
**Fig. S14:** NOE spectrum of **2** (D_2O).	18
**Fig. S15:** IR spectrum of **2** (KBr disc).	19
**Fig. S16:** HRESIMS (+ve) spectrum of **2**.	20
**Fig. S17:** DEPTQ-135 spectrum of **3** (D_2O, 100 MHz).	21
**Fig. S18:** ^{1}H NMR spectrum of **3** (D_2O, 400 MHz).	22
**Fig. S19:** COSY spectrum of **3** (D_2O).	23
**Fig. S20:** DEPTQ-135 and ^{1}H NMR correlation spectrum of **3** (D_2O).	24
**Fig. S21:** HMBC spectrum of **3** (D_2O).	25
**Fig. S22:** NOE spectrum of **3** (D_2O).	26
**Fig. S23:** IR spectrum of **3** (KBr disc).	27
**Fig. S24:** ESIMS (-ve) spectrum of **3**.	28
**Fig. S25:** DEPTQ-135 spectrum of **4** (D_2O, 100 MHz).	29

**Fig. S26:** ^{1}H NMR spectrum of **4** (D_2O, 400 MHz).	30
**Fig. S27:** COSY spectrum of **4** (D_2O).	31
**Fig. S28:** DEPTQ-135 and ^{1}H NMR correlation spectrum of **4** (D_2O).	32
**Fig. S29:** HMBC spectrum of **4** (D_2O).	33
**Fig. S30:** IR spectrum of **4** (KBr disc).	34
**Fig. S31:** ESIMS (+ve) spectrum of **4**.	35
**Fig. S32:** ^{13}C NMR spectrum of **5** (D_2O, 100 MHz).	36
**Fig. S33:** ESIMS (-ve) spectrum of **5.**	37
**Fig. S34:** ^{1}H NMR spectrum of **5** (D_2O, 400 MHz).	38
**Fig. S35:** COSY spectrum of **5** (D_2O).	39
**Fig. S36:** HSQC spectrum of **5** (D_2O).	40
**Fig. S37:** HMBC spectrum of **5** (D_2O).	41
**Fig. S38:** IR spectrum of **5** (KBr disc).	42
**Fig. S39:** The binding mode of **arecoline**, **2** and **4**. Electrostatic surface representation of ligand binding pocket of **arecoline** (A), **2** (B) and **4** (C). Amino acid residues involved in ligand interaction, hydrogen bonds are shown as blue dashed lines, **arecoline** (D), **2** (E) and **4** (F).	43
**Fig. S40:** SwissADME prediction, BOILED egg showing blood brain barrier permeability (yellow region) and human intestinal absorption (white region). Red dots represented molecules predicted not to be effluated from central nervous system by P-glycoprotein. **Arecoline** (A), **1** (B)**, 2** (C), **3** (D), **4** (E) and **5** (F).	44
**Fig. S41:** Cell permeability prediction of arecoline and compounds **1-5** from PerMM web-based tool.	45
**Table S1: A:** Effect of **1** on the cell cycle of non-small cell lung cancer A549, using untreated lung cancer cells as a negative control. **B:** Effect of **3** and **5** on cell cycle of leukemia cancer K562, using untreated leukemia cancer cells as a negative control.	46

**Table S2**: **A**: Detection of apoptosis induced by **1** using non-small cell lung cancer A549 and untreated cells as a negative control. **B**: Detection of apoptosis induced by **3** and **5** using leukemia cancer K562 and untreated cells as a negative control. Fold increase than control was included.	47
**Table S3:** Docking results of arecoline and compounds **1-5** to M3-mT4L (PDB ID: 4U15).	48
**Table S4:** Amino acids involved in hydrogen bond interaction of arecoline and compounds **1-5** with M3-mT4L.	49
**Table S5:** Physicochemical properties of arecoline and compounds **1-5** for detection of drug likeness and oral bioavailability.	50
**Table S6:** Results of ADME prediction of arecoline and compounds **1-5** from PreADMET web-based tool.	51
**Table S7:** Cell permeability prediction of arecoline and compounds **1-5** using from PerMM web-based tool	52
**Table S8:** List of Microorganisms.	53

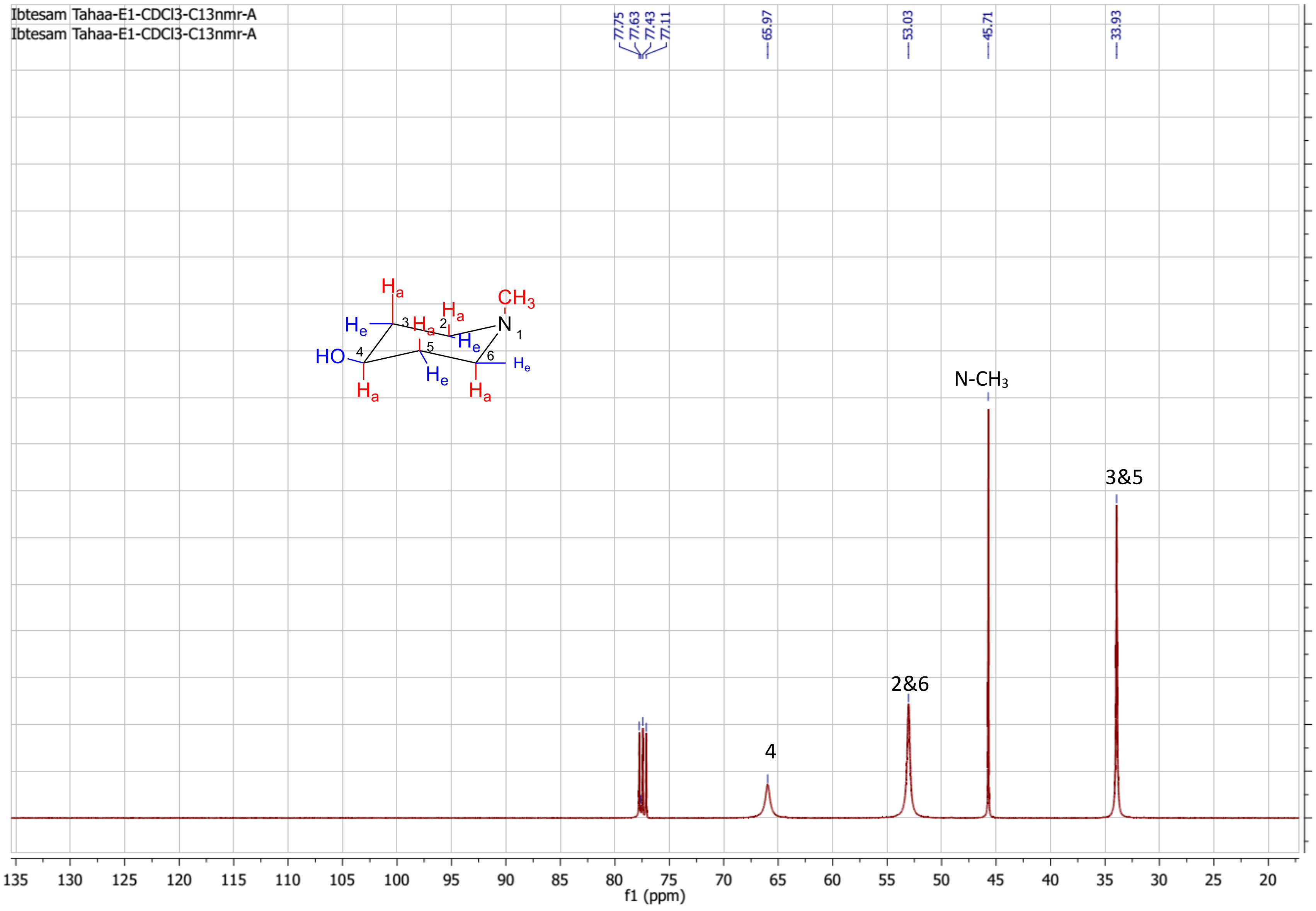

Fig. S1: ^{13}C NMR spectrum of **1** ($CDCl_3$, 100 MHz).

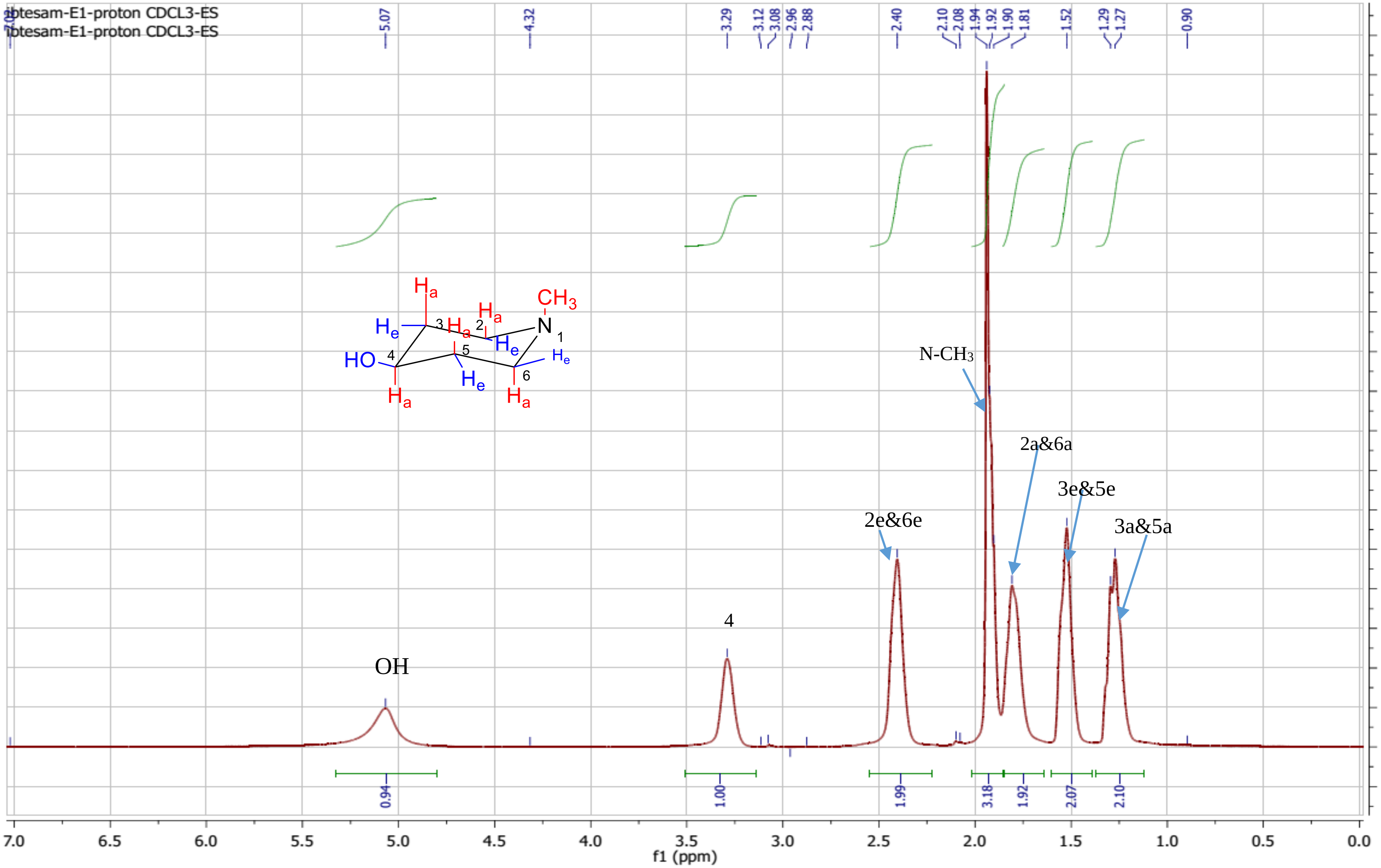

Fig. S2: ^{1}H NMR spectrum of **1** ($CDCl_3$, 400 MHz).

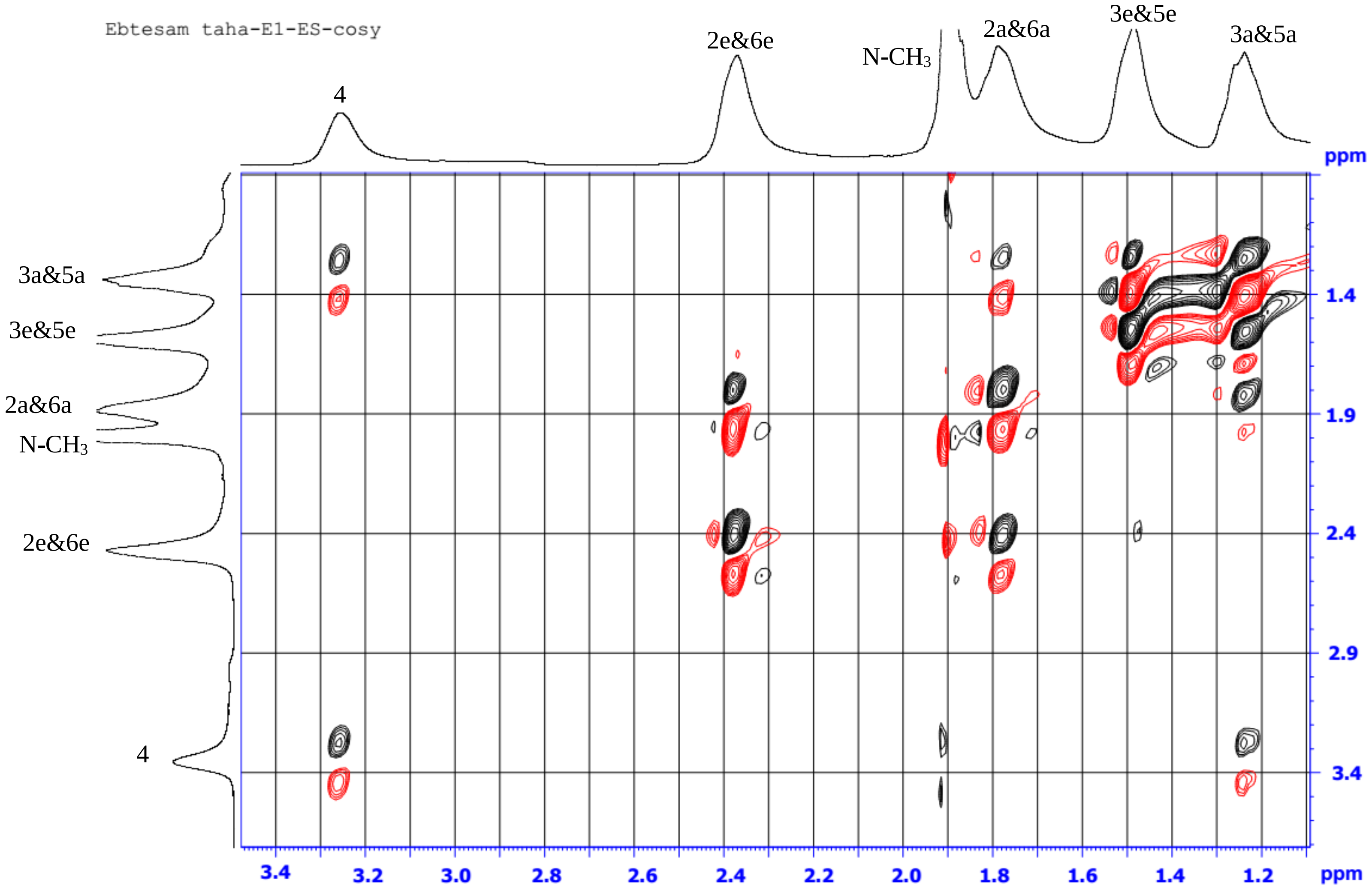

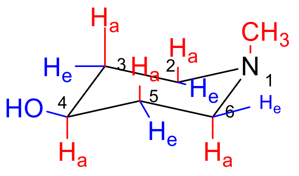

Fig. S3: COSY spectrum of **1** ($CDCl_3$).

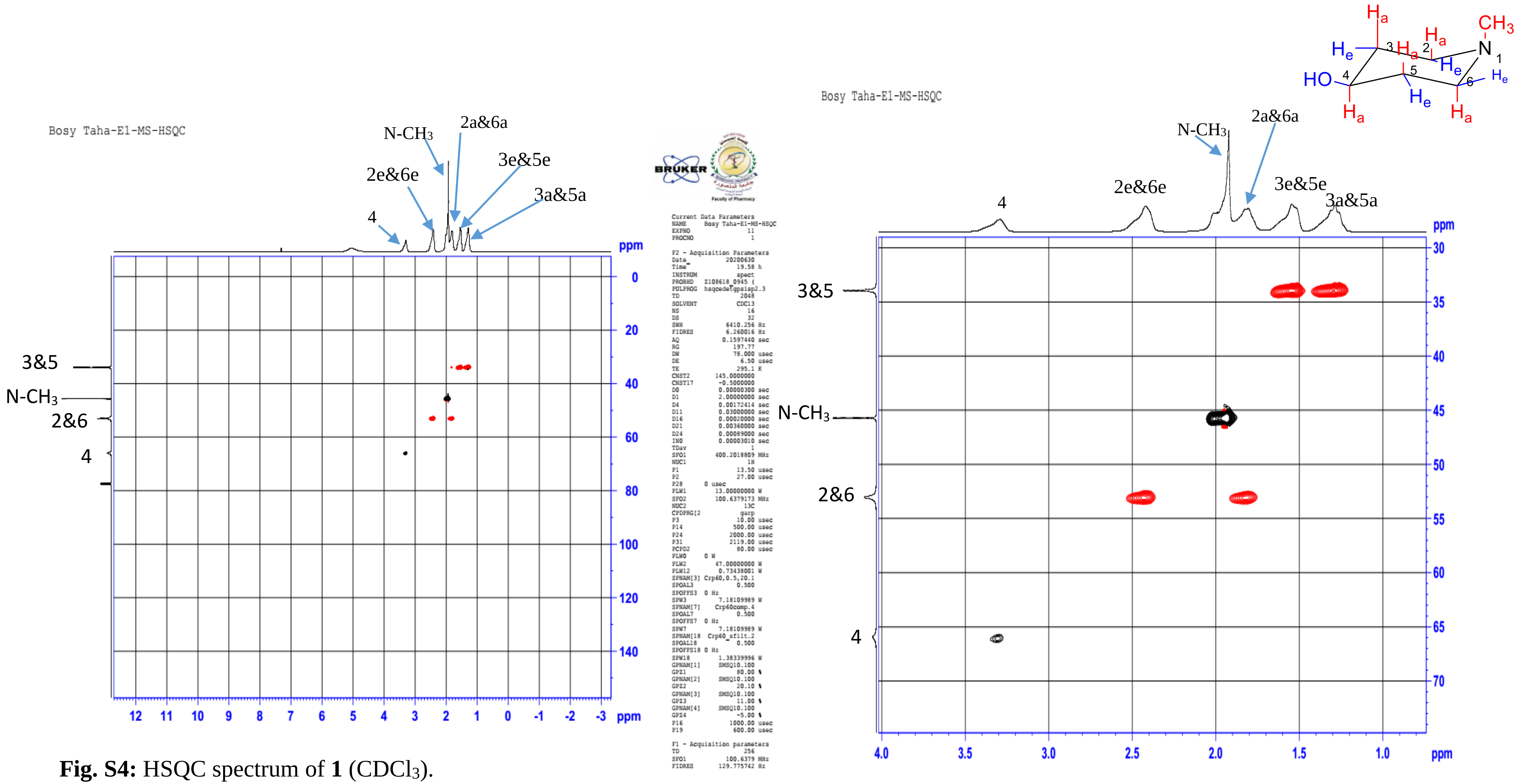

Fig. S4: HSQC spectrum of **1** ($CDCl_3$).

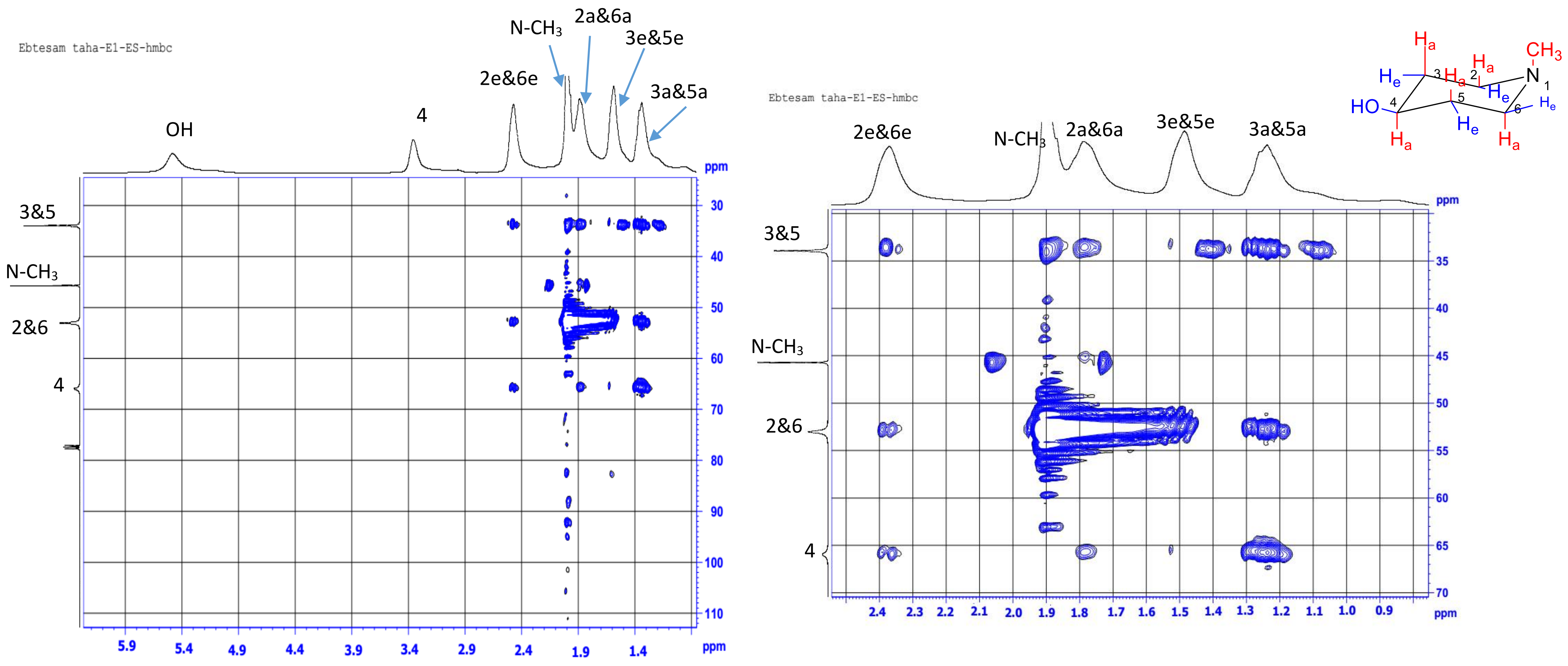

Fig. S5: HMBC spectrum of **1** ($CDCl_3$).

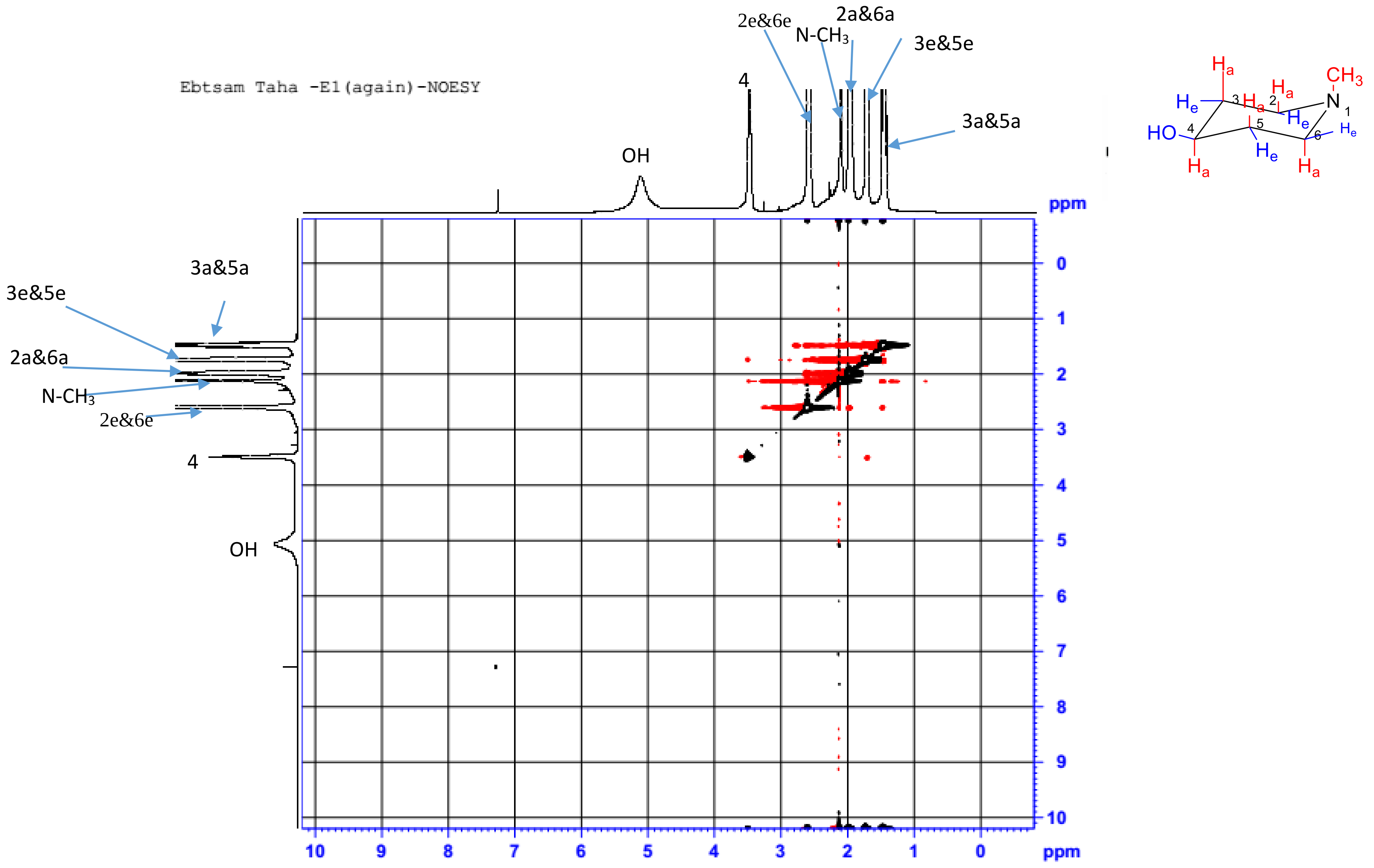

Fig. S6: NOE spectrum of **1** ($CDCl_3$).

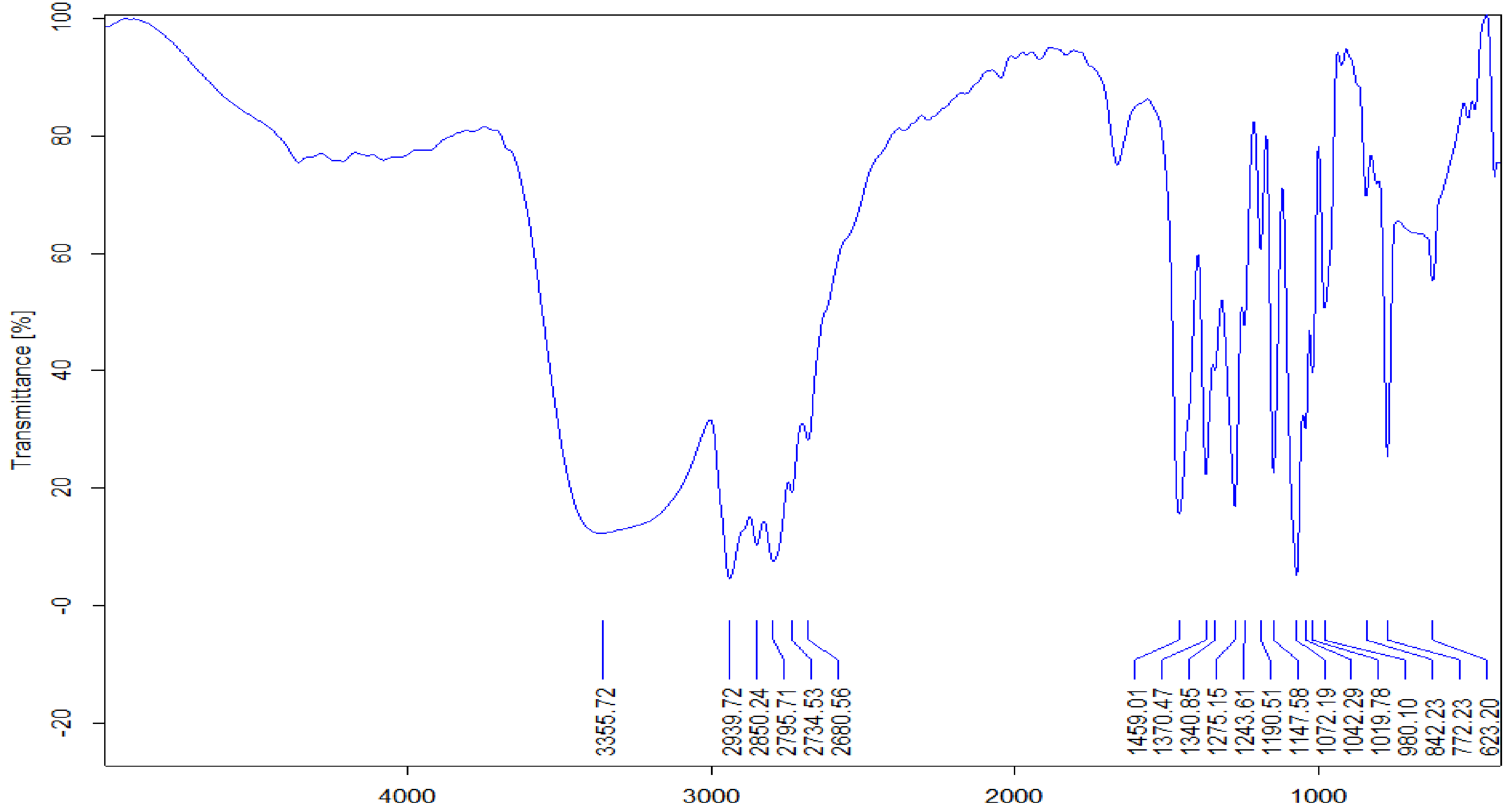

Fig. S7: IR spectrum of **1** (KBr disc).

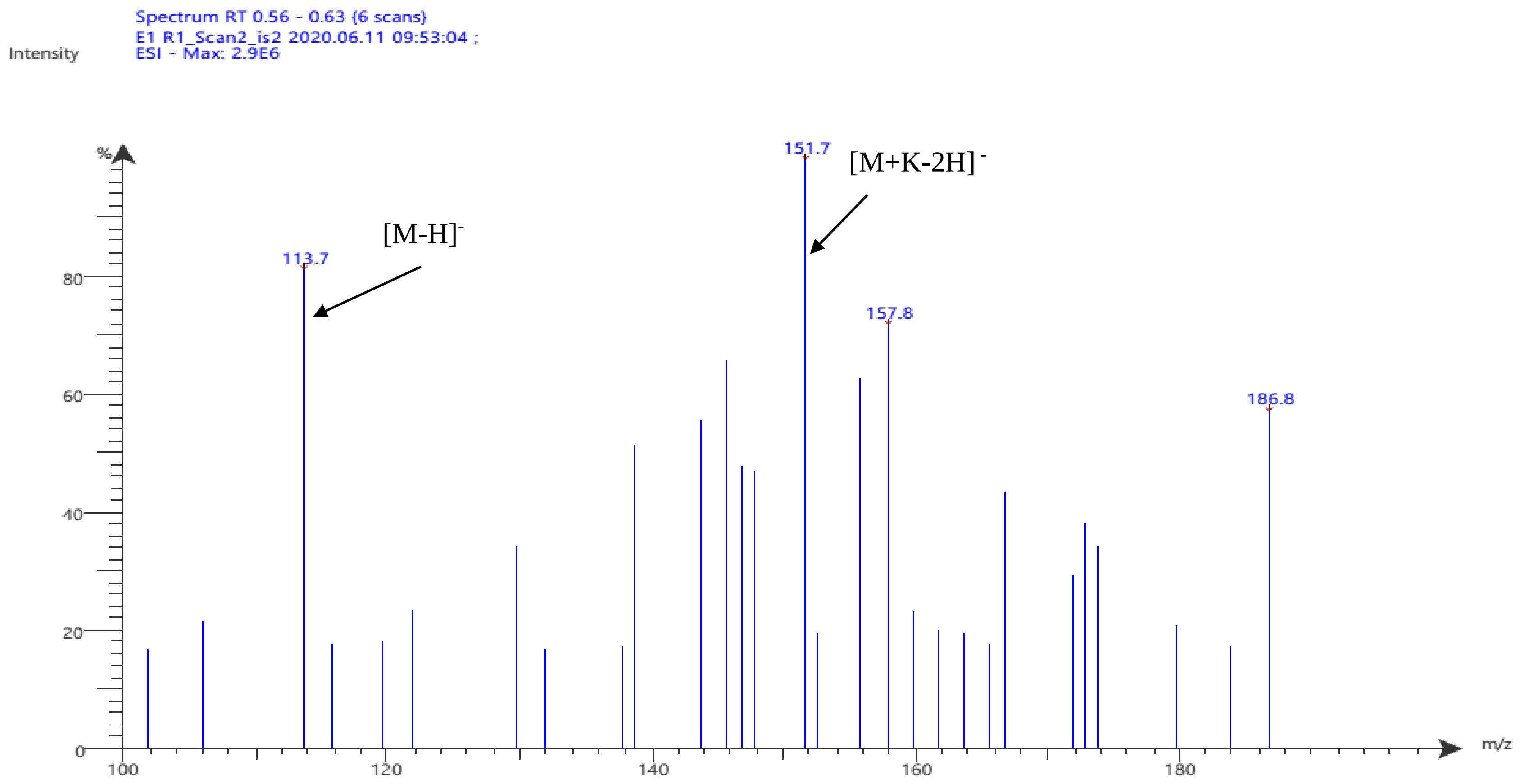

Fig. S8: ESIMS (-ve) spectrum of **1**.

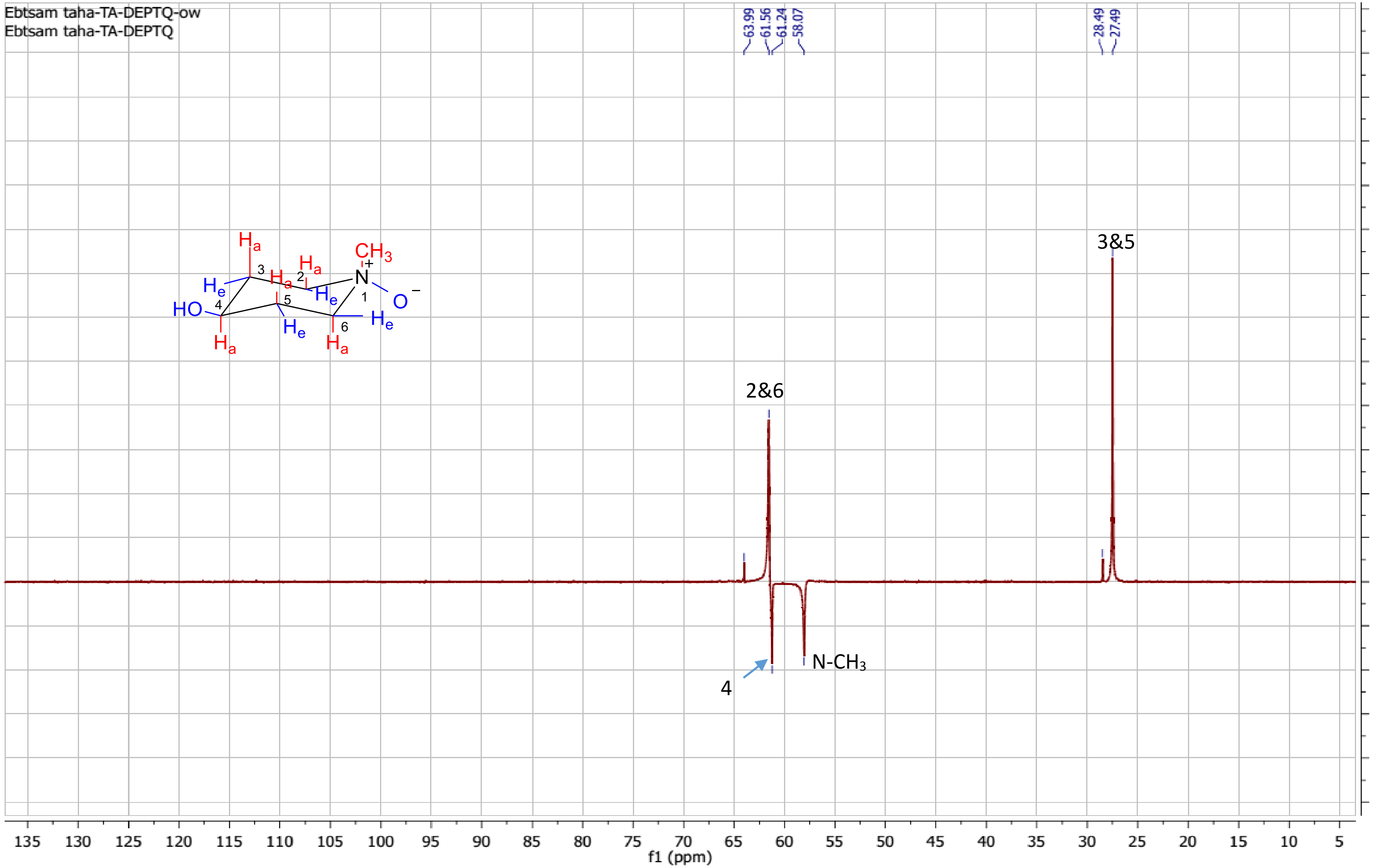

Fig. S9: DEPTQ-135 NMR spectrum of **2** (D_2O, 100 MHz).

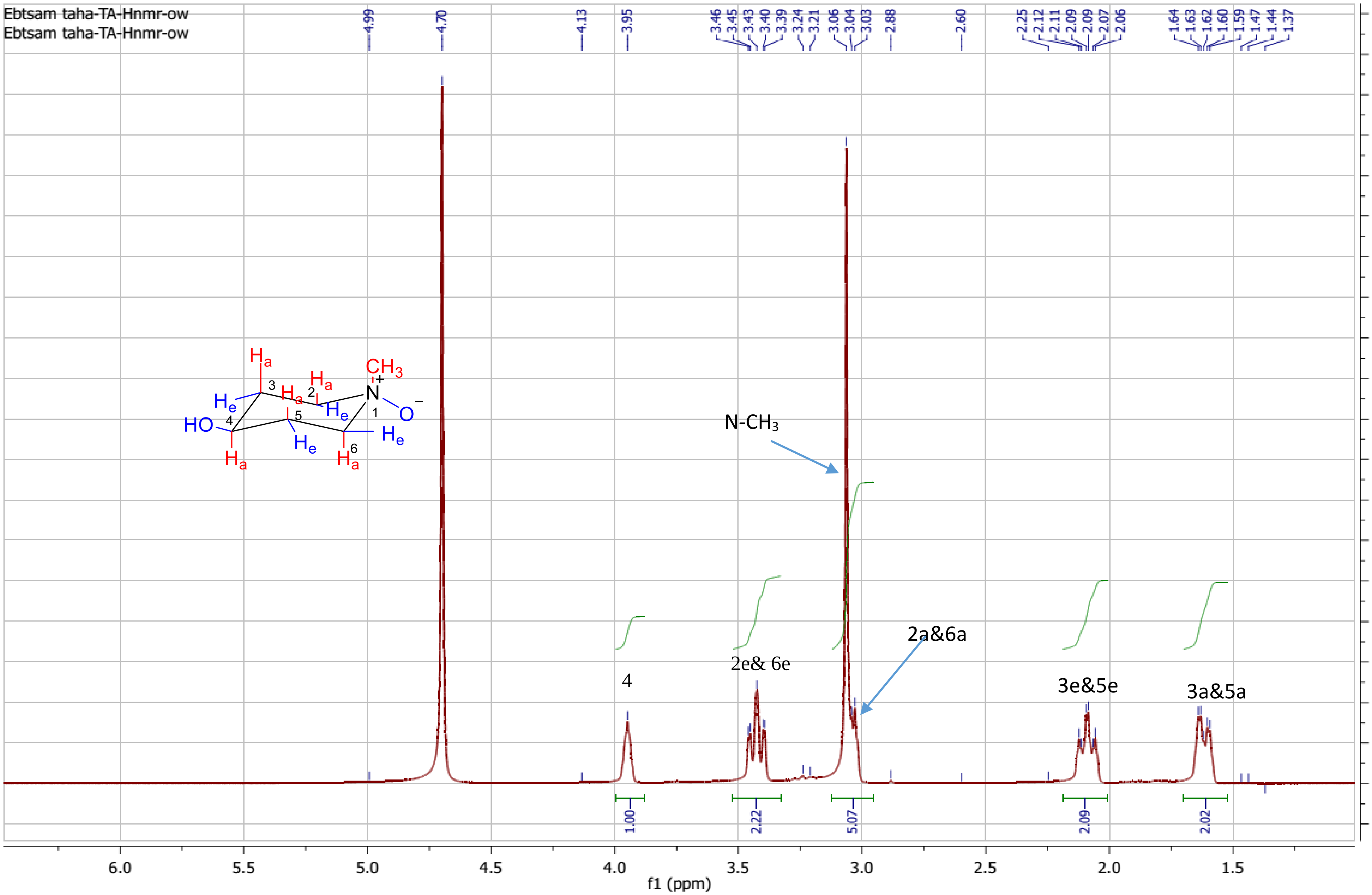

Fig. S10: ^{1}H NMR spectrum of **2** (D_2O, 400 MHz).

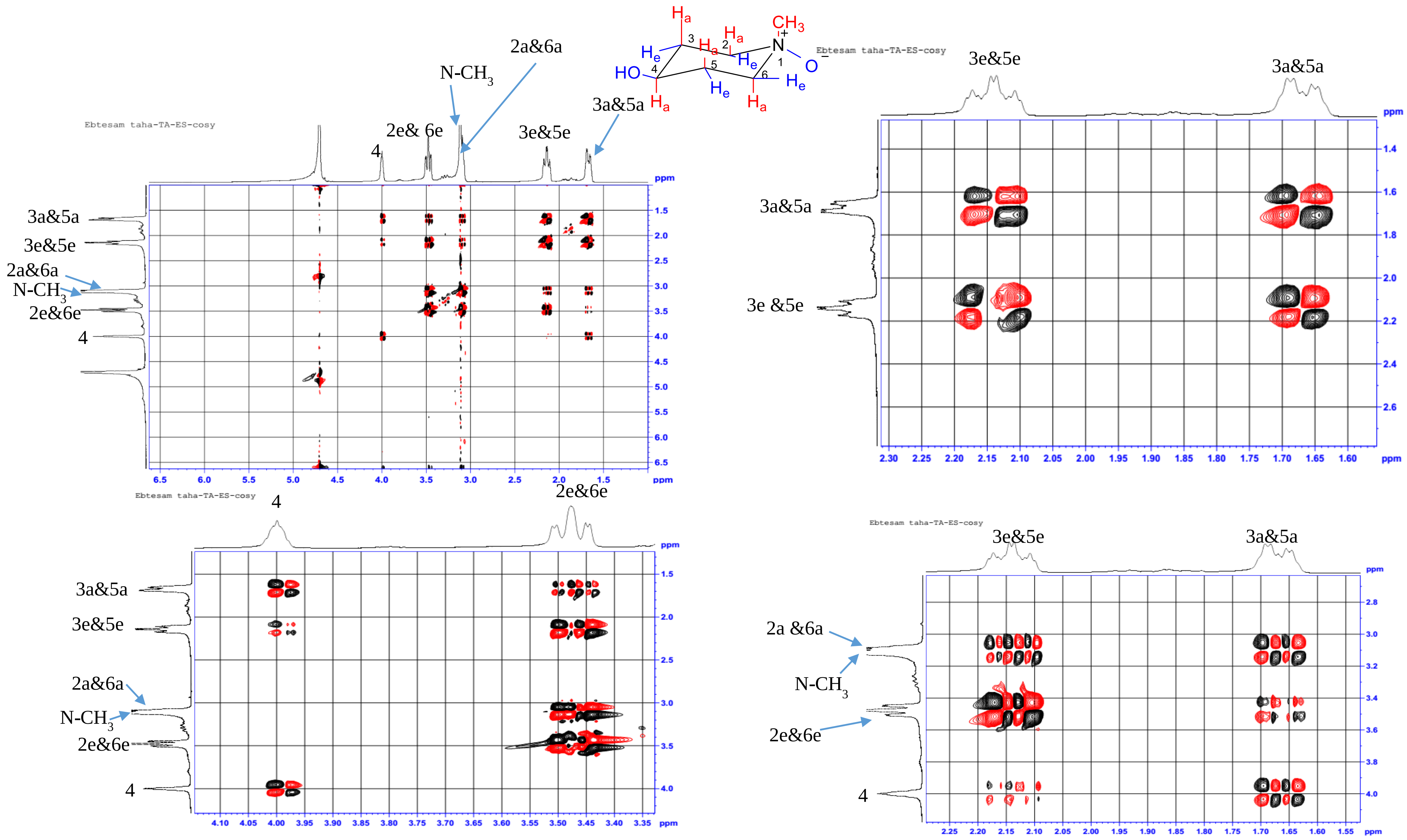

Fig. S11: COSY spectrum of **2** (D_2O).

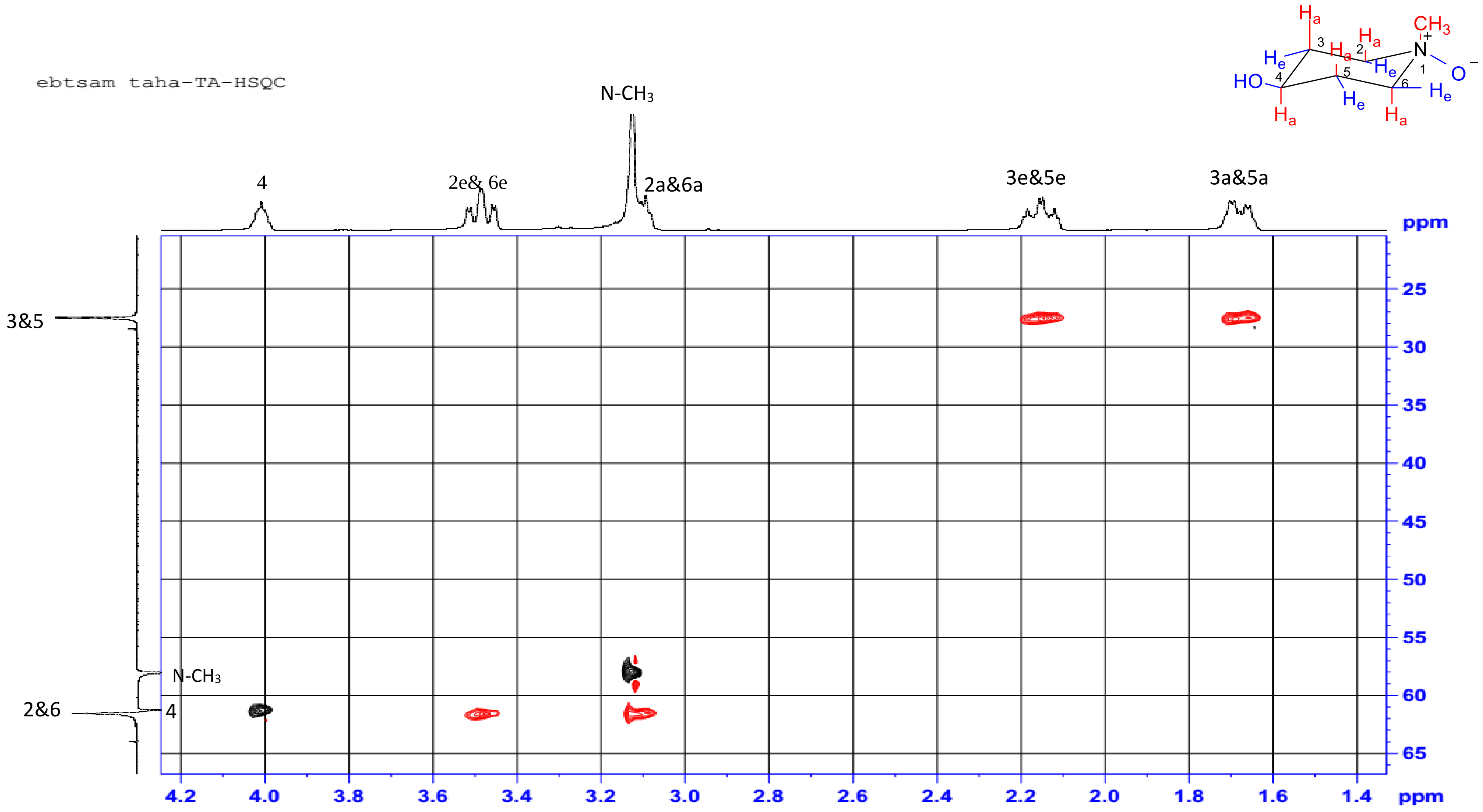

Fig. S12: DEPTQ-135 and ^{1}H NMR correlation spectrum of **2** (D_2O).

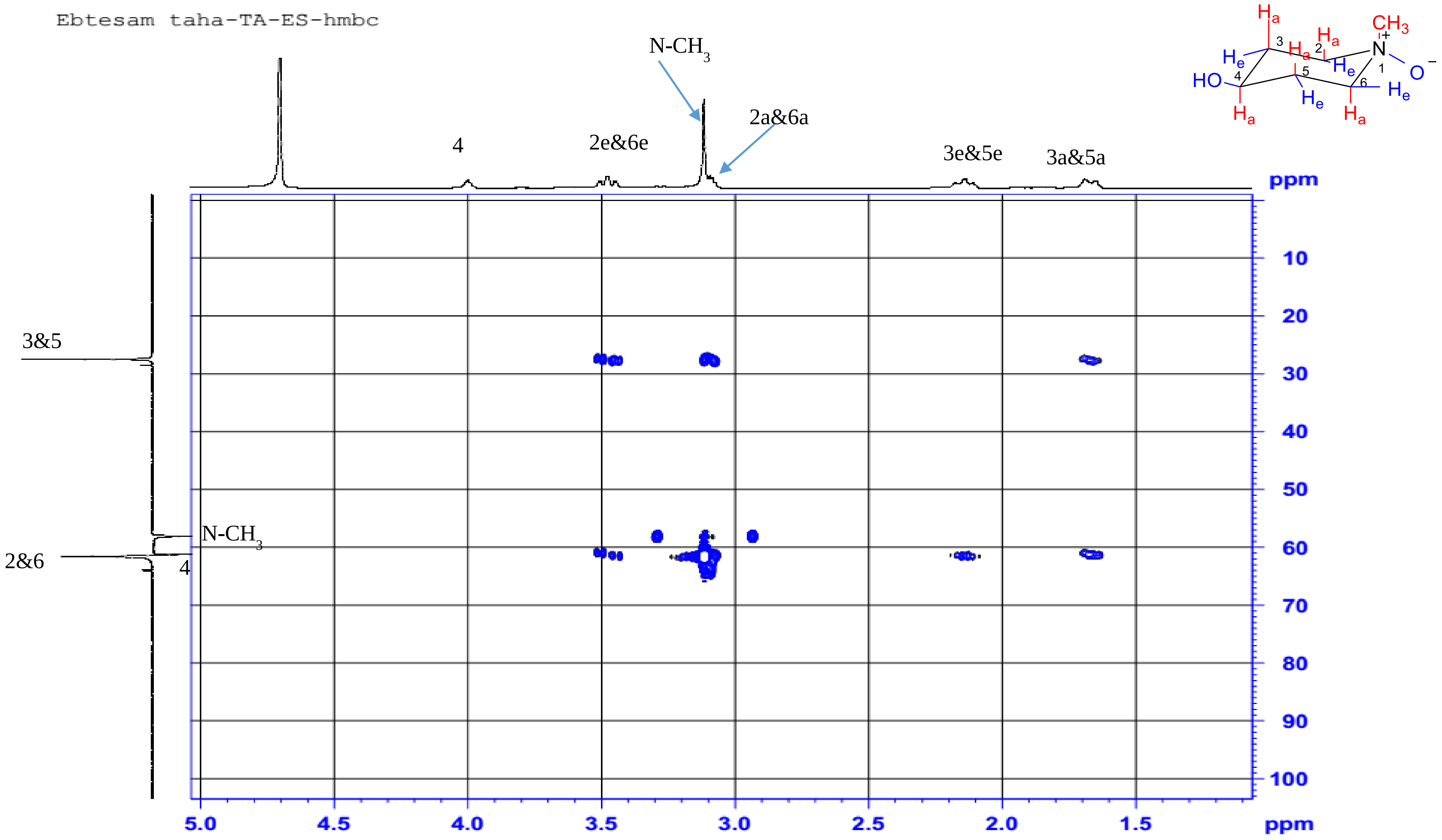

Fig. S13: HMBC spectrum of **2** (D_2O).

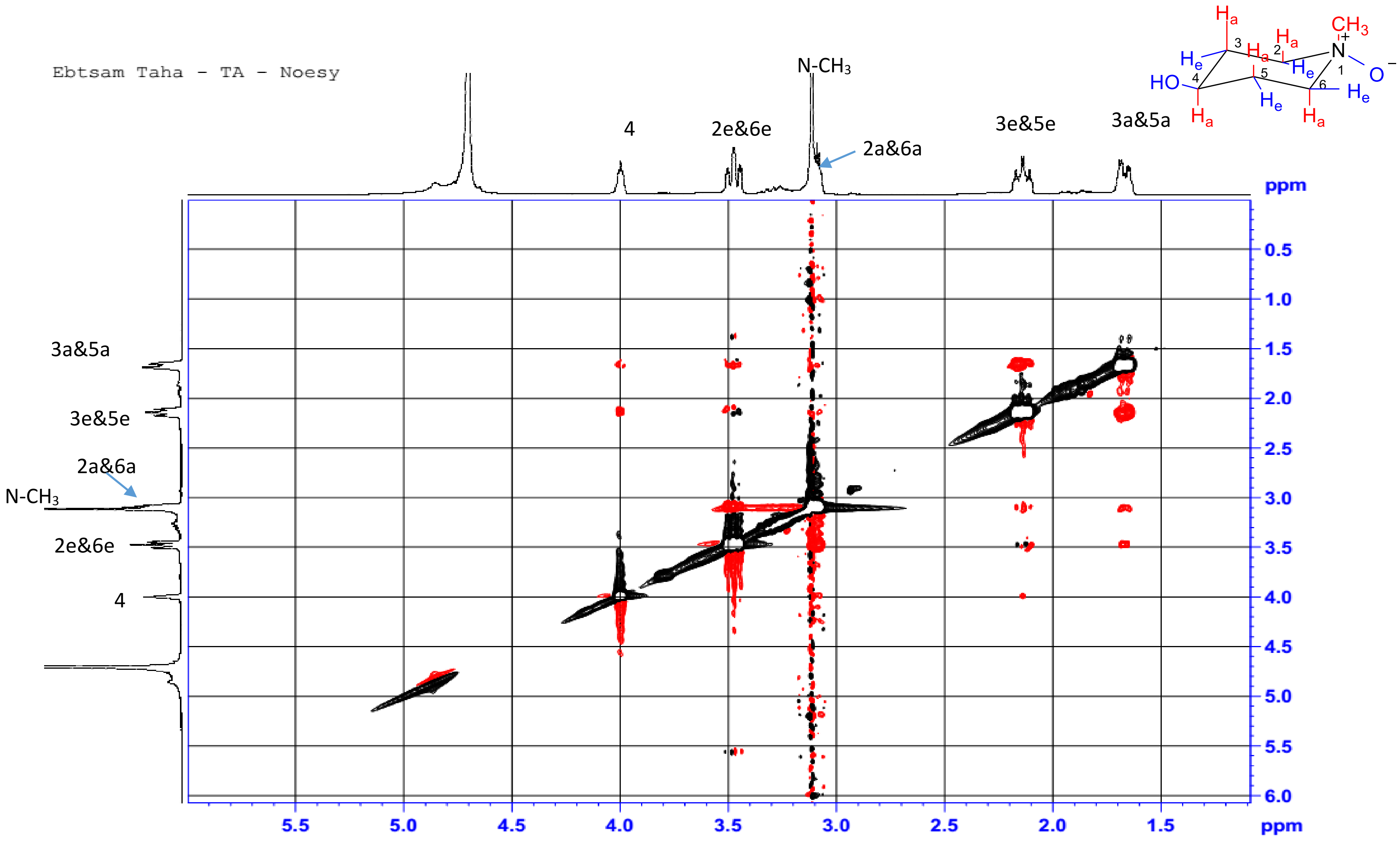

Fig. S14: NOE spectrum of **2** (D_2O).

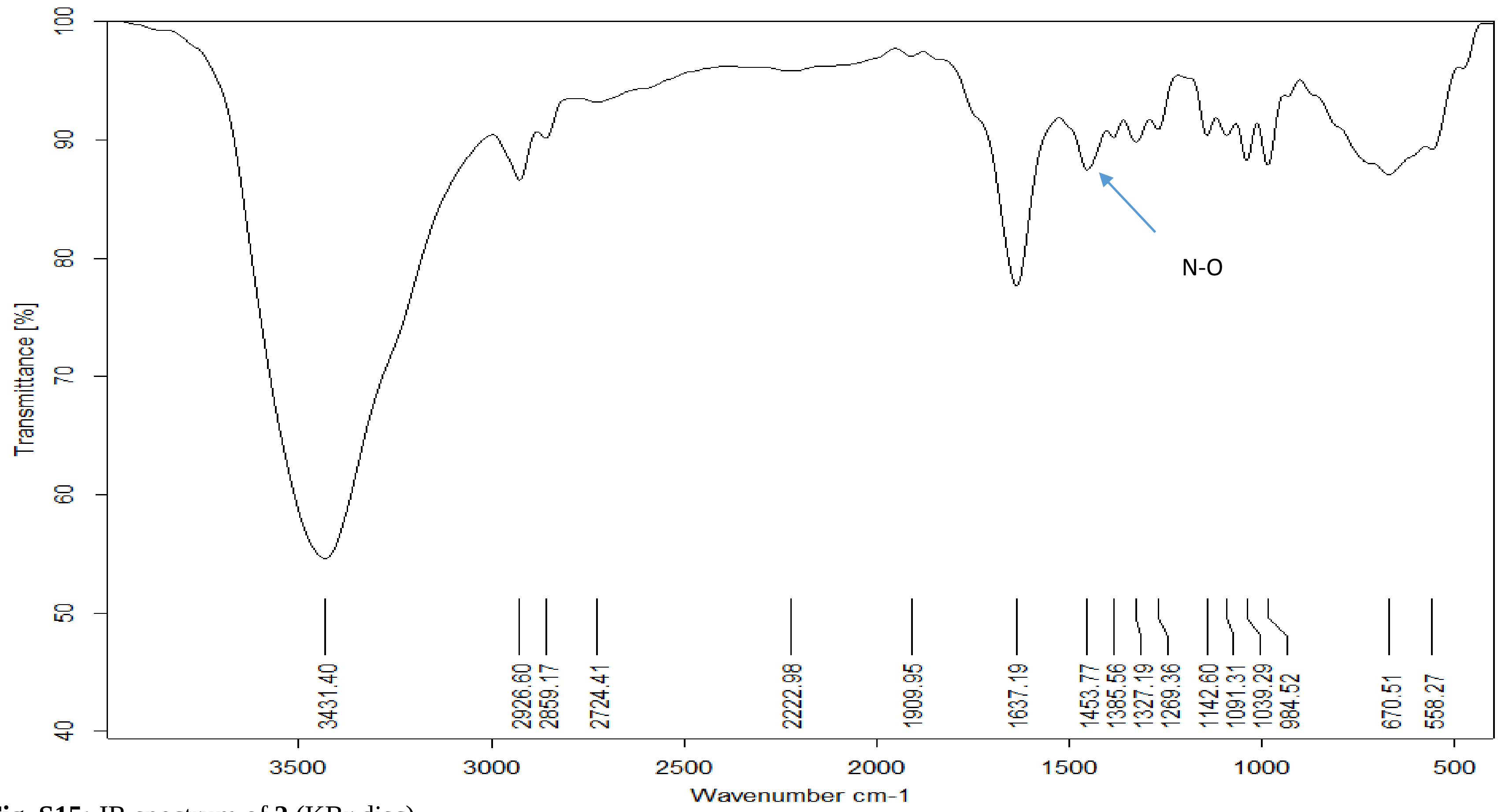

Fig. S15: IR spectrum of **2** (KBr disc).

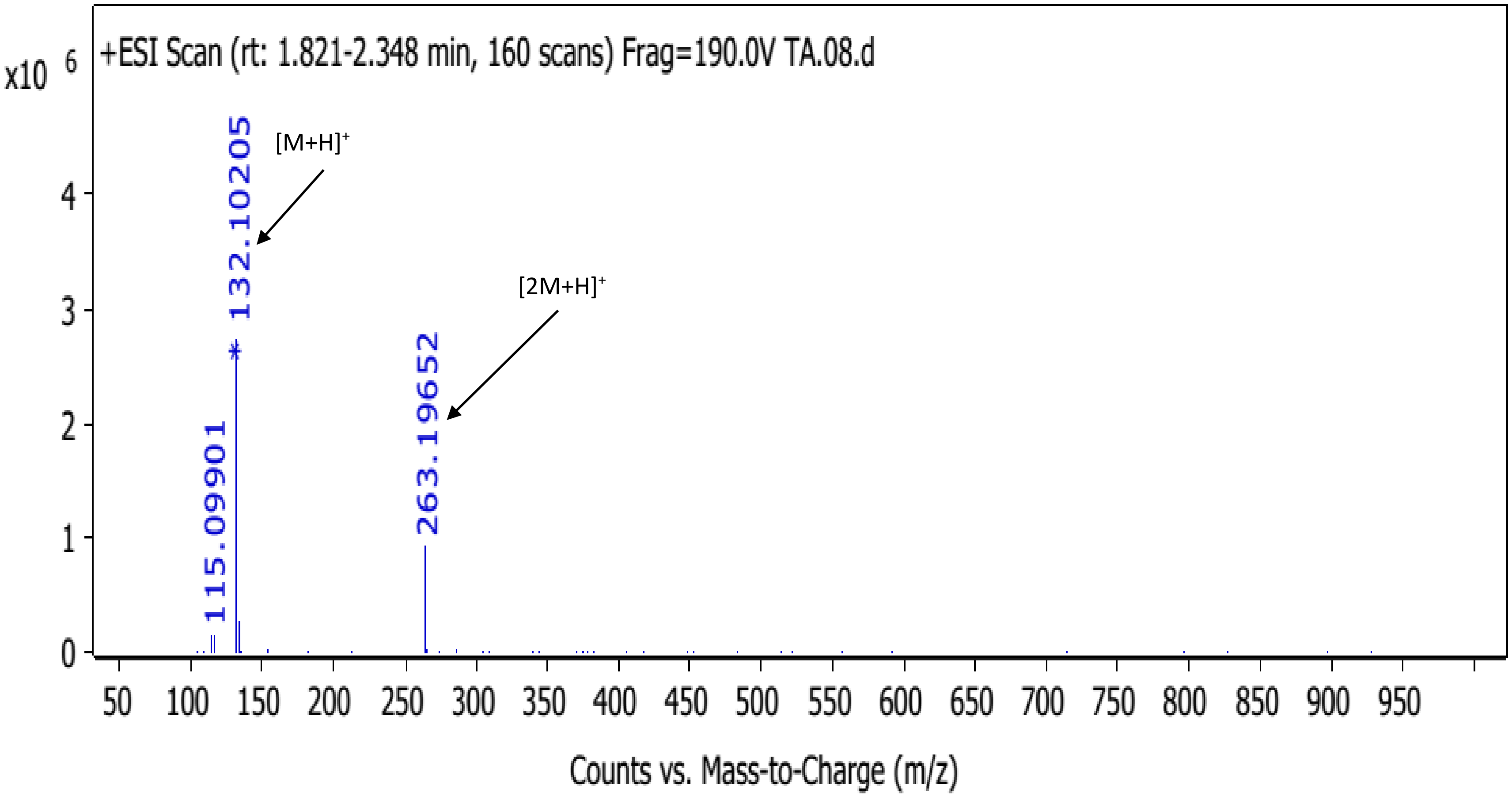

Fig. S16: HRESIMS (+ve) spectrum of **2**.

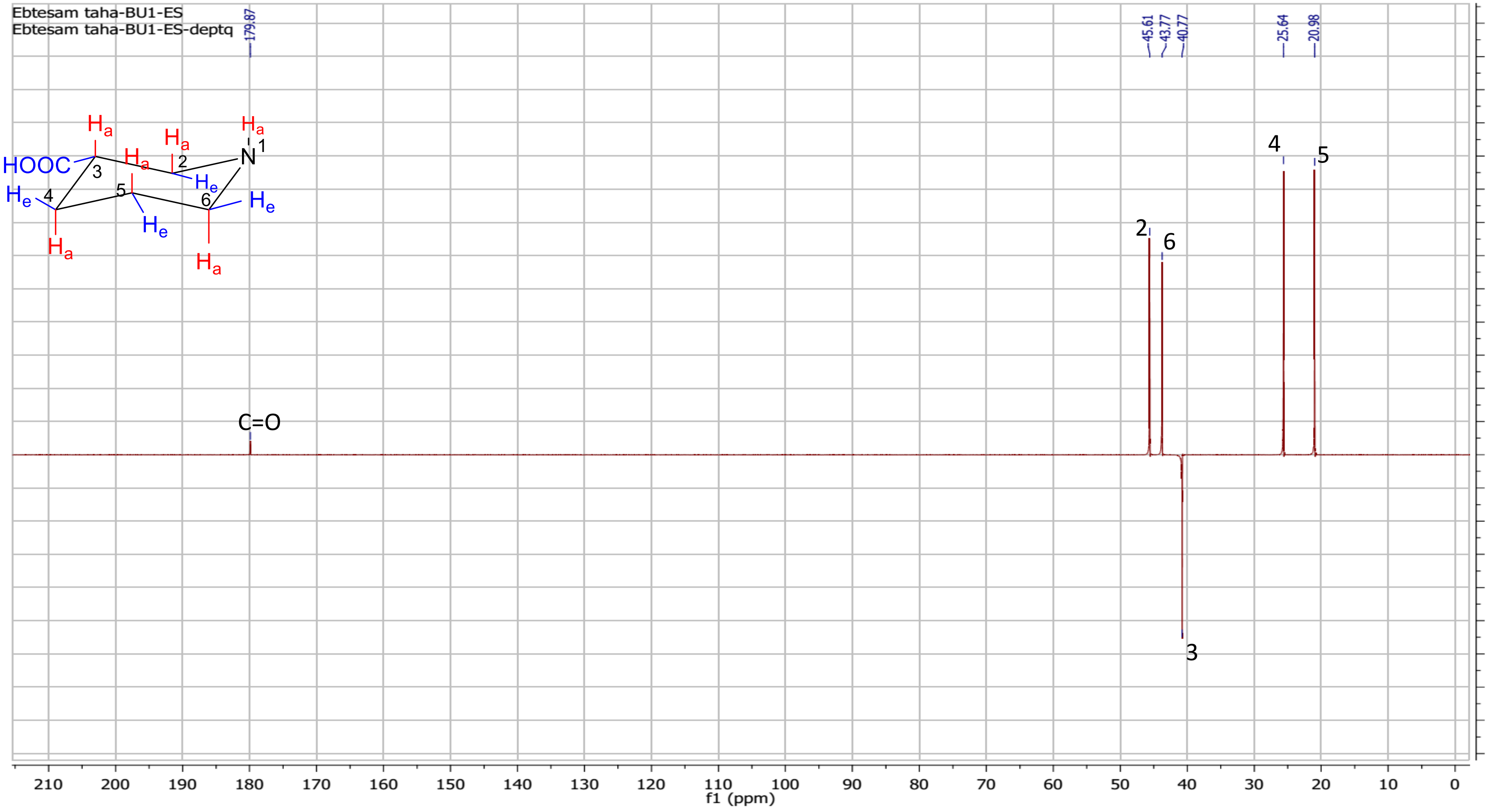

Fig. S17: DEPTQ-135 spectrum of **3** (D_2O, 100 MHz).

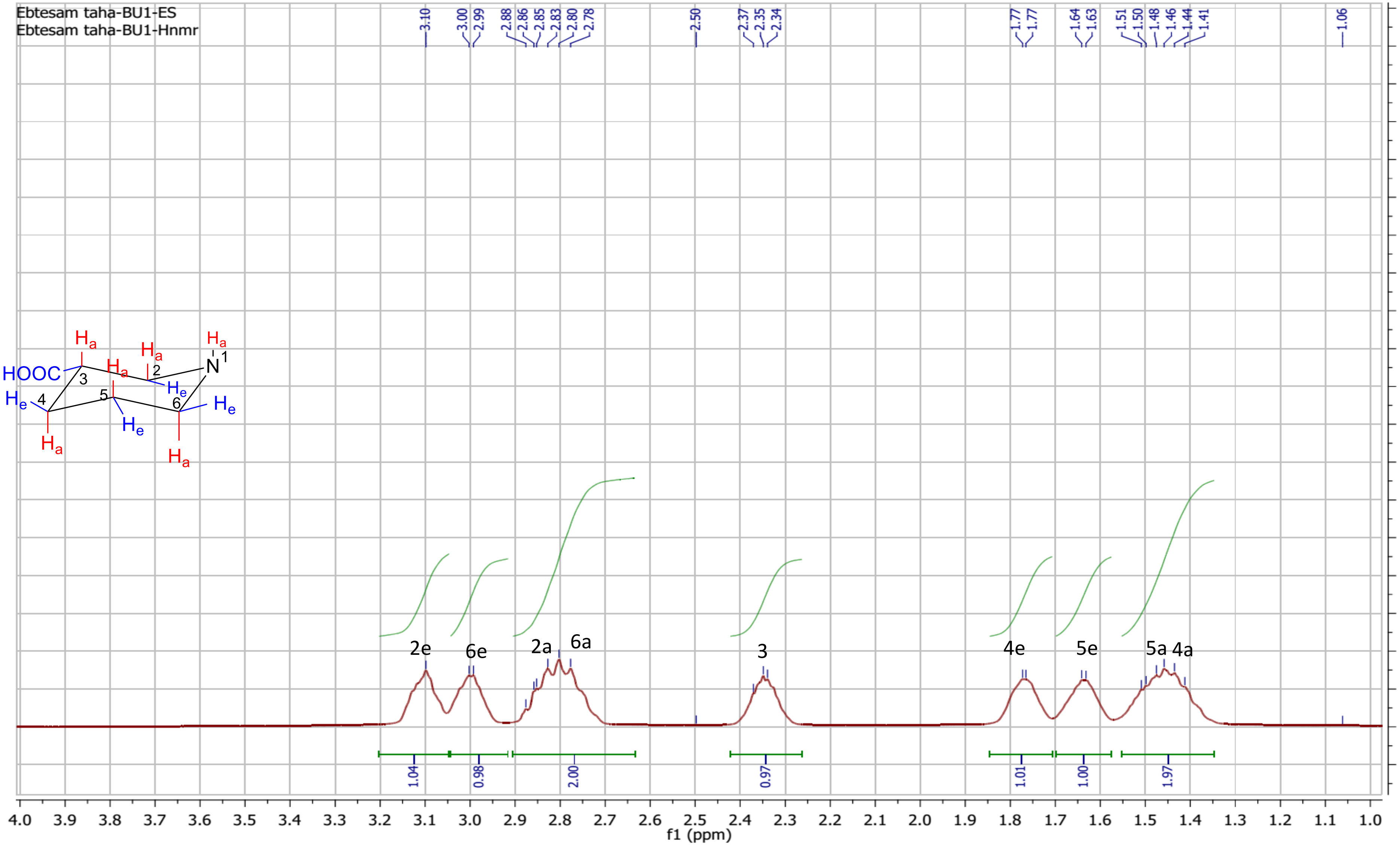

Fig. S18: ^{1}H NMR spectrum of **3** (D_2O, 400 MHz).

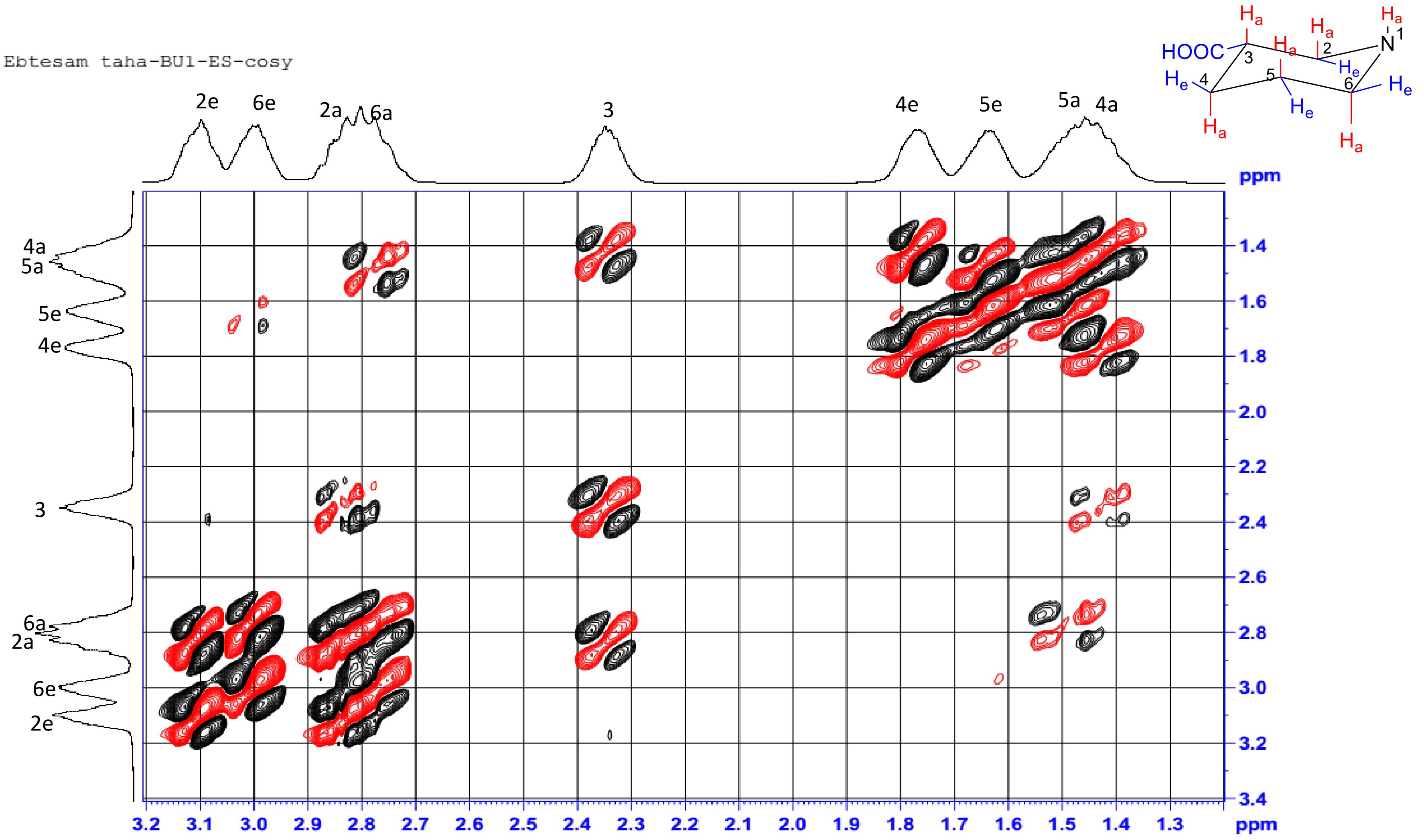

Fig. S19: COSY spectrum of **3** (D_2O).

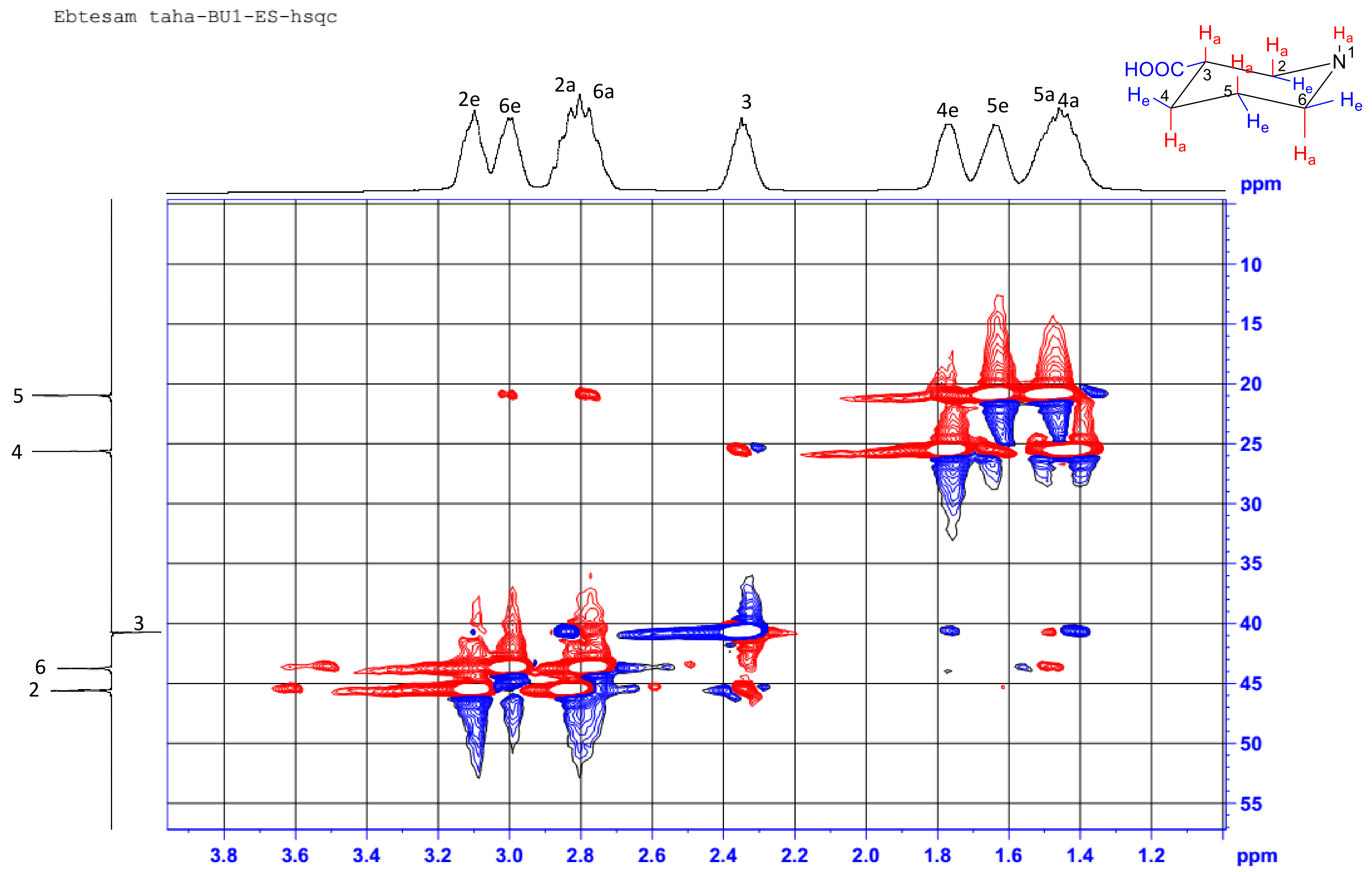

Fig. S20: DEPTQ-135 and ^{1}H NMR correlation spectrum of **3** (D_2O).

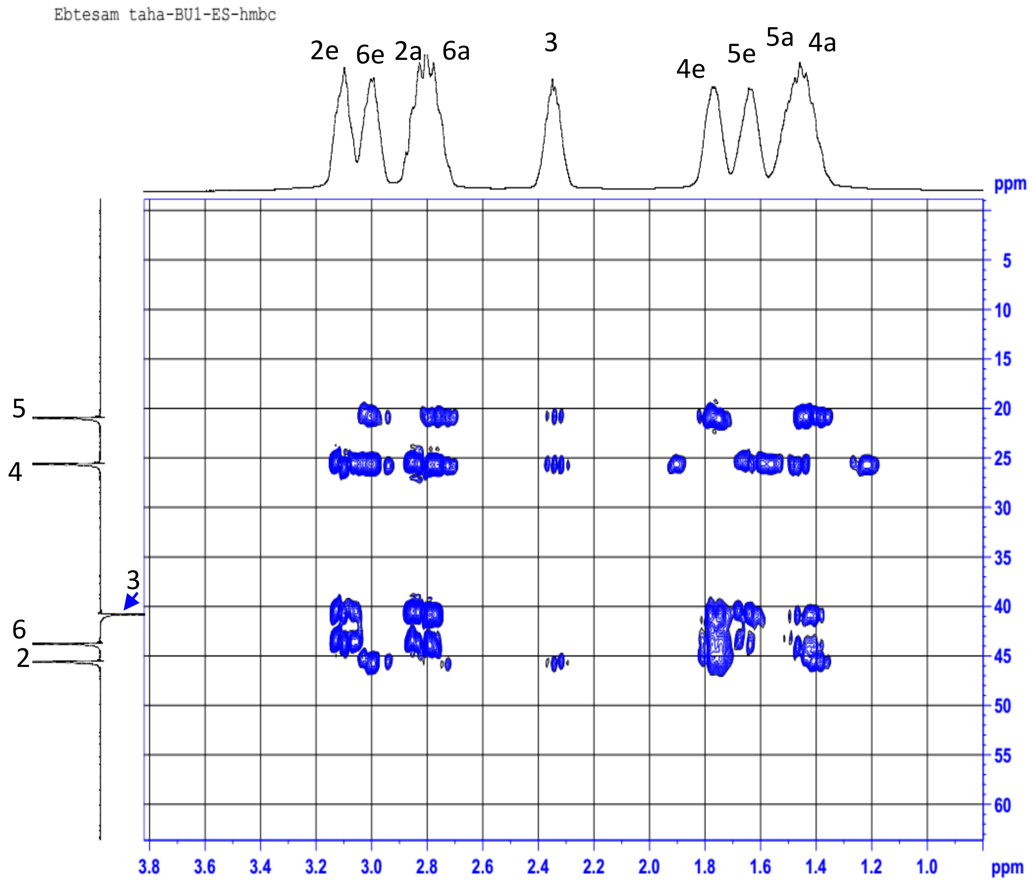

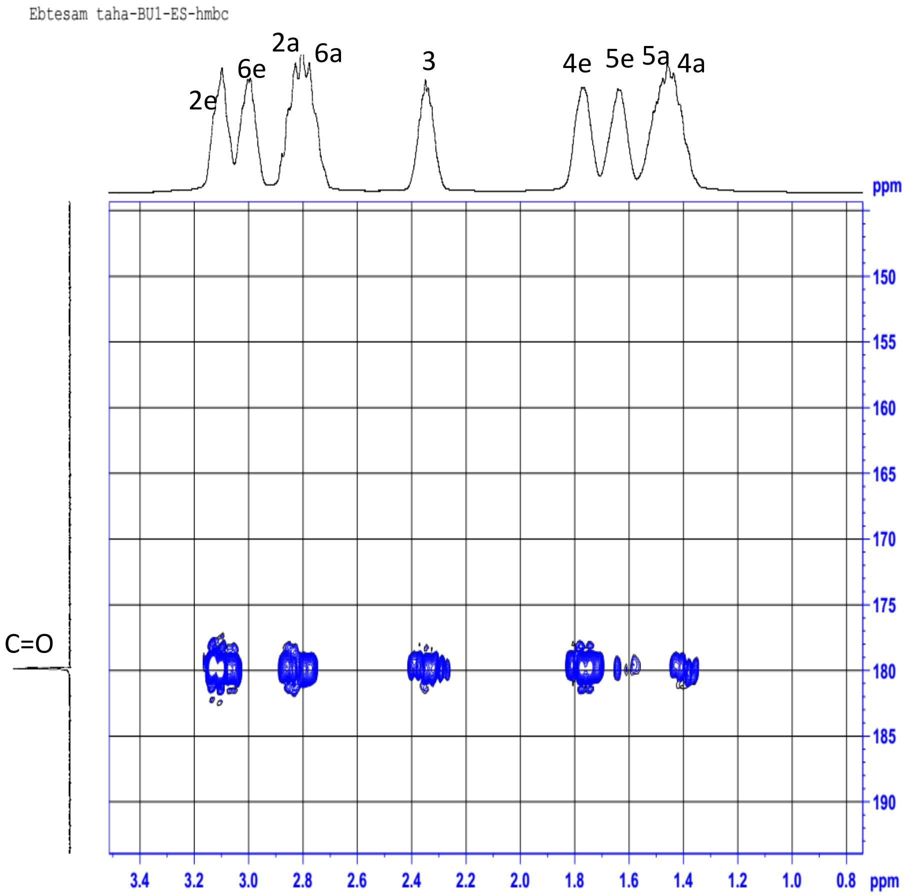

Fig. S21: HMBC spectrum of **3** (D_2O).

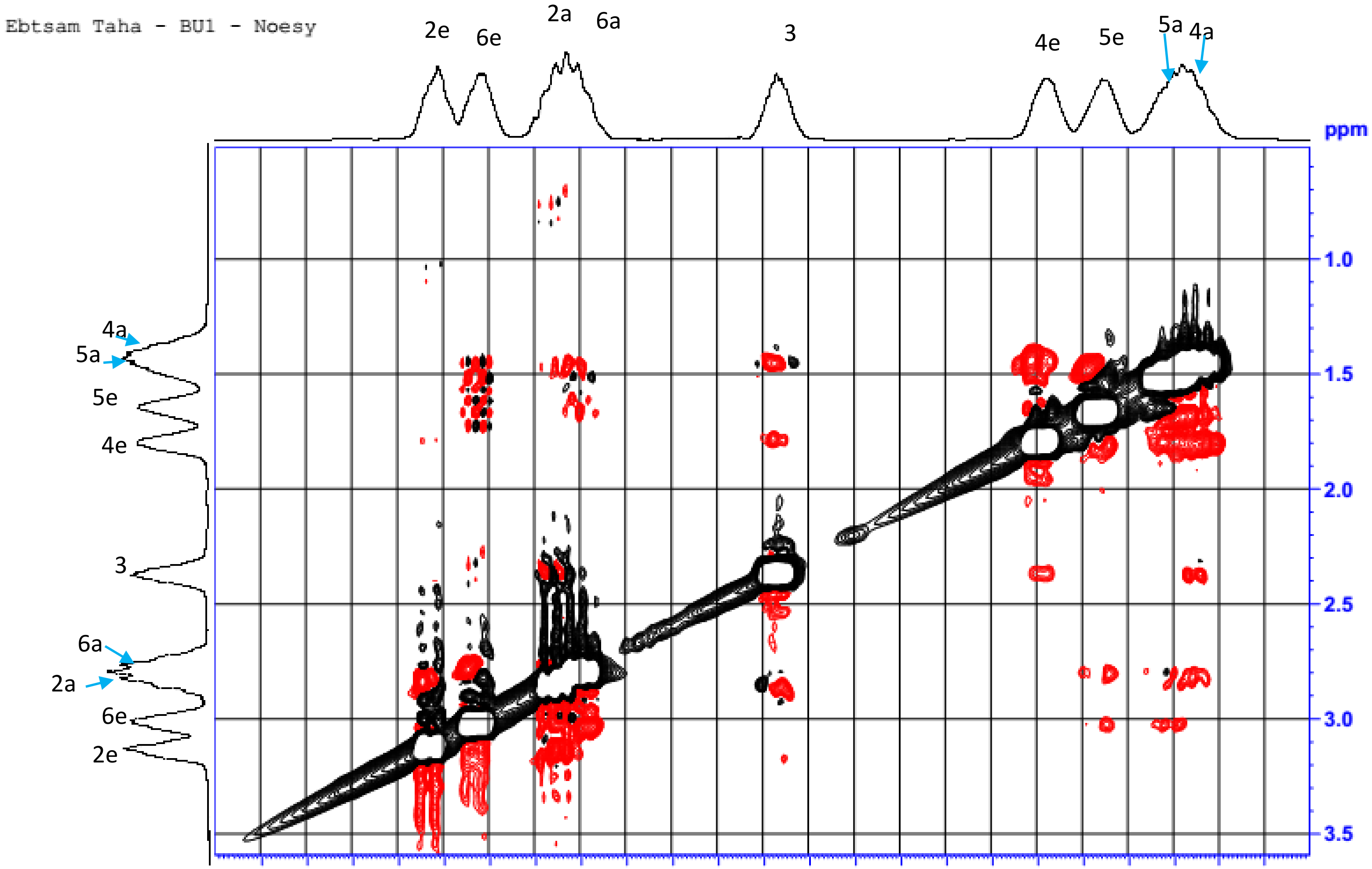

Fig. S22: NOE spectrum of **3** (D_2O).

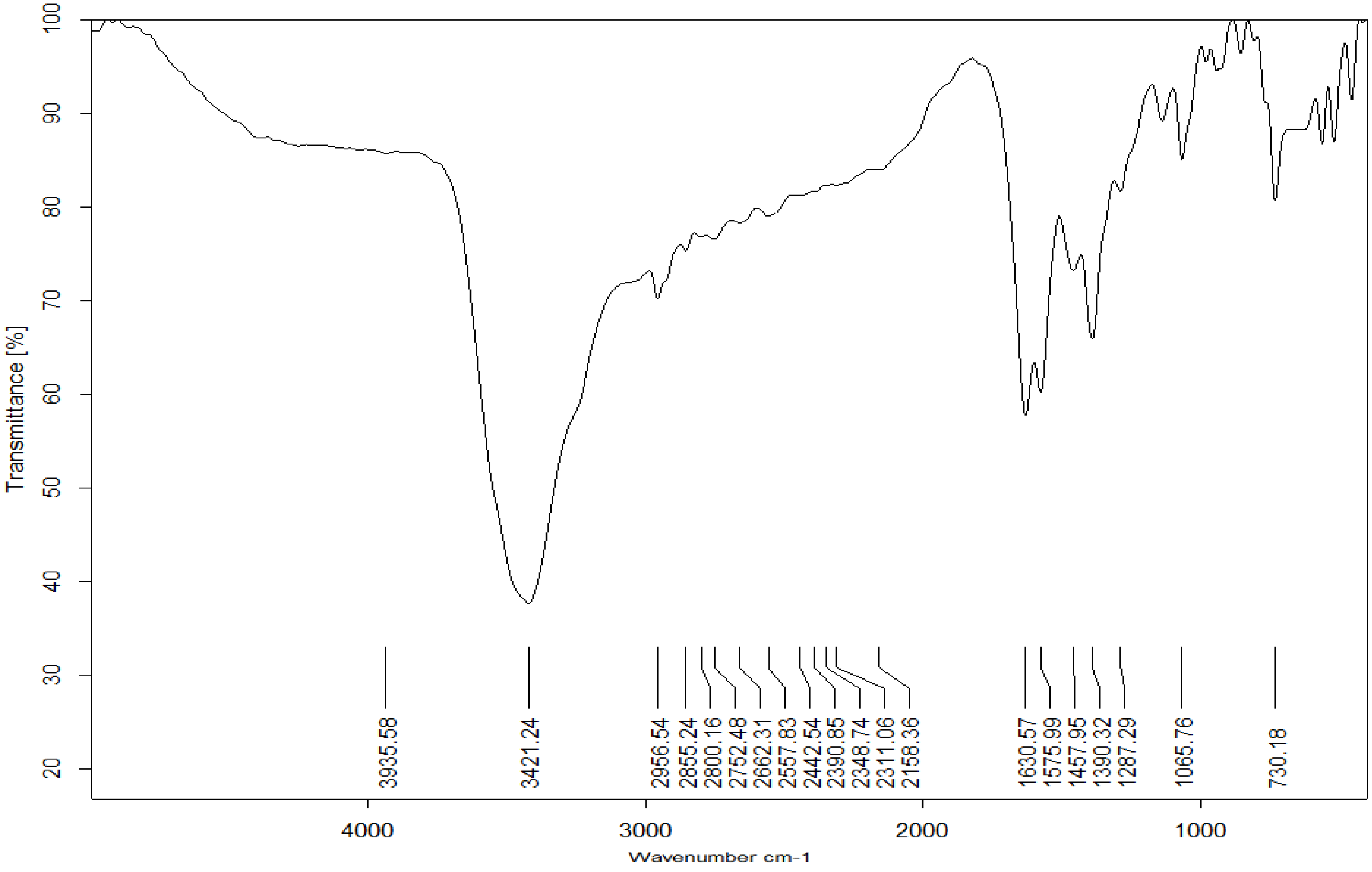

Fig. S23: IR spectrum of **3** (KBr disc).

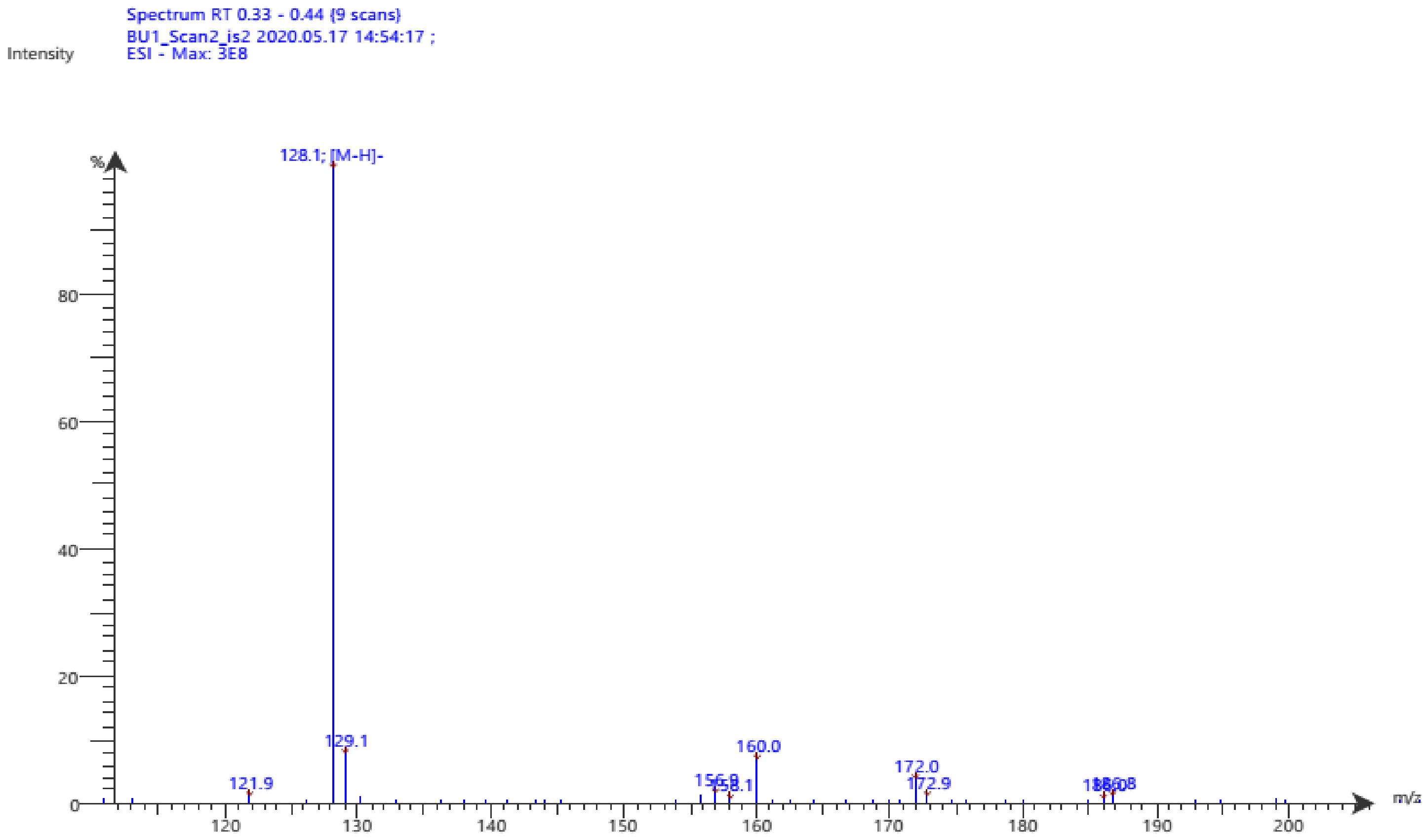

Fig. S24: ESIMS (-ve) spectrum of **3**.

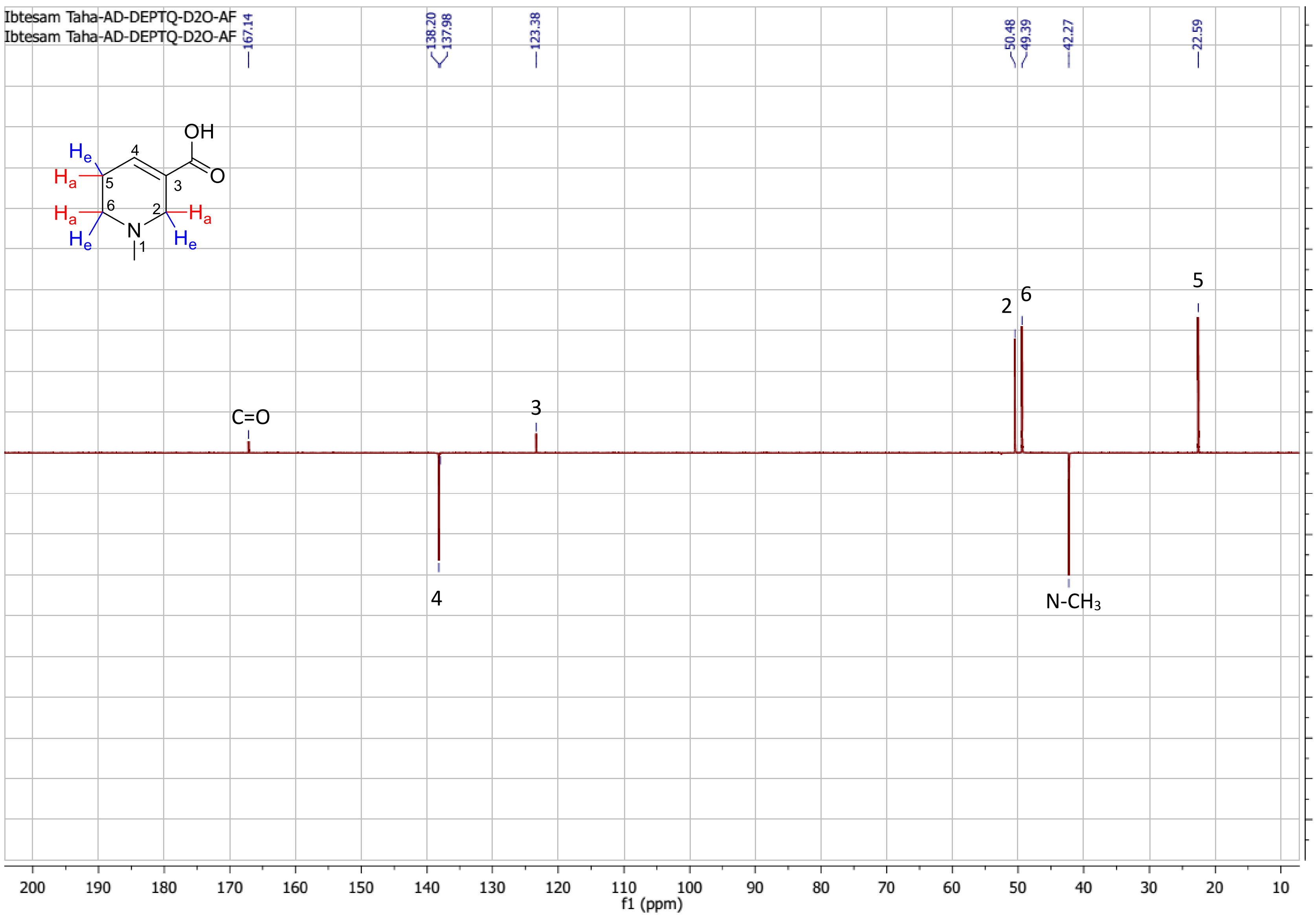

Fig. S25: DEPTQ-135 spectrum of **4** (D_2O, 100 MHz).

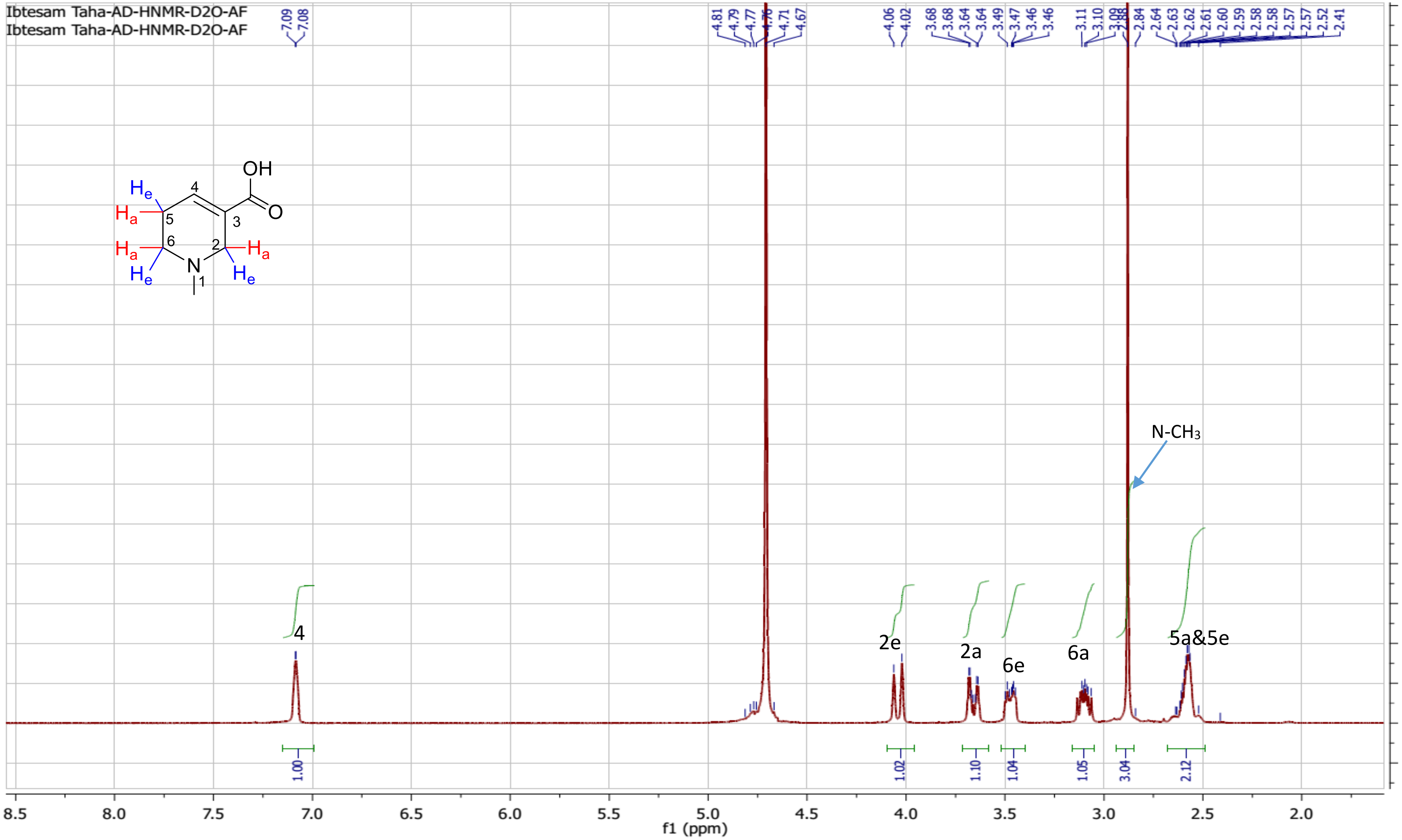

Fig. S26: ^{1}H NMR spectrum of **4** (D_2O, 400 MHz).

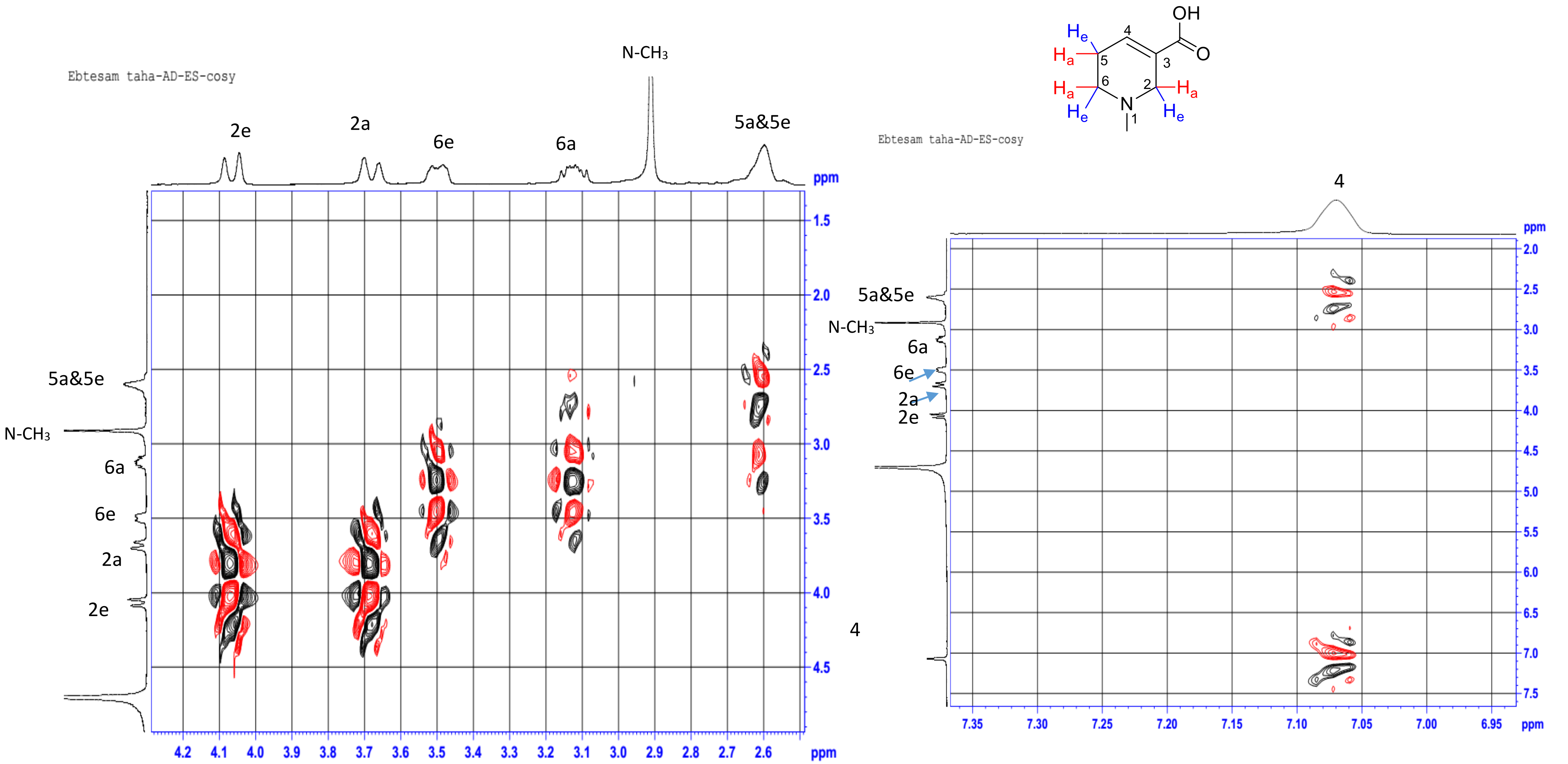

Fig. S27: COSY spectrum of **4** (D_2O).

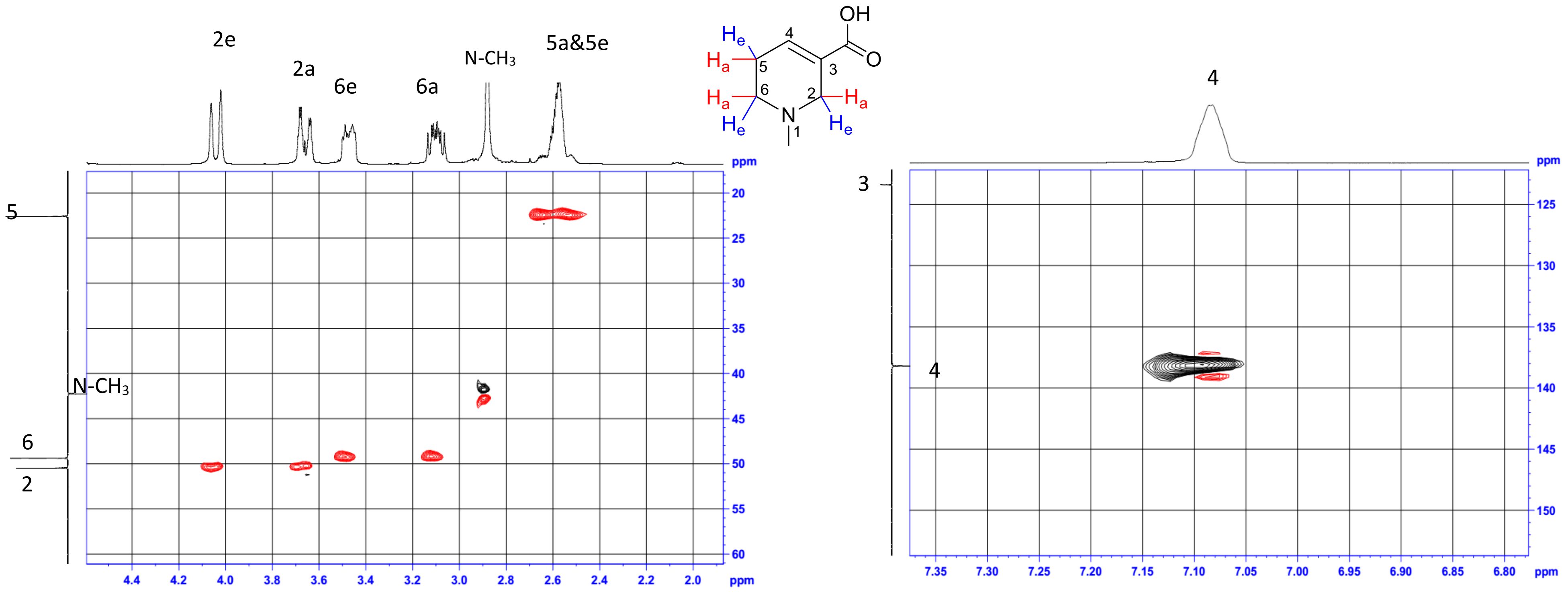

Fig. S28: DEPTQ-135 and ^{1}H NMR correlation spectrum of **4** (D_2O).

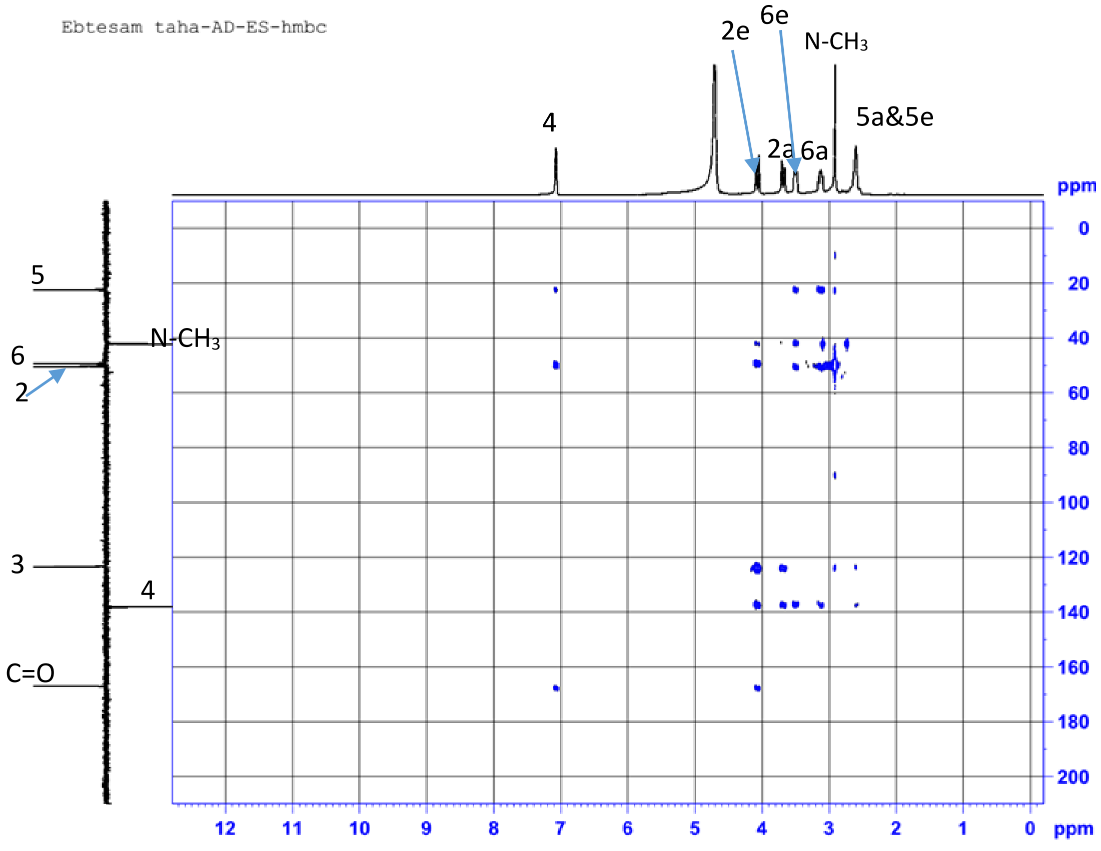

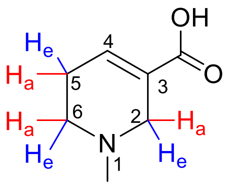

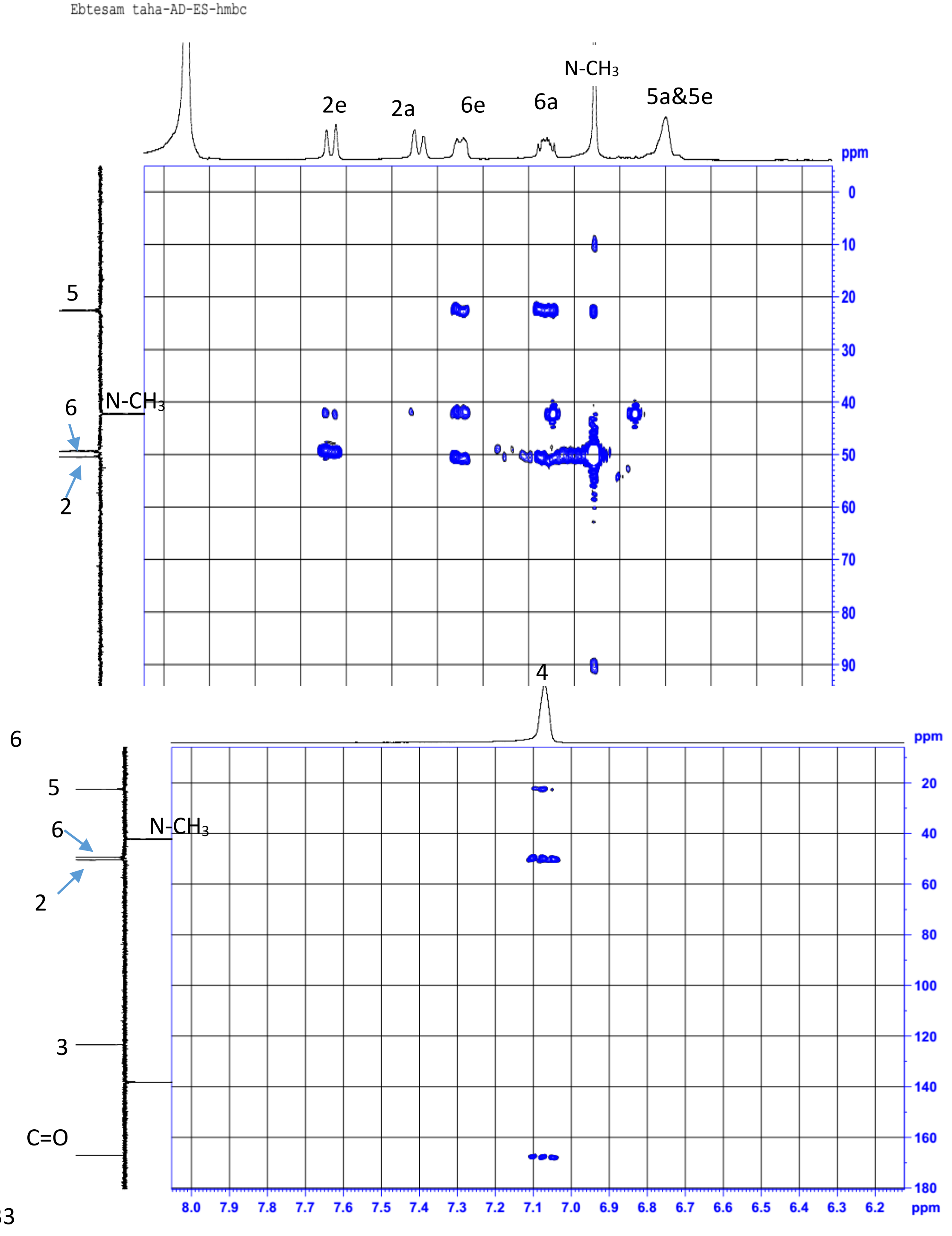

Fig. S29: HMBC spectrum of **4** (D_2O).

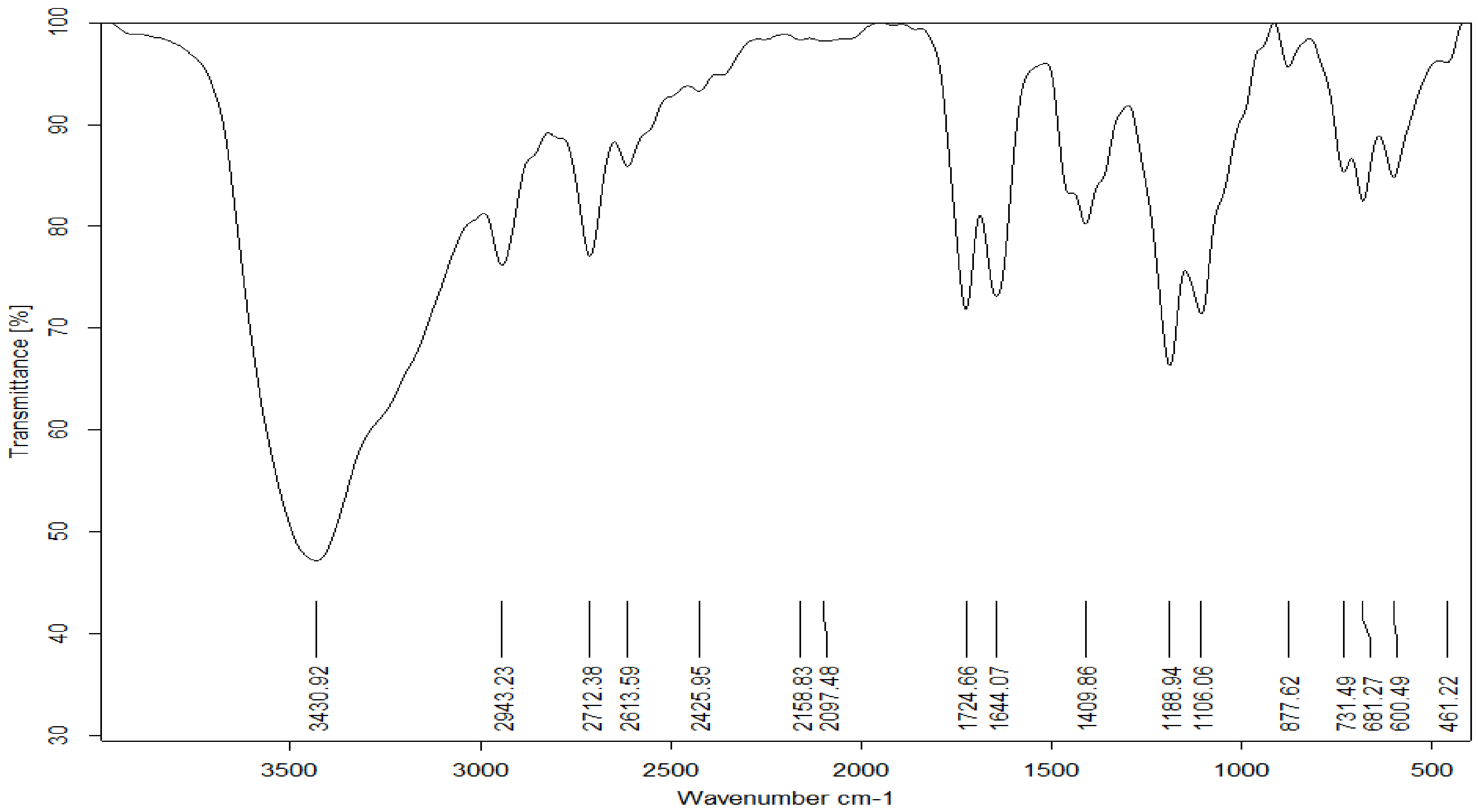

Fig. S30: IR spectrum of **4** (KBr disc).

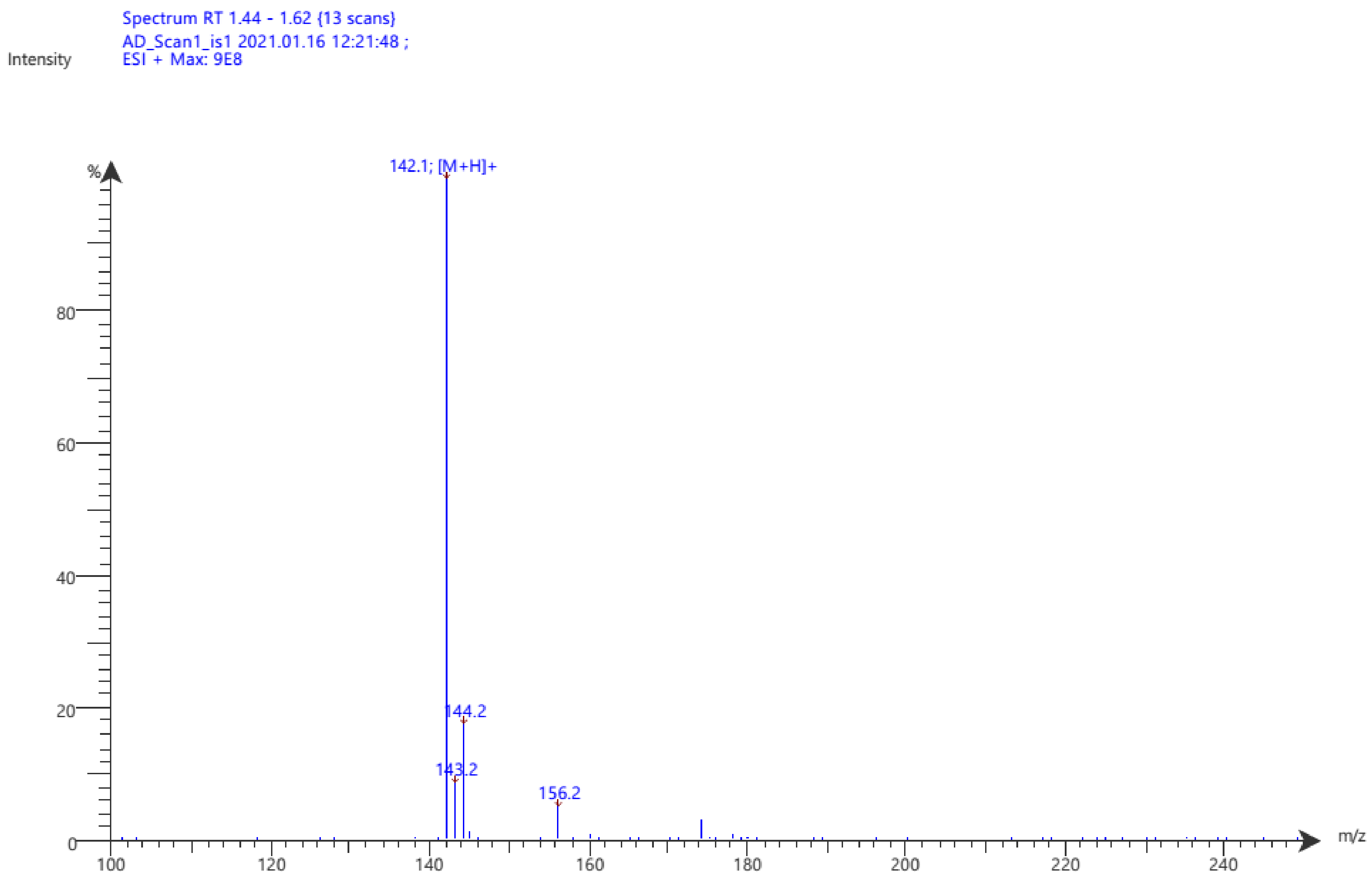

Fig. S31: ESIMS (+ve) spectrum of **4**.

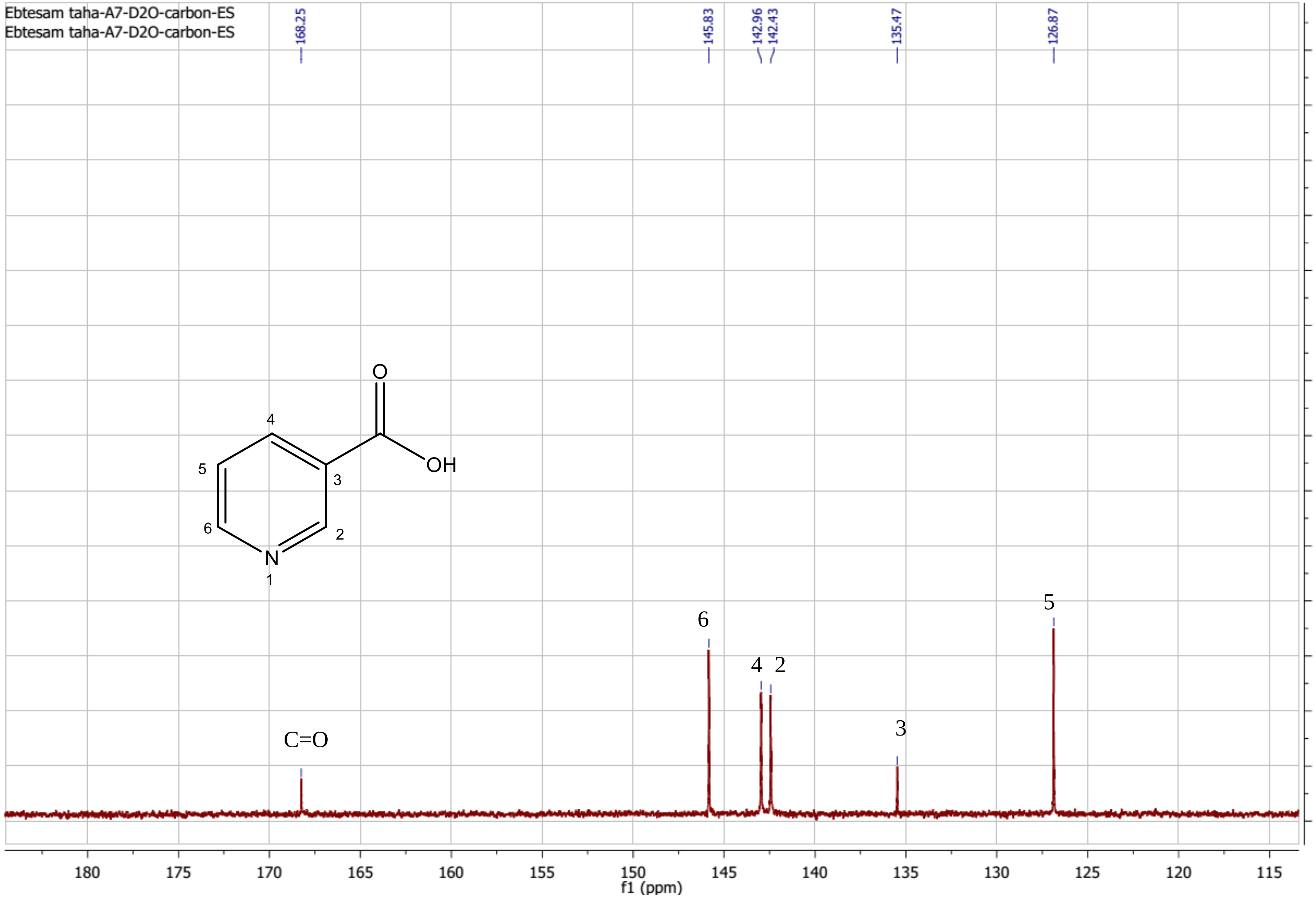

Fig. S32: ^{13}C NMR spectrum of **5** (D_2O, 100 MHz).

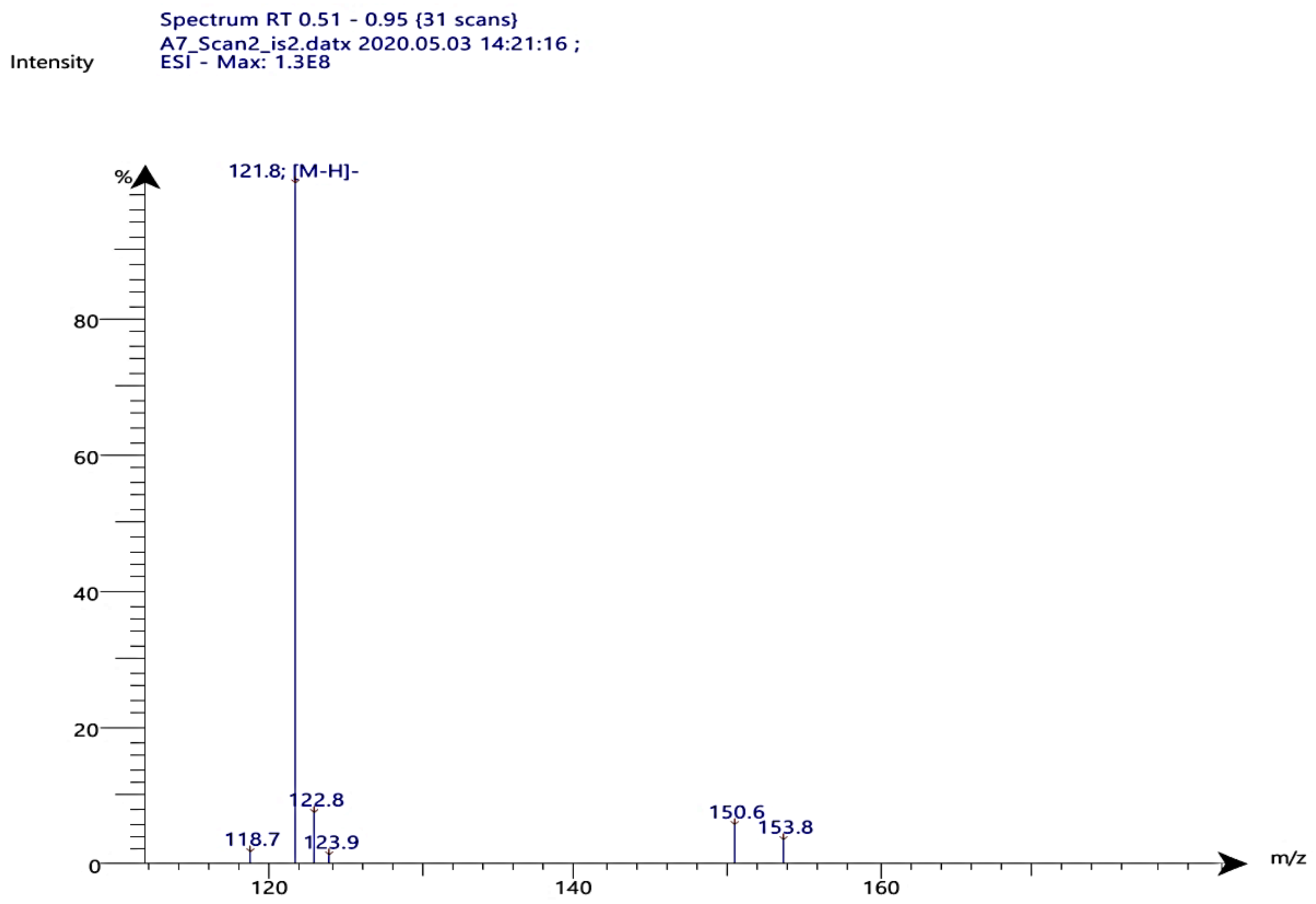

Fig. S33: ESIMS (-ve) spectrum of **5.**

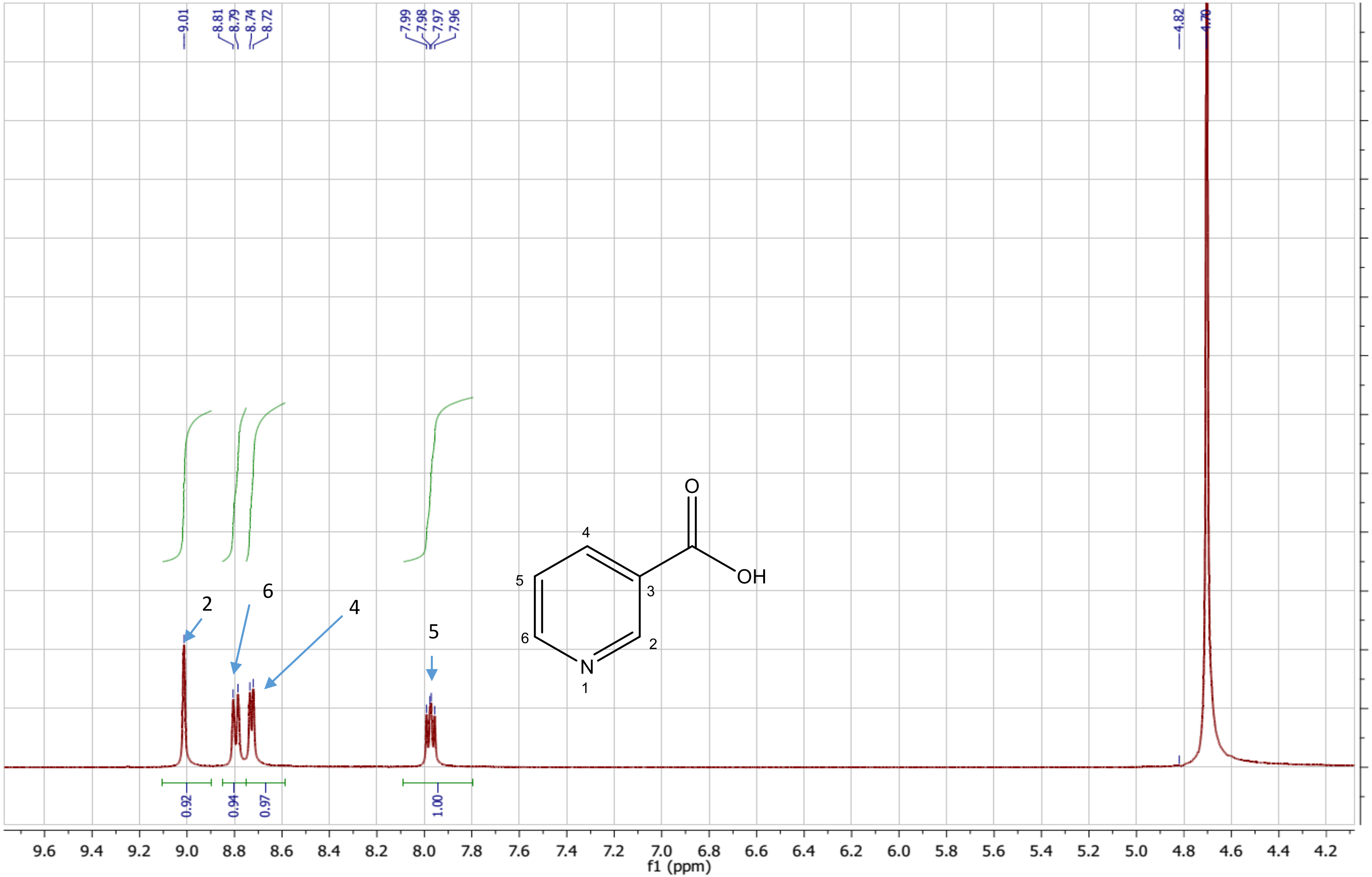

Fig. S34: ^{1}H NMR spectrum of **5** (D_2O, 400 MHz).

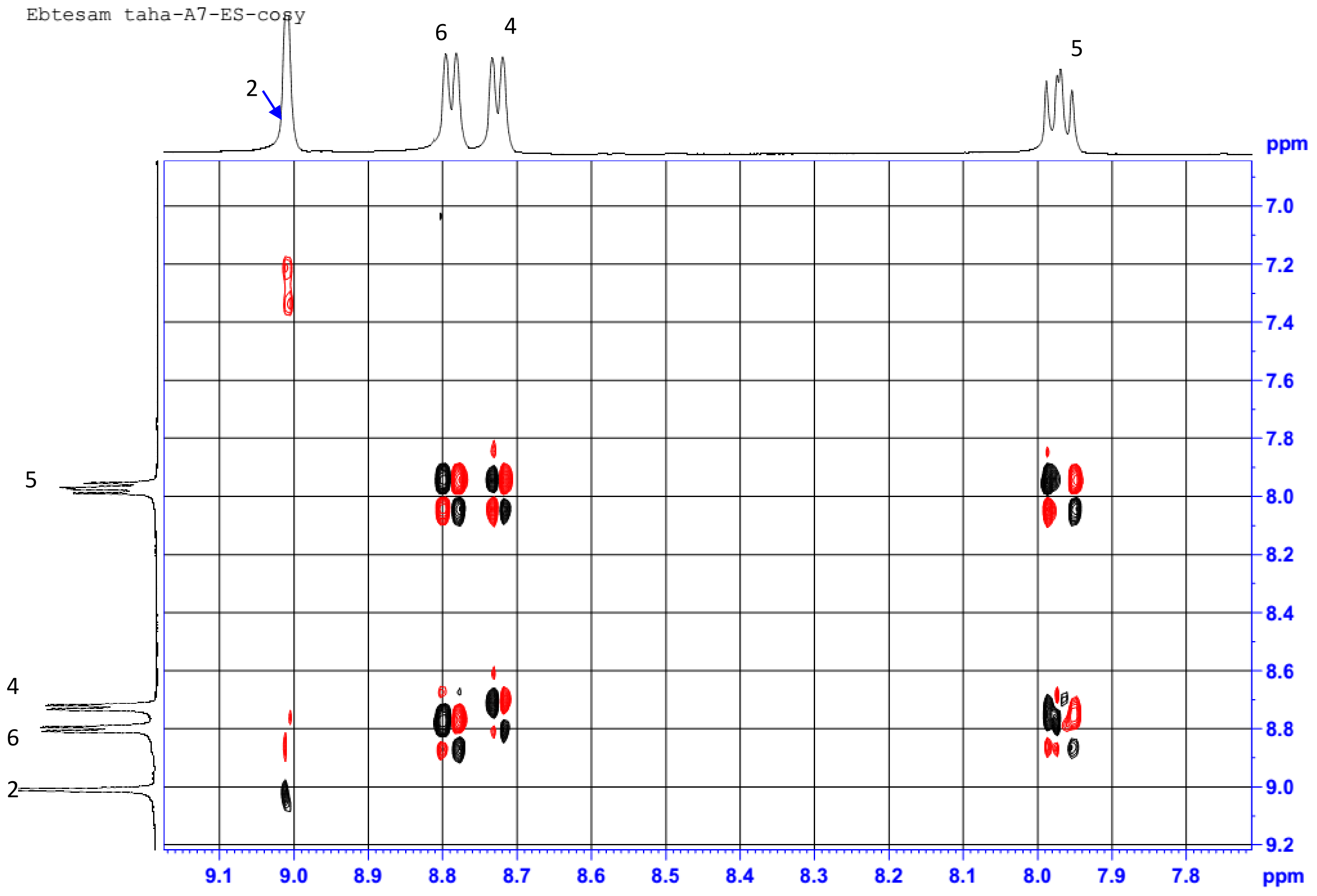

Fig. S35: COSY spectrum of **5** (D_2O).

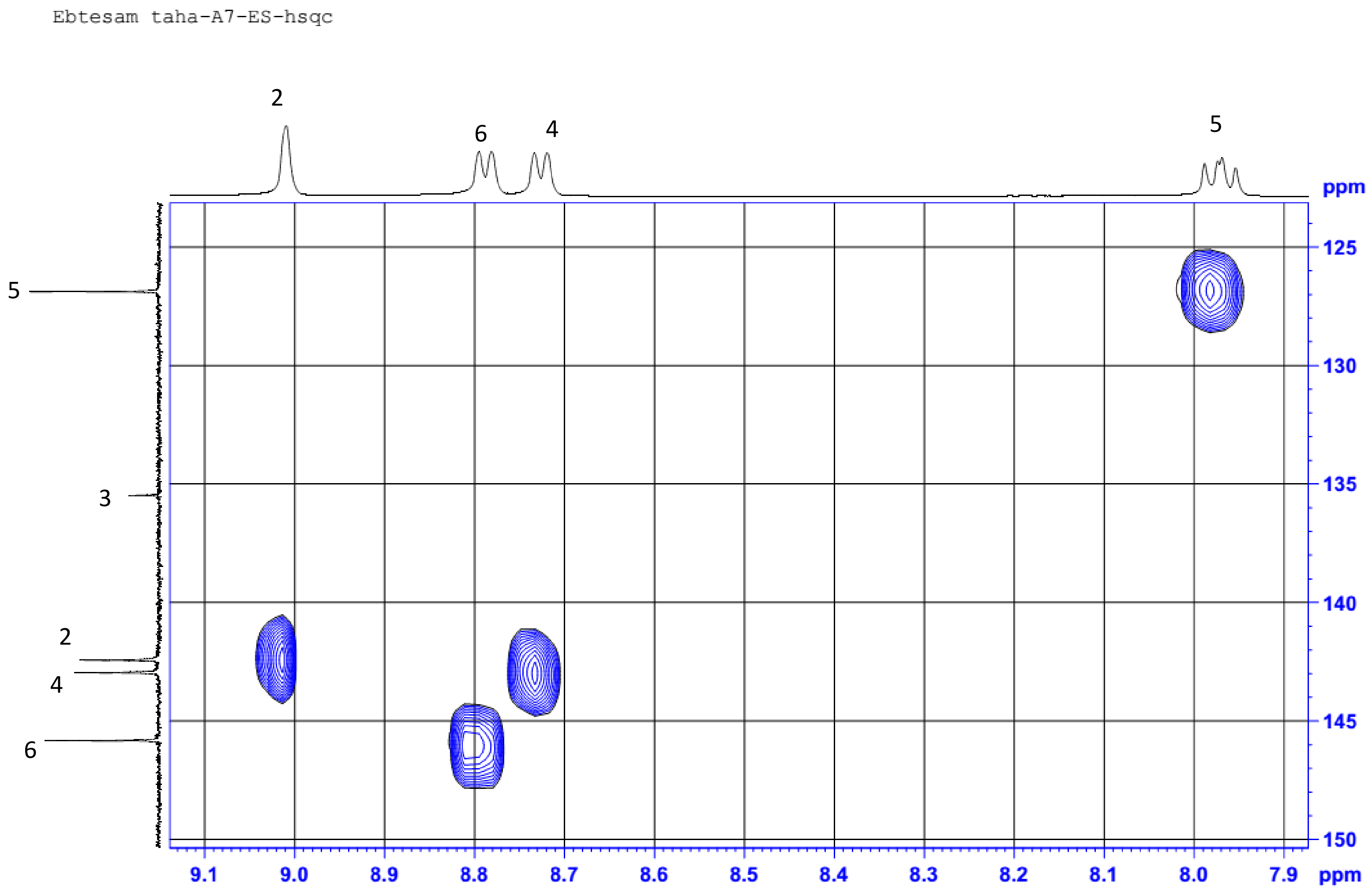

Fig. S36: HSQC spectrum of **5** (D_2O).

Ebtesam taha-A7-ES-hmbc

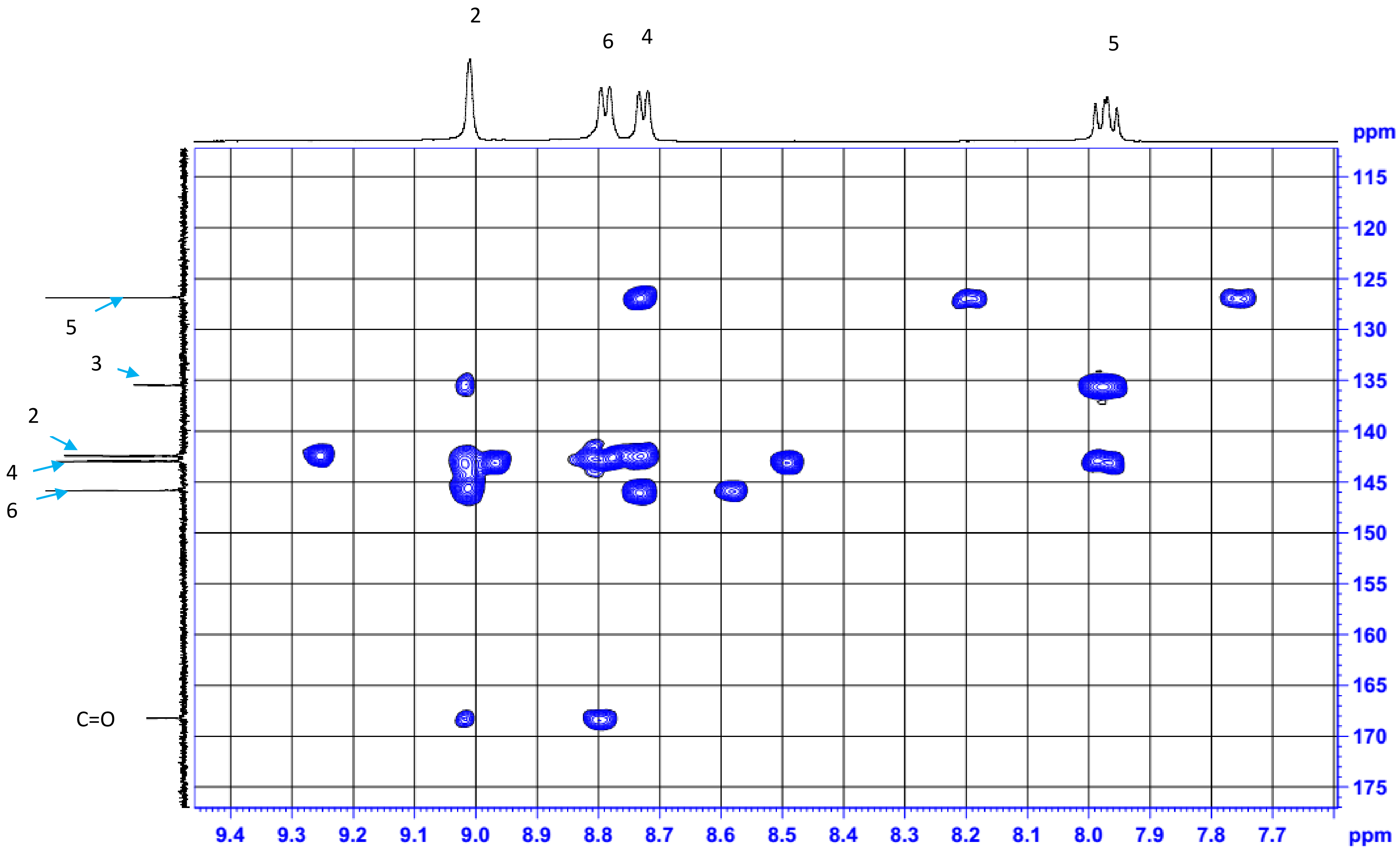

Fig. S37: HMBC spectrum of **5** (D_2O).

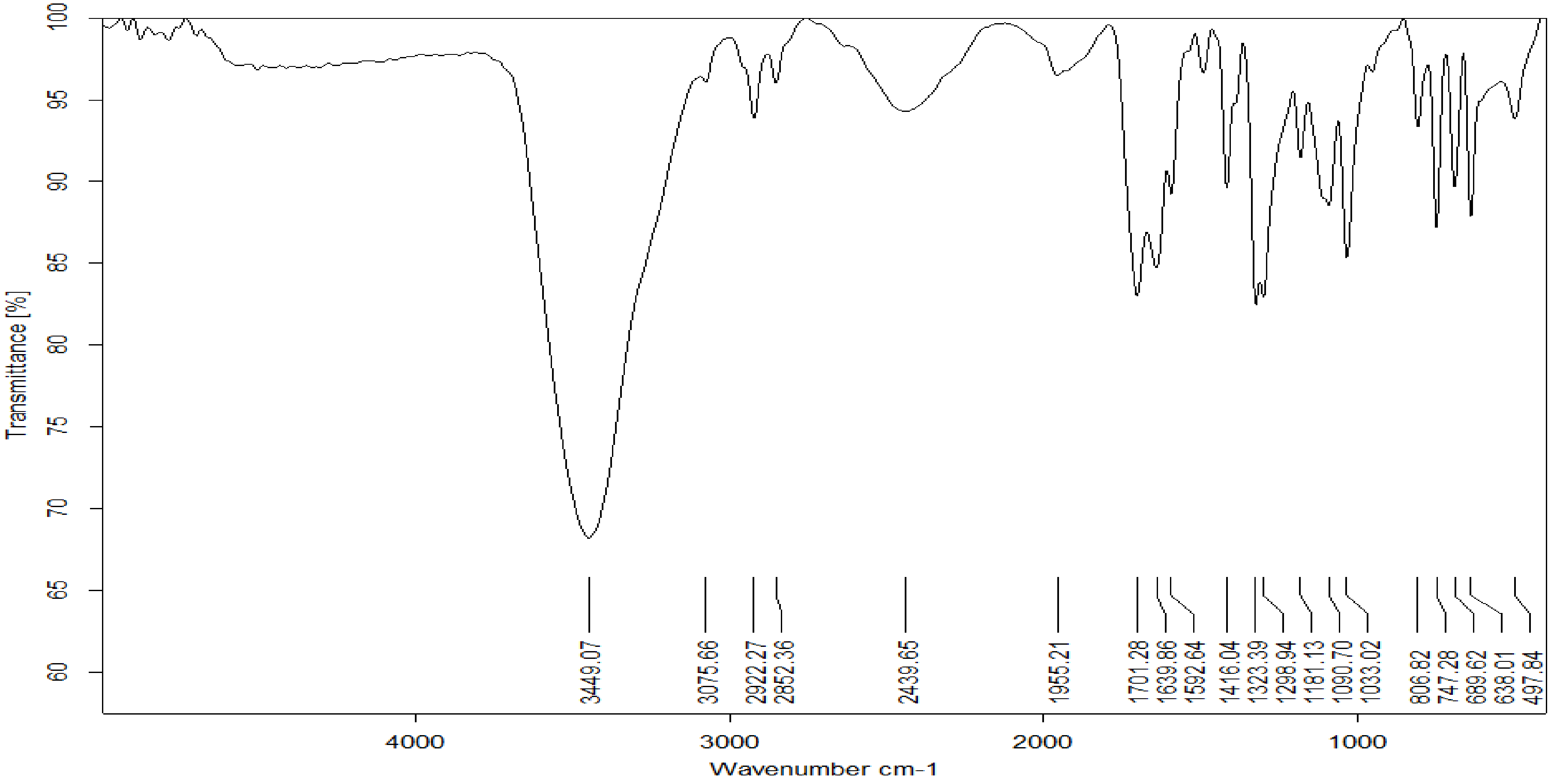

Fig. S38: IR spectrum of **5** (KBr disc).

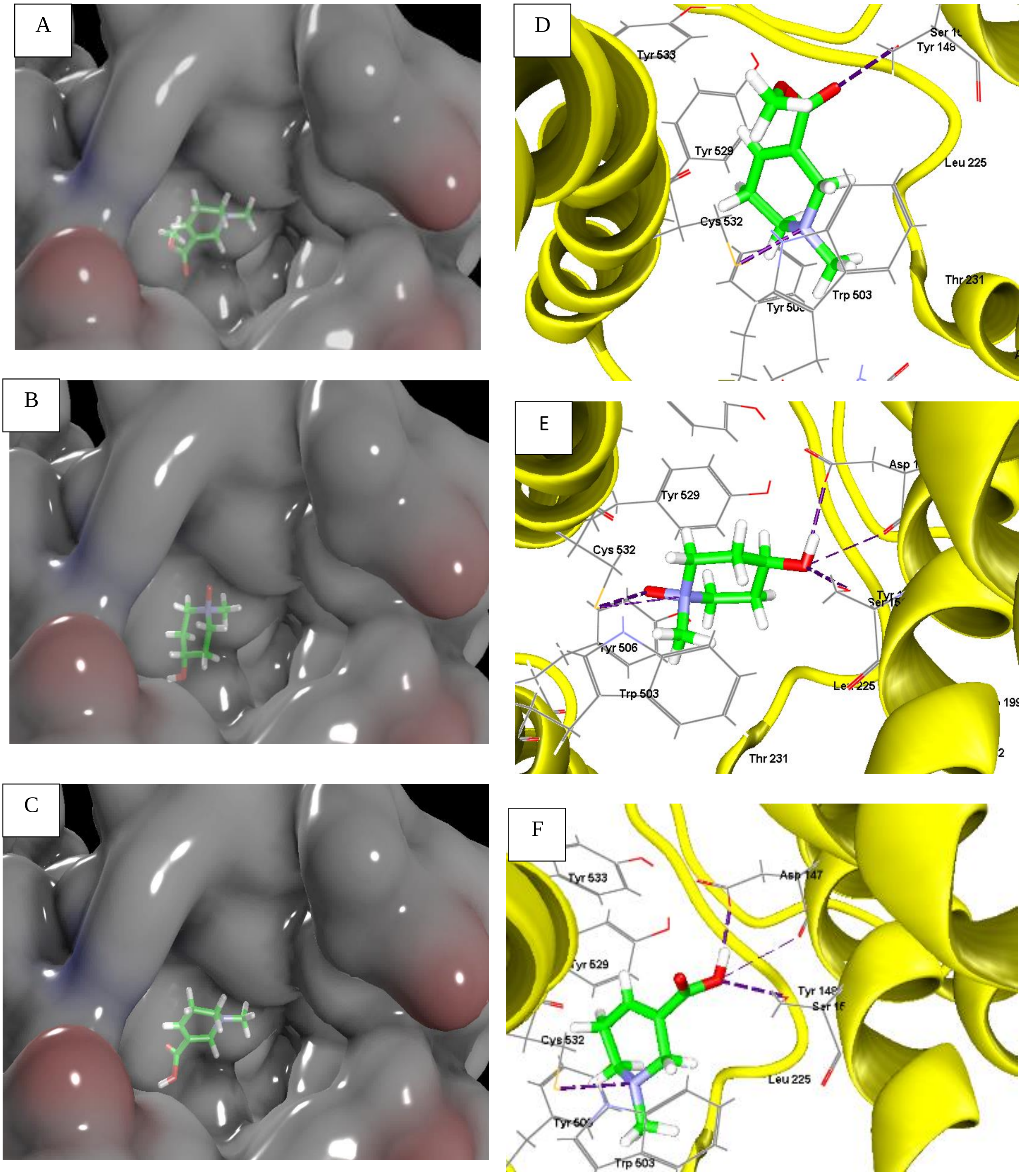

Fig. S39: The binding mode of **arecoline**, **2** and **4**. Electrostatic surface representation of ligand binding pocket of **arecoline** (A), **2** (B) and **4** (C). Amino acid residues involved in ligand interaction, hydrogen bonds are shown as blue dashed lines, **arecoline** (D), **2** (E) and **4** (F).

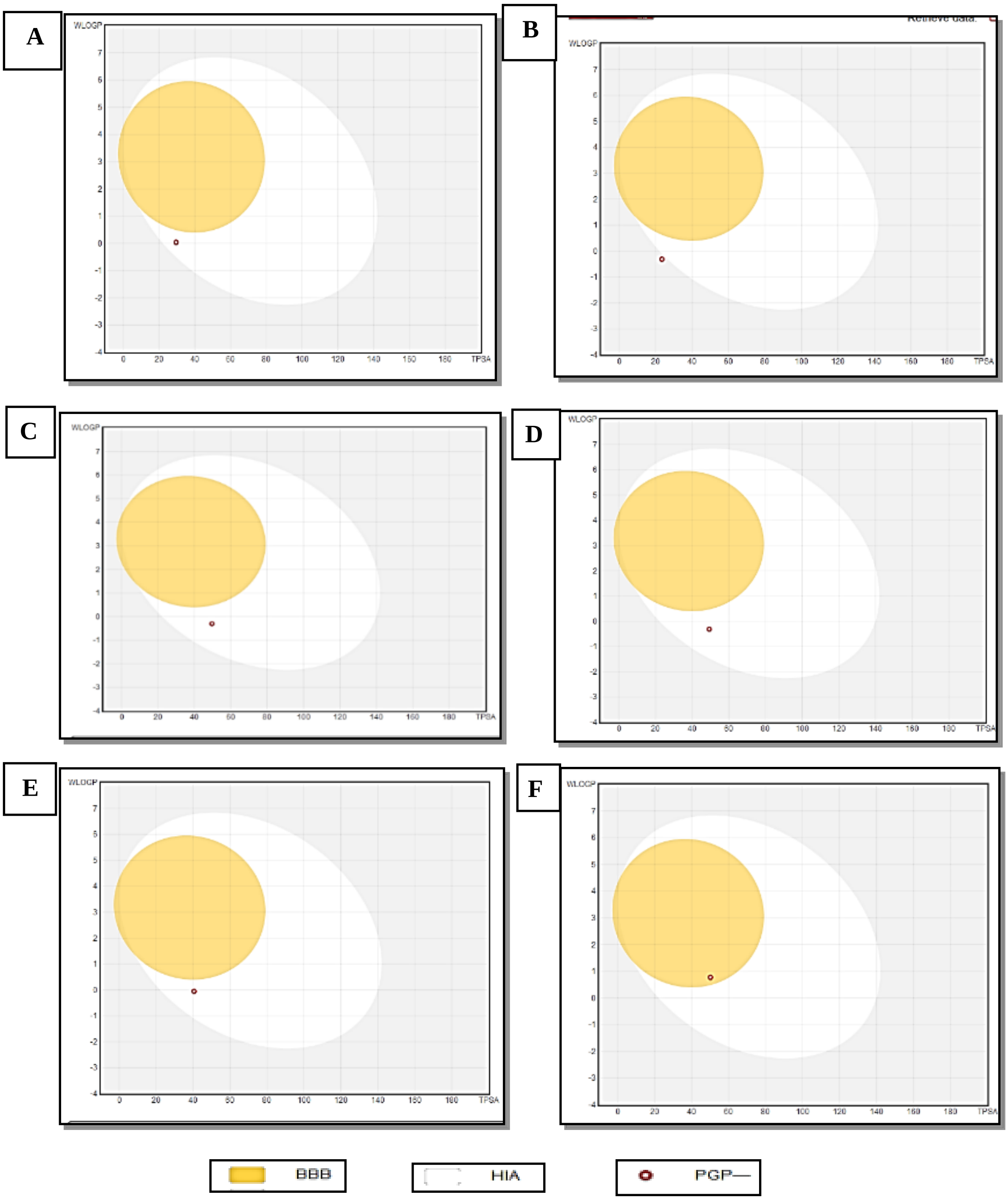

Fig. S40: SwissADME prediction, BOILED egg showing blood brain barrier permeability (yellow region) and human intestinal absorption (white region). Red dots represented molecules predicted not to be effluated from central nervous system by P-glycoprotein. **Arecoline** (A), **1** (B)**, 2** (C), **3** (D), **4** (E) and **5** (F).

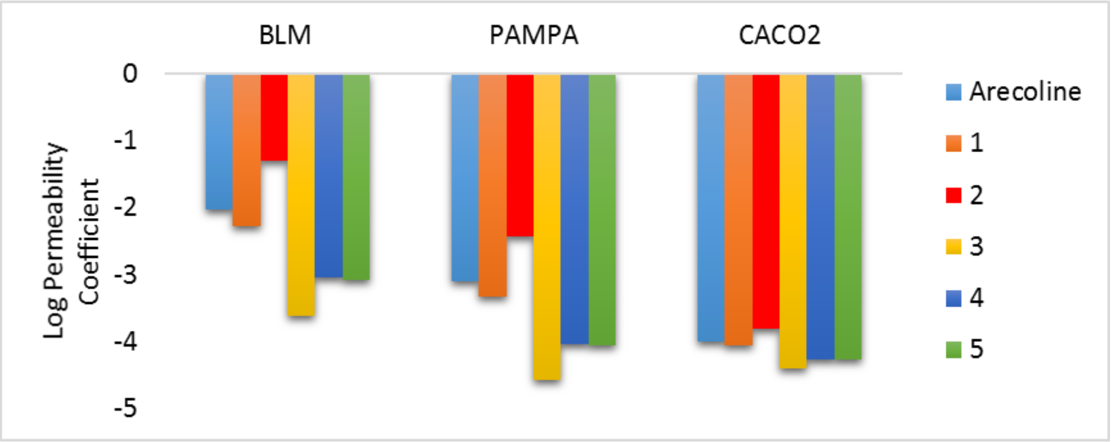

Fig. S41: Cell permeability prediction of arecoline and compounds **1-5** from PerMM web-based tool.

Table S1: A: Effect of **1** on the cell cycle of non-small cell lung cancer A549 using untreated lung cancer cells as a negative control. **B:** Effect of **3** and **5** on cell cycle of leukemia cancer K562 using untreated leukemia cancer cells as a negative control.

Tested compounds	Cell cycle distribution (DNA content)			
	**%Pre-G1**	**%G0-G1**	**%S**	**%G2/M**
**A) Lung cancer A549 cell line**				
**1**	28.61	44.11	31.01	24.88
Untreated lung A549 (**Control**)	1.26	56.06	35.85	8.09
**(B) Leukemia cancer K562 cell line**				
**3**	43.22	35.29	57.23	7.48
**5**	31.74	36.41	52.91	10.68
Untreated leukemia K562 (**Control**)	1.68	44.84	42.57	12.59

Table S2: **A**: Detection of apoptosis induced by **1** using non-small cell lung cancer A549 and untreated cells as a negative control. **B**: Detection of apoptosis induced by **3** and **5** using leukemia cancer K562 and untreated cells as a negative control. Fold increase than control was included.

Test compound	% DNA content			
	Total	Apoptosis		Necrosis
		Early	Late	
**(A) Lung cancer A549 cell line**				
**1**	28.61	5.09	10.38	13.14
Untreated lung A549 (Control)	1.26	0.29	0.18	0.79
Fold increase by **1**	22.71	17.55	57.67	16.63
**(B) Leukemia cancer K562 cell line**				
**3**	43.22	2.33	30.35	10.54
**5**	31.74	2.94	11.74	17.06
Untreated leukemia K562 (Control)	1.68	0.37	0.04	1.27
Fold increase by **3**	25.73	6.30	758.75	8.30
Fold increase by **5**	18.89	7.95	293.5	13.43

Table S3: Docking results of arecoline and compounds **1-5** to M3-mT4L (PDB ID: 4U15).

Ligand	MolDock Score (kcal/mol)	Rerank Score (kcal/mol)	Interaction (kcal/mol)	H. Bond (kcal/mol)	LE1[a]	LE3
Arecoline	-81.15	-67.15	-85.54	-3.50	-7.38	-6.10
**1**	-62.19	-54.32	-66.54	-4.59	-7.77	-6.79
**2**	-73.43	-61.62	-77.08	-9.15	-8.16	-6.85
**3**	-66.20	-57.88	-71.20	-2.29	-7.35	-6.43
**4**	-75.37	-63.87	-79.63	-4.91	-7.54	-6.39
**5**	-65.18	-51.62	-71.77	-5.86	-7.24	-5.74

[a] LE: Ligand efficiency

Table S4: Amino acids involved in hydrogen bonding interaction of arecoline and compounds **1-5** with M3-mAChR.

Ligand name	Amino acids involved in hydrogen bonding
Arecoline	Ser 151, Cys 532
**1**	Ser 151, Asp 147
**2**	Ser 151, Cys 532, Asp 147
**3**	Ser 151, Tyr 529, Asp 147
**4**	Ser 151, Cys 532, Asp 147
**5**	Ser 151, Cys 532, Asp 147

Table S5: Physicochemical properties of arecoline and compounds **1-5** for detection of drug likeness and oral bioavailability.

Tested compound	Size (M.W)[a]	Flexibility (number of rotatable bonds)	Hydrogen bond donor	Hydrogen bond acceptor	Polarity (TPSA)[b]	Partition coefficient WLogp
**Arecoline**	155.19	2	0	2	29.54	0.04
**1**	115.17	0	1	2	23.47	-0.31
**2**	131.13	1	1	2	49.66	-0.30
**3**	129.16	1	2	3	49.33	-0.31
**4**	141.17	1	1	3	40.54	-0.05
**5**	123.11	1	1	3	50.19	0.78

[a]M.W: Molecular weight. [b]TPSA: Topological polar surface area.

Table S6: Results of ADME prediction of arecoline and compounds **1-5** from PreADMET web-based tool.

Tested compound	ADME prediction	
	Absorption	Distribution
	Caco-2[a] cell permeability (nm/sec)	%PPB[b]
Arecoline	26.33	8.14
**1**	37.30	0
**2**	6.71	14.13
**3**	20.74	0
**4**	21.02	91.95
**5**	20.75	4.51

[a]Caco-2: Colorectal carcinoma.

[b]%PPB: Plasma protein binding.

Table S7: Cell permeability prediction of arecoline and compounds **1-5** using PerMM web-based tool.

Log of permeability Coefficient (kcal/mol)	**Arecoline**	**1**	**2**	**3**	**4**	**5**
BLM [a]	-2.02	-2.27	-1.29	-3.61	-3.04	-3.07
PAMPA [b]	-3.09	-3.33	-2.42	-4.56	-4.03	-4.06
Caco-2 [c]	-4.00	-4.06	-3.81	-4.4	-4.26	-4.27

[a] BLM: Black liquid membrane.

[b] PAMPA: Parallel artificial membrane permeability assay.

[c] Caco-2: Colorectal carcinoma cell.

Table S8: List of microorganisms.

	Microorganism	Code	Family
1	*Alternaria alternata*	AUMC 4685	Pleosporaceae
2	*Aspergillus flavipes*	ATCC 11013	Trichocomaceae
3	*A. ochraceous*	NRRL 405	Trichocomaceae
4	*A. flavus*	AUMC 4787	Trichocomaceae
5	*A. fumigatus*	**	Trichocomaceae
6	*A. niger*	ATCC 10549	Trichocomaceae
7	*A. niger*	AUMC 4156	Trichocomaceae
8	*A. niger*	NRRL 328	Trichocomaceae
9	*A. versicolor*	AUMC 4807	Trichocomaceae
10	*Candida albicans* (lab isolate)	*	Saccharomycetaceae
11	*Chaetomium funicolum*	**	Chaetomiaceae
12	*Cladosporium species*	**	Davidiellaceae
13	*Cunninghamella blakesleeana*	MR 198	Cunninghamellaceae
14	*C. blakesleeana*	NRRL 1369	Cunninghamellaceae
15	*C. echinulata*	NRRL 1382	Cunninghamellaceae
16	*C. elegans*	NRRL 1392	Cunninghamellaceae
17	*C. elegans*	NRRL 2310	Cunninghamellaceae
18	*Dreshella species*	**	Pleosporaceae
19	*Gymnascella citrina*	NRRL 6050	Gymnoascaceae
20	*Lindra pennispora*	NRRL 2237	Lulworthiaceae
21	*Mucor species*	**	Mucoraceae
22	*Penicillium chrysogenium*	ATCC 10002	Trichocomaceae
23	*P. chrysogenium*	ATCC 9480	Trichocomaceae
24	*P. glabrum*	**	Trichocomaceae
25	*P. brevicompactum*	AUMC 2751	Trichocomaceae
26	*P. vermiculatum*	NRRL 1009	Trichocomaceae
27	*Rhizopus somniferum*	ATCC 36060	Mucoraceae
28	*Rhodotorula rubra*	NRRL y 1592	Sporidiobolaceae
29	*Saccharomyces cerivisae (Baker's yeast)*	Lyophilized yeast cell	Saccharomycetaceae
30	*Trichoderma virdie*	**	Hypocreaceae
31	*Ulocladium botrytis*	**	Pleosporaceae

* Laboratory isolate supplied through the Department of Microbiology, College of Pharmacy, King Saud University.

**Isolate obtained from Assiut University Mycological Center (AUMC).
